# Characteristics of older patients undergoing surgery in the UK: SNAP-3, a snapshot observational study

**DOI:** 10.1016/j.bja.2024.11.024

**Published:** 2025-01-06

**Authors:** Claire Jane Swarbrick, Karen Williams, Bob Evans, Helen Abigail Blake, Thomas Poulton, Samuel Nava, Akshay Shah, Peter Martin, Judith Stephanie Louise Partridge, Iain Keith Moppett, SNAP-3 collaborators

**Affiliations:** 1 Anaesthesia, Royal Devon University Healthcare NHS Foundation Trust, Exeter, UK; 2 Centre for Research and Improvement, Royal College of Anaesthetists, London, UK; 3 Patient, Carer and Public Involvement and Engagement (PCPIE) Group, Royal College of Anaesthetists, London, UK; 4 Department of Primary Care and Population Health, University College London, London, UK; 5 Department of Anaesthesia, Perioperative Medicine, and Pain Medicine, Peter MacCallum Cancer Centre, Melbourne, VIC, Australia; 6 Department of Critical Care, University of Melbourne, Melbourne, VIC, Australia; 7 Research Department of Targeted Intervention, University College London, London, UK; 8 Royal United Hospitals Bath NHS Foundation Trust, Bath, UK; 9 Nuffield Department of Clinical Neurosciences, University of Oxford, Oxford, UK; 10 Department of Anaesthesia, Hammersmith Hospital, Imperial College Healthcare NHS Trust, London, UK; 11 Perioperative Medicine for Older People Undergoing Surgery (POPS), Guy's and St Thomas' NHS Foundation Trust, London, UK; 12 School of Population Health and Environmental Sciences, Faculty of Life Sciences and Medicine, King's College Hospital, London, UK; 13 Anaesthesia a Critical Care, Injury, Recovery and Inflammation Sciences, University of Nottingham, Nottingham, UK

**Keywords:** ageing, epidemiology, frailty syndrome, geriatric medicine, multimorbidity, perioperative care, surgery

## Abstract

**Background:**

Frailty and multimorbidity are common in older adults, but the prevalence and interaction of these conditions in surgical patients remain unclear. This study describes the clinical characteristics of a heterogeneous cohort of older UK surgical patients.

**Methods:**

We conducted a prospective observational cohort study during 5 days in March 2022, aiming to recruit all UK patients aged 60 yr and older undergoing surgery, excluding minor procedures (e.g. cataract surgery). Data were collected on patient characteristics, clinical care, frailty, and multimorbidity measures.

**Results:**

A total of 7134 patients from 214 NHS hospitals were recruited, with a mean (sd) age of 72.8 (8.1) yr. Of all operations, 69% (95% confidence interval [CI] 67.9–70.1%) were elective, and 34% (95% CI 32.7–34.8%) were day cases. Of the patients, 19% (95% CI 18.3–20.1%) were living with frailty (Clinical Frailty Score ≥5), and 63.1% (95% CI 62.0–64.3%) were living with multimorbidity (count of ≥2 comorbidities). Those living with frailty, multimorbidity, or both were typically older, were from lower socioeconomic backgrounds, and experienced greater polypharmacy and reduced independence. Patients living with frailty were less likely to undergo elective and day-case surgeries. Four out of five (78.8% [1079/1369]) of those who were living with frailty were also living with multimorbidity; 27.1% (1079/3978) of those who were living with multimorbidity were also living with frailty.

**Conclusions:**

In the UK, one in five older patients undergoing surgery is living with frailty, and almost two-thirds of older patients are living with multimorbidity. These data highlight the importance of frailty screening. In addition, they can serve to guide resource allocation and provide comparative estimates for future research.

**Editor's key points**

• Sprint National Anaesthesia Project-3 (SNAP-3) was designed to describe the clinical impact of frailty, multimorbidity, and delirium, and their management, on outcomes after surgery in older patients in the UK.
• A prospective observational cohort study was conducted during 5 days in March 2022, aiming to recruit all patients aged 60 yr and older undergoing surgery in UK National Health Service hospitals.
• Of 7134 patients from 214 hospitals recruited, 19% were living with frailty and 63% were living with multimorbidity (two or more comorbidities).
• Patients living with frailty or multimorbidity were typically older, were from lower socioeconomic backgrounds, took more medications, and had reduced independence.
• These data from a heterogenous cohort of older surgical patients in the UK highlight the importance of perioperative frailty screening and provide baseline data for future research.

The UK population is ageing, with the proportion of those aged older than 85 yr expected to nearly double to 4.3% by 2045.^1^ The surgical population is ageing even faster; by 2030, one-fifth of people aged 75 yr and older are predicted to undergo surgery each year.^2^ Surgery can alleviate symptoms and extend life but carries higher risks for older patients, including postoperative complications, longer hospital stays, increased mortality, slower recovery, and reduced quality of life.^3^, ^4^, ^5^ Understanding this higher-risk group is essential for planning future perioperative services.

Multimorbidity and frailty are common in older adults. Multimorbidity, the presence of multiple long-term health conditions in an individual, complicates disease recognition, management, and prevention. It also contributes to lower quality of life, functional decline, disability, and increased mortality.^6^, ^7^, ^8^ Frailty, a syndrome of multidomain decline in physiological reserve and function, heightens vulnerability to minor stressors.^9^ Approximately 70% of people living with frailty are also living with multimorbidity.^10^ Frailty is associated with higher rates of postoperative complications, longer hospital stays, more frequent readmissions, increased mortality, poorer quality of life, and reduced independence.^3^^,^^11^, ^12^, ^13^, ^14^, ^15^, ^16^

The estimated prevalence of multimorbidity in the general UK population ranges from 10% to 23%,^17^^,^^18^ whereas estimates of frailty in those aged ≥50 yr range from 8% to 39%.^19^^,^^20^ Despite being common, the interplay between these conditions in the older surgical population is not fully understood. Frailty prevalence in surgical patients varies widely by subgroup. For example, the prevalence is 17% among older patients undergoing elective surgery^21^ and almost double (32%) among those requiring emergency laparotomy.^22^ Multimorbidity is less studied, with one study reporting a prevalence of 74% among nonelective general surgical patients.^23^ To our knowledge, no published study provides an overall prevalence of frailty, multimorbidity, and their interplay within a heterogeneous cohort of older surgical patients across the UK.

The Sprint National Anaesthesia Project-3 (SNAP-3) study aims to describe the clinical impact of frailty, multimorbidity, and delirium, and their management, on outcomes after surgery in patients aged 60 yr and older.^24^ Here, we describe the clinical characteristics and prevalence of frailty and multimorbidity for a heterogeneous cohort of older surgical patients in the UK.

## Methods

This was a planned analysis of data collected as part of SNAP-3, the methodology and regulatory approvals of which have been described.^24^ In summary, all UK hospitals that deliver adult surgical services were invited to participate in a prospective observational cohort study. The study aimed to recruit all patients aged 60 yr or older undergoing a surgical procedure during five consecutive days (Monday to Friday) in March 2022. The study was conducted during the COVID-19 pandemic, between the waves of the Delta, Omicron, and BA.2 Omicron variants. Despite the challenges posed by the severity and duration of the pandemic, SNAP-3 aimed to recruit a representative cohort whilst navigating the impact of the pandemic.

The cohort included those undergoing surgery under general, regional, neuraxial, and local anaesthesia. SNAP-3 included those without capacity to consent to the study. The main exclusion criteria were patients undergoing very minor procedures, including cataract surgery (Supplementary Table S1). Ethical approval was provided by the Wales Research Ethics Service (21/WA/0203) and the Scotland A Research Ethics Committee (302033) in July and September 2021, respectively. Electronically recorded or written informed consent or assent was obtained from all participants, a consultee, or personal legal representative. A parallel study using a protocol adapted to local regulations has been conducted in Australia and will be reported separately.

Local investigators collected demographic data, medical and surgical history, laboratory data, SARS-CoV-2 status, surgical risk scores, socioeconomic data, and frailty assessments. Frailty status was determined by the Clinical Frailty Score (CFS), Reported Edmonton Frailty Score (REFS), and electronic Frailty Index (eFI).^25^, ^26^, ^27^ Multimorbidity was reported as a count of two or more specified comorbidities. The comorbidities included in this list were adapted from the Charlson Comorbidity Index to include other conditions known to be relevant in the perioperative period, such as obstructive sleep apnoea and atrial fibrillation (Supplementary Table S2). Patients were followed up 120 days after their index operation to ascertain quality of life using EuroQol (EQ)-5D-5L and EQ-VAS. We used population-based healthcare administration records (NHS Digital, Digital Health and Care Wales, and NHS National Services Scotland) for further data regarding readmission, discharge, mortality, and comorbidities. Data on specific subgroups, delirium, and outcomes associated with multimorbidity and frailty will be reported separately. Local investigators were resident anaesthetists and research nurses supported by consultant anaesthetists. All local investigators were directed to web-based training in the completion of the CFS or REFS.

The *a priori* power calculation indicated that a sample size of approximately 7200 was needed to estimate the prevalence of frailty (estimated upper bound of 0.25) and incidence of delirium (estimated range of 0.05–0.25) with a one percentage point margin of error. Because of the COVID-19 pandemic, the recruitment window was reduced from 7 to 5 days (excluding weekends), and a decrease in surgical activity was expected. The 5-day recruitment was anticipated to still have sufficient statistical power.

Descriptive summary statistics have been used to report demographic details of the SNAP-3 participants. The cohort aims to be a study of the whole older surgical population, so we will not report statistical comparisons. Data are presented as proportions, mean (standard deviation [sd]), and median (interquartile range [IQR]), as appropriate. Confidence intervals (CIs) of proportions were obtained using bootstrapping. All analyses were conducted in R (version 4.3.1, R Project for Statistical Computing, Vienna, Austria).^28^ Results are reported in accordance with STROBE guidelines (Supplementary Table S3).

## Results

### Participating hospitals

Of the 263 NHS hospitals across the UK invited to participate, 214 participated, recruiting 7821 patients. Of these, 687 patients were withdrawn from the study (Supplementary Fig. S1), leaving data from 7134 participants for analysis.

### Characteristics of the older surgical population in the UK

The mean (sd) age was 72.8 (8.1) yr, with an equal sex distribution, and 76.8% were aged 60–79 yr. The cohort predominantly identified as White (96.1%, 95% CI 95.6–96.5%) and the remainder as Asian, Black, or mixed ethnicities. Approximately one-third were of normal body mass index (BMI) (18.5–24.9 kg m^−2^; 29.9%, 95% CI 28.9–31%), one-third were overweight (BMI 25.0–29.9 kg m^−2^; 35.9%, 95% CI 34.8–37.1%), and one-third were living with obesity class 1 (BMI 30.0–34.9 kg m^−2^; 28.6%, 95% CI 27.5–29.6%). Extreme categories included 2.0% underweight (BMI <18.5 kg m^−2^; 95% CI 1.7–2.3%) and 3.6% class ≥2 obese (BMI ≥35.0 kg m^−2^; 95% CI 3.2–4.0%) (Table 1, Table 2).

**Table 1:** Patient and clinical characteristics of the SNAP-3 cohort and those living with and without frailty defined by CFS. Data are presented as mean (sd) or percentage (95% CI). ADL, activity of daily living; BMI, body mass index; CFS, Clinical Frailty Score; CI, confidence interval; IMD, Index of Multiple Deprivation. Percentages have been rounded, so they may not total 100% exactly. Missing data are omitted from this table but reported in Supplementary Table S11.

Characteristic	Overall cohort		Frail (CFS ≥5)		Not frail (CFS <5)	
	*n*=7134	*n*	*n*=1369	*n*	*n*=5628	*n*
Age (yr)	72.8 (8.1)	7056	77 (9.3)	1368	71.8 (7.5)	5624
60–69	36.6 (35.5–37.8)	2585	25.1 (22.8–27.5)	344	39.4 (38.1–40.7)	2217
70–79	40.2 (39.1–41.4)	2839	30.7 (28.4–33.2)	420	42.5 (41.3–43.8)	2392
80–89	19.9 (18.9–20.8)	1401	32.7 (30.3–35.3)	448	16.7 (15.8–17.7)	940
≥90	3.3 (2.9–3.7)	231	11.4 (9.7–13)	156	1.3 (1–1.6)	75
Sex						
Female	49.1 (47.8–50.2)	3465	57.7 (55.2–60.2)	790	46.9 (45.6–48.2)	2642
Male	50.9 (49.8–52)	3595	42.3 (39.7–45)	579	53.1 (51.8–54.3)	2986
Ethnicity						
Asian	2 (1.7–2.3)	138	2.4 (1.5–3.2)	32	1.9 (1.5–2.2)	104
Black	1.4 (1.1–1.7)	100	1.9 (1.3–2.7)	26	1.3 (1–1.6)	72
Mixed	0.5 (0.4–0.7)	36	0.7 (0.3–1.3)	10	0.5 (0.3–0.7)	26
White	96.1 (95.6–96.5)	6704	95 (93.8–96.1)	1287	96.4 (95.9–96.9)	5361
IMD decile						
1 (least deprived)	6.8 (6.2–7.4)	469	10.8 (9.1–12.4)	144	5.8 (5.2–6.5)	319
2	7.6 (6.9–8.1)	520	10.9 (9.3–12.6)	145	6.7 (6.1–7.4)	369
3	8.2 (7.6–8.9)	566	10.7 (9.1–12.4)	143	7.7 (6.9–8.3)	420
4	8.9 (8.2–9.6)	612	9.5 (7.9–11.1)	127	8.8 (8.1–9.5)	482
5	10 (9.3–10.7)	686	9.2 (7.7–10.9)	123	10.1 (9.3–10.9)	553
6	12.1 (11.3–12.8)	831	11.6 (10–13.3)	155	12.2 (11.4–13.1)	671
7	11.8 (11.1–12.5)	812	10.4 (8.8–12.1)	139	12.1 (11.3–13)	665
8	11.4 (10.6–12.1)	784	9.2 (7.7–10.7)	123	11.9 (11–12.8)	651
9	12.4 (11.7–13.2)	856	10.5 (8.9–12.2)	140	12.9 (12–13.8)	708
10 (most deprived)	10.9 (10.2–11.6)	748	7.1 (5.8–8.5)	95	11.8 (10.9–12.7)	648
BMI						
Underweight	2 (1.7–2.3)	138	5 (3.9–6.1)	67	1.3 (1–1.5)	70
Healthy weight	29.9 (28.9–31)	2096	35.7 (33.1–38.3)	482	28.7 (27.5–29.9)	1603
Overweight	35.9 (34.8–37.1)	2516	26.4 (23.9–28.8)	356	38.2 (36.8–39.5)	2133
Obesity class 1	28.6 (27.5–29.6)	1999	27.8 (25.6–30.2)	375	28.7 (27.6–29.9)	1606
Obesity class ≥2	3.6 (3.2–4)	251	5.2 (3.9–6.4)	70	3.2 (2.8–3.7)	179
Independence with ADLs						
Independent	72.9 (71.9–74)	4991	10.7 (9.2–12.5)	137	87.2 (86.3–88.1)	4813
Assistance with instrumental ADLs	23.8 (22.8–24.9)	1632	73.9 (71.4–76.4)	943	12.3 (11.5–13.2)	680
Assistance with basic ADLs	3.3 (2.8–3.7)	223	15.4 (13.4–17.4)	196	0.5 (0.3–0.7)	26
Multimorbid as ≥2 comorbidities						
Multimorbid	63.1 (62–64.3)	3978	85.9 (84–87.8)	1079	57.4 (56–58.7)	2863
Not multimorbid	36.9 (35.7–38)	2325	14.1 (12.2–16.1)	177	42.6 (41.3–44.1)	2129
CFS						
1	11.5 (10.8–12.3)	808	–	–	14.4 (13.5–15.3)	808
2	19.3 (18.4–20.3)	1351	–	–	24 (22.9–25.1)	1351
3	30.6 (29.5–31.7)	2141	–	–	38 (36.8–39.3)	2141
4	19 (18.1–19.9)	1328	–	–	23.6 (22.5–24.7)	1328
5	9.9 (9.2–10.6)	696	50.8 (48.1–53.5)	696	–	–
6	6 (5.4–6.6)	421	30.8 (28.3–33.1)	421	–	–
7	3.2 (2.8–3.6)	222	16.2 (14.3–18.3)	222	–	–
8	0.4 (0.2–0.5)	25	1.8 (1.2–2.6)	25	–	–
9	0.1 (0–0.1)	5	0.4 (0.1–0.7)	5	–	–

**Table 2:** Patient characteristics of the SNAP-3 cohort and those living with and without multimorbidity as defined by a count of two or more comorbidities. Data are presented as mean (sd) or percentage (95% CI). Percentages have been rounded, so they may not total 100% exactly. Missing data are omitted from this table but reported in Supplementary Table S11. ADL, activity of daily living; BMI, body mass index; CFS, Clinical Frailty Score; IMD, Index of Multiple Deprivation.

Characteristic	Overall cohort		Multimorbid (≥2 comorbidities)		Not multimorbid (<2 comorbidities)	
	*n*=7134	*n*	*n*=3978	*n*	*n*=2325	*n*
Age (yr)	72.8 (8.1)	7056	74.4 (8.1)	3976	70.7 (7.6)	2324
60–69	36.6 (35.5–37.8)	2585	29.2 (27.7–30.6)	1160	46.9 (44.9–49)	1090
70–79	40.2 (39.1–41.4)	2839	41.8 (40.2–43.3)	1662	38.5 (36.5–40.4)	895
80–89	19.9 (18.9–20.8)	1401	24.4 (23.1–25.7)	972	13.1 (11.8–14.5)	305
≥90	3.3 (2.9–3.7)	231	4.6 (4–5.2)	182	1.5 (1–2)	34
Sex						
Female	49.1 (47.8–50.2)	3465	46.3 (44.7–47.8)	1842	54 (51.9–56.1)	1255
Male	50.9 (49.8–52)	3595	53.7 (52–55.3)	2135	46 (44–48.1)	1070
Ethnicity						
Asian	2 (1.7–2.3)	138	1.9 (1.5–2.4)	76	2.1 (1.6–2.7)	49
Black	1.4 (1.1–1.7)	100	1.6 (1.2–2)	64	1.1 (0.7–1.6)	26
Mixed	0.5 (0.4–0.7)	36	0.5 (0.3–0.8)	21	0.4 (0.2–0.7)	9
White	96.1 (95.6–96.5)	6704	95.9 (95.2–96.5)	3775	96.3 (95.6–97)	2207
IMD decile						
1	6.8 (6.2–7.4)	469	8.2 (7.3–9.1)	319	4.9 (4–5.8)	110
2	7.6 (6.9–8.1)	520	8.5 (7.6–9.4)	330	6.1 (5.1–7.1)	138
3	8.2 (7.6–8.9)	566	8.7 (7.8–9.6)	337	7.5 (6.4–8.6)	169
4	8.9 (8.2–9.6)	612	8.8 (7.9–9.7)	343	8.9 (7.7–10.1)	201
5	10 (9.3–10.7)	686	9.8 (8.9–10.8)	381	10.1 (8.8–11.3)	229
6	12.1 (11.3–12.8)	831	12 (11–13.1)	465	13 (11.5–14.4)	293
7	11.8 (11.1–12.5)	812	11.3 (10.3–12.3)	439	12.3 (11–13.7)	277
8	11.4 (10.6–12.1)	784	11 (10–11.9)	425	11.7 (10.4–13)	264
9	12.4 (11.7–13.2)	856	11.9 (10.9–12.9)	461	13.6 (12.2–15)	308
10 (most deprived)	10.9 (10.2–11.6)	748	9.8 (8.8–10.8)	381	12 (10.7–13.4)	271
BMI						
Underweight	2 (1.7–2.3)	138	2.3 (1.8–2.8)	90	1.6 (1.1–2.1)	37
Healthy weight	29.9 (28.9–31)	2096	26.9 (25.5–28.3)	1063	34.3 (32.4–36.2)	790
Overweight	35.9 (34.8–37.1)	2516	34.5 (33.1–36)	1361	38.3 (36.3–40.3)	881
Obesity class 1	28.6 (27.5–29.6)	1999	31.8 (30.3–33.3)	1256	23.8 (22–25.6)	548
Obesity class ≥2	3.6 (3.2–4)	251	4.5 (3.9–5.1)	177	2 (1.5–2.6)	47
Independence with ADLs						
Independent	72.9 (71.9–74)	4991	63 (61.5–64.5)	2418	87.8 (86.3–89.2)	1993
Assistance with instrumental ADLs	23.8 (22.8–24.9)	1632	32.6 (31–34.1)	1250	10.8 (9.5–12.1)	245
Assistance with basic ADLs	3.3 (2.8–3.7)	223	4.5 (3.8–5.1)	171	1.5 (1–1.9)	33
Frailty by CFS ≥5						
Frail	19.6 (18.6–20.5)	1369	27.4 (26–28.8)	1079	7.7 (6.6–8.8)	177
Not frail	80.4 (79.5–81.4)	5628	72.6 (71.2–74)	2863	92.3 (91.2–93.4)	2129
CFS						
1	11.5 (10.8–12.3)	808	5.6 (4.8–6.3)	220	20.6 (19–22.3)	476
2	19.3 (18.4–20.3)	1351	12.5 (11.5–13.5)	493	29.2 (27.3–31)	673
3	30.6 (29.5–31.7)	2141	30.8 (29.4–32.2)	1215	30.2 (28.3–32.1)	697
4	19 (18.1–19.9)	1328	23.7 (22.4–25.1)	935	12.3 (11.1–13.7)	283
5	9.9 (9.2–10.6)	696	13.7 (12.6–14.8)	540	4.1 (3.3–4.9)	94
6	6 (5.4–6.6)	421	8.7 (7.8–9.5)	341	2 (1.5–2.7)	47
7	3.2 (2.8–3.6)	222	4.4 (3.8–5)	172	1.4 (1–1.9)	33
8	0.4 (0.2–0.5)	25	0.6 (0.3–0.8)	22	0.1 (0–0.2)	2
9	0.1 (0–0.1)	5	0.1 (0–0.2)	4	0 (0–0.1)	1

Approximately one-third (2452/7134, 34.4%) of the SNAP-3 cohort was in the three least deprived deciles of deprivation as measured by the Index of Multiple Deprivation (IMD), and one-fifth (1555/7134, 21.8%) was in the three most deprived IMD deciles. Moreover, 22% (95% CI 21.7–23.7%) of the cohort reported having degree-level education, and 22.7% (95% CI 21.7–23.6%) reported having no formal qualifications (Supplementary Table S4).

Seventy-three percent (95% CI 71.9–74.0%) of participants were independent with activities of daily living (ADLs), 23.8% (95% CI 22.8–24.9%) required assistance with instrumental ADLs (e.g. finances, shopping, and organisation), and 3.3% (95% CI 2.8–3.7%) required assistance with personal ADLs (e.g. hygiene, dressing, and feeding). Of the cohort, 47% (95% CI 45.8–48.1%) were taking at least five medications and so would be classed as having polypharmacy.

### Details of perioperative care for the older surgical population

Orthopaedic surgery was the largest surgical specialty with just under one-third of participants (29.8%,95% CI 28.6–30.8%), followed by 19.2% (95% CI 18.3–20.1%) undergoing urological surgery and 12.2% (95% CI 11.4–12.9%) undergoing colorectal surgery (Table 3, Table 4 and Supplementary Table S5). The most common surgical procedures conducted were primary total knee replacement, transurethral resection of bladder tumour, primary total hip replacement, laparoscopic cholecystectomy, hip fracture surgery, and wide local excision of breast tissue (Table 5). General anaesthesia was the most common anaesthetic technique. In 18.9% (95% CI 17.9–20.1%) of general anaesthesia cases, adjunctive regional and/or neuraxial techniques were provided (Table 6).

**Table 3:** Perioperative details of the SNAP-3 cohort and those living with and without frailty defined by CFS. Data are presented as percentage (95% CI). AHP, allied health professional; ASA, American Society of Anesthesiologists; CFS, Clinical Frailty Score; CI, confidence interval; PACU, postanaesthetic care unit. Percentages have been rounded, so they may not total 100% exactly. Missing data are omitted from this table but reported in Supplementary Table S11. Surgical urgency is defined using NCEPOD categorisations.^51^ Further details regarding surgical specialties can be found in the Supplementary information. PACU admission is defined as nurse-led, protocol-driven, level 1.5/2/3 care for up to 24 h after surgery, in addition to the initial period of active patient management required to safely recover airway reflexes and respiratory and cardiovascular stability after a procedure/anaesthetic. Planned admission to PACU or critical care is defined as an admission that is decided upon or booked before the day of surgery in an elective case or the start of the anaesthetic in emergency cases.

Characteristic	Overall cohort		Frail (CFS ≥5)		Not frail (CFS <5)	
	*n*=7134	*n*	*n*=1369	*n*	*n*=5628	*n*
Surgical urgency						
Emergency	2.3 (2–2.6)	161	3.4 (2.6–4.4)	47	2 (1.6–2.3)	112
Urgent	15 (14.2–15.9)	1062	29.5 (27.2–31.9)	404	11.6 (10.8–12.5)	654
Expedited	13 (12.2–13.8)	915	15.9 (14–17.8)	217	12.3 (11.5–13.2)	692
Elective	69.7 (68.6–70.8)	4922	51.2 (48.6–53.8)	701	74.1 (72.9–75.2)	4169
Surgical specialty (10 most common)						
Breast	5.5 (5–6.1)	386	2.2 (1.5–3)	30	6.4 (5.7–7)	353
Colorectal	12.2 (11.4–12.9)	850	8.2 (6.8–9.7)	111	13.2 (12.3–14)	732
Ear, nose, and throat	3.7 (3.3–4.2)	259	1.8 (1.1–2.4)	24	4.2 (3.6–4.7)	231
Gynaecology	4.8 (4.4–5.4)	337	3.4 (2.4–4.4)	46	5.2 (4.6–5.8)	289
Orthopaedics	29.8 (28.6–30.8)	2072	46.5 (43.9–49.3)	630	25.7 (24.6–26.9)	1426
Plastics	3.8 (3.4–4.3)	266	4.9 (3.8–6.1)	66	3.6 (3.1–4.1)	198
Thoracic	4.2 (3.7–4.7)	290	2 (1.3–2.8)	27	4.7 (4.1–5.2)	259
Upper gastrointestinal	5.3 (4.8–5.8)	368	2.6 (1.8–3.5)	35	5.9 (5.3–6.5)	328
Urology	19.2 (18.3–20.1)	1337	15.1 (13.1–17.1)	204	20.2 (19.1–21.2)	1119
Vascular	2.7 (2.3–3)	186	4 (3–5.1)	54	2.3 (1.9–2.7)	129
ASA physical status						
1	7.3 (6.7–8)	513	0.5 (0.1–1)	7	8.9 (8.2–9.7)	499
2	53.2 (52.1–54.4)	3725	20.2 (18.1–22.4)	275	61.2 (59.9–62.5)	3416
3	35.6 (34.4–36.7)	2490	65.8 (63.2–68.1)	894	28.3 (27.1–29.5)	1577
4	3.7 (3.3–4.2)	261	13.5 (11.8–15.3)	183	1.4 (1.1–1.7)	76
5	0.1 (0.1–0.2)	10	–	–	0.2 (0.1–0.3)	10
Day case	34 (32.7–34.8)	2407	18.5 (16.4–20.5)	253	37.8 (36.4–39)	2127
Postoperative destination						
Ward (level 0 or 1 care)	86 (85.1–86.7)	6059	87.6 (85.8–89.3)	1196	85.6 (84.7–86.5)	4808
Unplanned admission to PACU or equivalent (level 1.5 care)	0.5 (0.3–0.7)	35	0.7 (0.3–1.2)	10	0.4 (0.3–0.6)	25
Planned admission to PACU or equivalent (level 1.5 care)	5.9 (5.4–6.5)	418	4.5 (3.4–5.7)	62	6.3 (5.7–6.9)	354
Unplanned admission to PACU or equivalent (level 2/3 care)	0.2 (0.1–0.3)	13	0.5 (0.1–0.9)	7	0.1 (0–0.2)	6
Planned admission to PACU or equivalent (level 2/3 care)	1.4 (1.2–1.7)	101	1.6 (1–2.3)	22	1.4 (1.1–1.7)	78
Unplanned critical care admission (level 2 or 3 care)	0.7 (0.5–0.9)	51	1.1 (0.6–1.7)	15	0.6 (0.4–0.9)	36
Planned critical care admission (level 2 or 3 care)	5 (4.5–5.5)	355	3.8 (2.8–4.9)	52	5.3 (4.7–5.9)	298

**Table 4:** Perioperative details of the SNAP-3 cohort and those living with and without multimorbidity as defined by a count of two or more comorbidities. Data are presented as percentage (95% CI). AHP, allied health professional; ASA, American Society of Anesthesiologists; CFS, Clinical Frailty Score; CI, confidence interval; PACU, postanaesthetic care unit. Percentages have been rounded, so they may not total 100% exactly. Missing data are omitted from this table but reported in Supplementary Table S11. Surgical urgency is defined using NCEPOD categorisations.^51^ Further details regarding surgical specialties can be found in Supplementary information. PACU admission is defined as nurse-led, protocol-driven, level 1.5/2/3 care for up to 24 h after surgery, in addition to the initial period of active patient management required to safely recover airway reflexes and respiratory and cardiovascular stability after a procedure/anaesthetic. Planned admission to PACU or critical care is defined as an admission that is decided upon or booked before the day of surgery in an elective case or the start of the anaesthetic in emergency cases.

Characteristic	Overall cohort		Multimorbid (≥2 comorbidities)		Not multimorbid (<2 comorbidities	
	*n*=7134	*n*	*n*=3978	*n*	*n*=2325	*n*
Surgical urgency						
Emergency	2.3 (2–2.6)	161	2.4 (1.9–2.9)	95	2.5 (1.8–3.1)	57
Urgent	15 (14.2–15.9)	1062	17 (15.9–18.2)	676	13.8 (12.4–15.2)	320
Expedited	13 (12.2–13.8)	915	14 (12.9–15.1)	557	11.1 (9.9–12.4)	259
Elective	69.7 (68.6–70.8)	4922	6.6 (65.2–68)	2650	72.6 (70.8–74.4)	1689
Surgical specialty (10 most common)						
Breast	5.5 (5–6.1)	386	5.2 (4.5–6)	205	6.5 (5.5–7.6)	149
Colorectal	12.2 (11.4–12.9)	850	11.4 (10.4–12.4)	448	14.4 (12.9–15.9)	329
Ear, nose & throat	3.7 (3.3–4.2)	259	2.8 (2.3–3.4)	111	4.5 (3.7–5.4)	103
Gynaecology	4.8 (4.4–5.4)	337	3.6 (3–4.2)	141	6.5 (5.5–7.6)	150
Orthopaedics	29.8 (28.6–30.8)	2072	30.9 (29.5–32.4)	1216	27.1 (25.3–28.9)	621
Plastics	3.8 (3.4–4.3)	266	3.1 (2.5–3.6)	121	3.4 (2.7–4.2)	78
Thoracic	4.2 (3.7–4.7)	290	5.2 (4.5–5.9)	204	3.1 (2.4–3.9)	72
Upper gastrointestinal	5.3 (4.8–5.8)	368	4.6 (4–5.3)	182	7 (6–8.1)	160
Urology	19.2 (18.3–20.1)	1337	21.3 (20–22.6)	838	17.2 (15.7–18.9)	395
Vascular	2.7 (2.3–3)	186	3.5 (2.9–4.1)	137	1.4 (1–1.9)	32
ASA physical status						
1	7.3 (6.7–8)	513	1.1 (0.8–1.4)	42	15.5 (14.1–17.1)	358
2	53.2 (52.1–54.4)	3725	44.2 (42.7–45.8)	1748	67.3 (65.3–69.2)	1550
3	35.6 (34.4–36.7)	2490	49.1 (47.6–50.7)	1941	15.8 (14.4–17.3)	364
4	3.7 (3.3–4.2)	261	5.5 (4.8–6.1)	217	1.2 (0.8–1.6)	27
5	0.1 (0.1–0.2)	10	0.1 (0–0.3)	5	0.2 (0–0.3)	4
Day case	34 (32.7–34.8)	2407	27.4 (26–28.8)	1089	38.2 (36.2–40.3)	888
Postoperative destination						
Ward (level 0 or 1 care)	86 (85.1–86.7)	6059	83.7 (82.5–84.8)	3322	87.7 (86.3–89.1)	2036
Unplanned admission to PACU or equivalent (level 1.5 care)	0.5 (0.3–0.7)	35	0.6 (0.4–0.9)	25	0.4 (0.2–0.7)	9
Planned admission to PACU or equivalent (level 1.5 care)	5.9 (5.4–6.5)	418	6.3 (5.6–7.1)	251	5.7 (4.8–6.7)	132
Unplanned admission to PACU or equivalent (level 2/3 care)	0.2 (0.1–0.3)	13	0.2 (0.1–0.4)	9	0.2 (0–0.3)	4
Planned admission to PACU or equivalent (level 2/3 care)	1.4 (1.2–1.7)	101	2 (1.5–2.4)	78	0.9 (0.5–1.2)	20
Unplanned critical care admission (level 2 or 3 care)	0.7 (0.5–0.9)	51	0.9 (0.6–1.2)	35	0.6 (0.3–0.9)	14
Planned critical care admission (level 2 or 3 care)	5 (4.5–5.5)	355	6.1 (5.4–6.9)	243	4.3 (3.5–5.1)	99

**Table 5:** Most common surgical procedures carried out in the SNAP-3 cohort of older surgical patients. The distribution of the most common 18 surgical procedures carried out in those aged 60 years and over by surgical speciality in the UK. N (%). Transurethral resection of bladder tumour (TURBT); transurethral resection of the prostate (TURP). Seventy percent (95% CI 68.6–70.8%) of surgery was elective, with the remainder unplanned (including the NCEPOD categories of ‘Expedited’, ‘Urgent’ and ‘Emergency’^51^). Thirty-four percent (2407, 95% CI 32.7–34.8%) of procedures were day-case. The mean (SD) Surgical Outcome Risk Tool (SORT) predicted 30-day morbidity was 22.7% (17.8), and SORT predicted 30-day mortality was 1.9% (4.3) (supplementary Table 6).^52^ Details of modes of anaesthesia are displayed in Table 6. Sixty-two percent of participants received general anaesthesia alone (95% CI 61.1–63.4%), 14.7% received general anaesthesia combined with regional, neuraxial or regional and neuraxial anaesthesia (95% CI 12.6–16.1%). Eighty-six percent of participants (95% CI 85.1–86.7%) were discharged to ward-level care, whilst 5.7% (95% CI 5.2–6.3%) went directly to critical care. Additionally, 6.1% (95% CI 5.9–7.0%) were admitted to a post-anaesthetic care unit (PACU) for level 1.5 care, whilst 1.6% (95% CI 1.3–1.9%) to a PACU for level two or three care.^53^.

Surgical specialty	Procedure	n (%)
Orthopaedic trauma	Hip fracture (all)	484 (6.8%)
	Primary open reduction and internal fixation of long bone	155 (2.2%)
Urology	TURBT	369 (5.2%)
	TURP	117 (1.6%)
	Rigid cystoscopy	107 (1.5%)
	Ureteroscopy	81 (1.1%)
	Endoscopic fragmentation of renal calculi	72 (1%)
	Robot assisted prostatectomy	59 (0.8%)
Elective orthopaedics	Total knee replacement	358 (5%)
	Total hip replacement	300 (4.2%)
Abdominal surgery	Laparoscopic cholecystectomy	216 (3%)
	Primary repair of inguinal hernia	144 (2%)
	Laparoscopically assisted right hemicolectomy	71 (1%)
	Laparoscopic inguinal hernia repair	57 (0.8%)
Breast surgery	Wide local excision breast	168 (2.4%)
	Mastectomy	56 (0.8%)
Gynaecology	Hysteroscopy with biopsy or polypectomy	110 (1.5%)
Cardiothoracics	Coronary artery bypass grafting	67 (0.9%)

**Table 6:** Modes of anaesthesia used in the SNAP-3 cohort of older surgical patients. Distribution of modes of anaesthesia given to older surgical patients in the UK, by intended conscious level and with or without regional or neuraxial anaesthesia. Missing data are omitted from this table but reported in Supplementary Table S11. CI, confidence interval; SNAP-3, Sprint National Anaesthesia Project-3.

Intended conscious level	Anaesthetic technique combination	*n*	% (95% CI)
General anaesthesia	General anaesthesia alone	4314	62.2 (61.1–63.4)
	With regional anaesthesia	617	9.0 (8.2–9.6)
	With neuraxial anaesthesia	364	5.3 (4.7–5.8)
	With regional and neuraxial anaesthesia	26	0.4 (0.2–0.5)
Sedation	Sedation alone	105	1.5 (1.2–1.8)
	With regional anaesthesia	132	1.9 (1.6–2.3)
	With neuraxial anaesthesia	360	5.2 (4.7–5.7)
	With regional and neuraxial anaesthesia	92	1.3 (1.1–1.6)
Awake	Awake alone	184	2.7 (2.3–3.1)
	With regional anaesthesia	223	3.2 (2.8–3.7)
	With neuraxial anaesthesia	451	6.5 (5.9–7.1)
	With regional and neuraxial anaesthesia	64	1.0 (0.7–1.2)
Total		6932	

### The prevalence of frailty and multimorbidity in older surgical patients

Of the older surgical cohort, 19.6% (95% CI 18.7–20.5%) were living with frailty according to CFS (≥5), with a similar proportion classified as frail using REFS (19.0%, 95% CI 18.1–19.2%). eFI was only reported in 42 cases, so we have not included this in our analysis. Of the patients, 63.1% (95% CI 62.0–64.3%) were identified as living with multimorbidity, defined by having two or more comorbidities.

### Characteristics of older surgical patients living with frailty and multimorbidity

**Fig 1:**
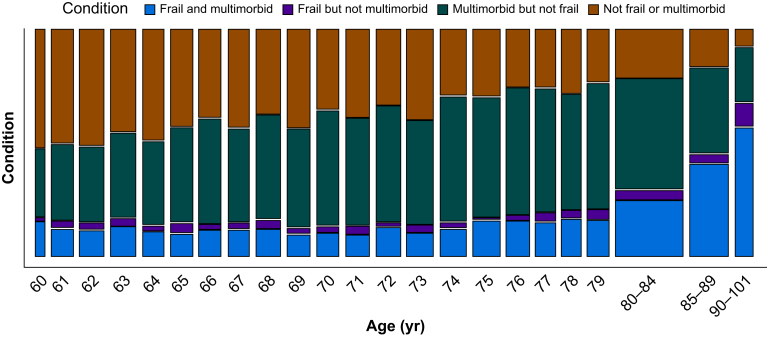
Prevalence of frailty and multimorbidity by age in surgical patients. Mosaic plot illustrating the relationships between age, frailty (defined by Clinical Frailty Score ≥5) and multimorbidity (count of ≥2 comorbidities). The width of the column represents the size of the group. Participants aged 80 yr and older have been grouped owing to small subgroup numbers.

Compared with individuals without frailty, those living with frailty were older and more likely to be female, to have lower education levels, to live in areas with higher levels of socioeconomic deprivation, to have more comorbidities, and to experience polypharmacy and reduced independence with ADLs (Table 1, Fig. 1, Supplementary Fig. S2, and Supplementary Table S4). They were also more often underweight or living with obesity class ≥2 (Table 1). Compared with individuals without multimorbidity, those living with multimorbidity were older and more likely to be male, to experience polypharmacy, to be less independent with ADLs, to have lower levels of education, and to live in areas with higher levels of socioeconomic deprivation (Table 2, Fig. 1, Supplementary Fig. S2, and Supplementary Table S4). They were also more commonly living with obesity class 1 or 2 (Table 2).

### Details of perioperative care for the older surgical population living with frailty and multimorbidity

**Fig 2:**
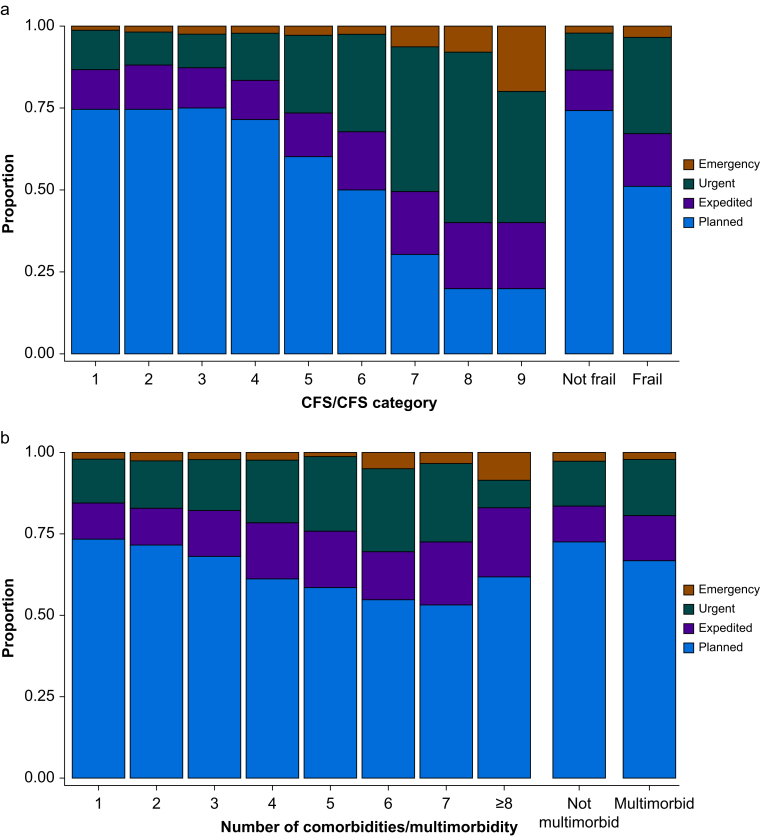
The variation in surgical urgency across frailty and multimorbidity status in patients aged ≥60 yr. Surgical urgency (by NCEPOD definition) varies by frailty and multimorbidity. (a) Surgical urgency by Clinical Frailty Score (CFS), with frailty defined by CFS≥5. (b) Surgical urgency by number of comorbidities, with multimorbidity defined by two or more comorbidities. American Society of Anaesthesiologists' (ASA's) and Surgical Outcome Risk Tool (SORT) scores were generally higher in cohorts with frailty and multimorbidity. The mean SORT-predicted 30-day mortality was more than double for individuals living with frailty than for those without. Among all patients undergoing elective surgery, 8.7% (95% CI 7.9–9.5%) were reviewed in a preoperative anaesthetist-led clinic (either alone or in addition to a nurse-led clinic), 1.3% (95% CI 1.1–1.6%) in a physician-led clinic (in addition to a nurse-led clinic), 1.1% (95% CI 0.8–1.3%) in a geriatrician or MDT-led clinic (in addition to a nurse-led clinic), and 0.2% (95% CI 0.1–0.4%) in a joint anaesthetist and geriatrician-led clinic (in addition to a nurse-led clinic) (Supplementary tables S7 and S8). Elective surgical patients living with frailty or multimorbidity were more frequently seen in anaesthetist-led preassessment clinics than those without these conditions. However, the small numbers in joint anaesthetist–geriatrician-led, geriatrician-led, and physician-led clinics limit meaningful comparisons between groups. The prevalence of day surgery cases in those living with frailty is half that of those without frailty (18.5% *vs* 37.8%). Patients living with multimorbidity were also more likely to be admitted for an inpatient stay after surgery, but the difference in day-case rates between those with and without multimorbidity was smaller (27.4% *vs* 38.2%). Patients living with frailty received more regional and neuraxial anaesthesia, both as their primary anaesthetic and as an adjunct to general anaesthesia, than those without frailty (Supplementary Table S9). Patients living with multimorbidity received the same types of anaesthesia as those without multimorbidity (Supplementary Table S10).

The predominant surgical specialty managing patients living with frailty was orthopaedics (46.5%, 95% CI 43.9–49.3%). Among the entire cohort, ∼1/20 patients underwent hip fracture surgery, with two-thirds of these patients living with frailty. Patients living with frailty were more likely to undergo orthopaedic, vascular, and spinal surgery and less likely to have urology, colorectal, breast, upper gastrointestinal, thoracic, and ear, nose, and throat surgery compared with non-frail patients (Table 3 and Supplementary Fig. S3). The distribution of patients living with multimorbidity across surgical specialties was similar to those without multimorbidity (Table 4 and Supplementary Fig. S3). Those living with frailty were less likely to have elective surgery (51.2%, 95% CI 48.6–53.8%) compared with non-frail individuals (74.1%, 95% CI 72.9–75.2%), a trend not seen in patients living with multimorbidity (Fig. 2). The prevalence of frailty in an individual having unplanned surgery was approximately double that of someone undergoing elective surgery.

### The interplay between frailty and multimorbidity in the older surgical population

Approximately 80% of patients living with frailty (78.8%, 1079/1369) were also living with multimorbidity. In contrast, 27.1% (1079/3978) of those living with multimorbidity were also living with frailty (Supplementary Fig. S4).

### Agreement between frailty measures

SNAP-3 recorded two different measures of frailty: CFS and REFS. When defining frailty, CFS reports frailty as ≥5 and REFS reports frailty as ≥8. There was a high level of agreement between CFS and REFS, with 88.4% (6008/6794) concordance between measures (Table 7 and Supplementary Fig. S5; kappa 0.63 [0.60–0.65]).

**Table 7:** Comparison of frailty assessment by CFS and REFS. A cross-tabulation of frailty measures comparing CFS and REFS is presented. CFS, Clinical Frailty Score; REFS, Reported Edmonton Frailty Score.

		CFS		Total
		Frail (≥5)	Not frail (≤4)	
REFS	Frail (≥8)	906	392	1298
	Not frail (≤7)	394	5102	5496
Totals		1300	5494	6794

## Discussion

We present a comprehensive description of prospectively collected patient-level data in a real-world older UK surgical population, which includes information on perioperative processes of care. This is the first study to describe the older UK surgical population in such detail, with a focus on the prevalence of frailty and multimorbidity. One in five older patients is living with frailty, and two-thirds are living with multimorbidity; these conditions are more prevalent in the older surgical population than in an age-matched community-dwelling population.^10^^,^^18^^,^^19^^,^^29^

We are grateful to our local collaborators for successfully recruiting a large patient cohort representative of the older surgical population in the UK. Our findings will facilitate and guide the development of surgical services for older patients. The older surgical cohort described in SNAP-3 mirrors the characteristics of community-dwelling older adults, as outlined by the UK Census and the Health Survey for England in terms of age, sex, ethnicity, BMI, and socioeconomic deprivation.^30^, ^31^, ^32^ The surgical specialties and urgency levels in our cohort align with recent studies of the wider UK surgical population, such as the 7th National Audit Project (NAP-7) study and NHS England data.^21^^,^^33^, ^34^, ^35^ Consequently, these results are applicable to patients, clinicians, managers, and those involved in service development on a broader scale.

The prevalence of frailty in the older surgical population described in SNAP-3 aligns with other studies focused on specific surgical subgroups and surgical populations across North America and Europe.^3^^,^^21^^,^^36^, ^37^, ^38^, ^39^, ^40^ Notably, the SNAP-3 cohort shows twice the prevalence of frailty compared with community-based studies of older people.^10^^,^^19^^,^^29^

Multimorbidity lacks a universal definition, which causes inconsistency when comparing studies. The reported prevalence of multimorbidity in the SNAP-3 cohort (63%) broadly aligns with other older emergency and elective surgical cohort studies that define multimorbidity as a count of two or more comorbidities.^23^^,^^41^ When multimorbidity is measured using UK administrative data based on ICD-10 codes, its prevalence is lower in part because of the limited sensitivity of this method.^41^ Multimorbidity also shares associations with frailty, including older age, lower socioeconomic status, lower educational attainment, reduced independence, and greater polypharmacy, although the differences between individuals living with and without frailty are more pronounced than those between individuals with and without multimorbidity. The distribution of surgical specialties and perioperative management in the multimorbid subgroup closely mirrors the overall older surgical cohort, partly because of the larger relative size of this subgroup.

The higher prevalence of frailty and multimorbidity in older surgical patients compared with community-dwelling individuals is linked to complex, bidirectional relationships between these conditions and surgical pathologies. For instance, diabetes mellitus and hypertension can lead to complications such peripheral arterial disease requiring surgery, whereas frailty (characterised by reduced mobility, weight loss, chronic inflammation, and immunosuppression) increases the risk of fractures, malignancy, and other conditions that can require surgical intervention. Conversely, surgical problems such as severe osteoarthritis can limit mobility, contributing to frailty and worsening comorbidities. This bidirectional influence can perpetuate poor health outcomes in older adults. It is important to understand that the relationship between frailty and surgical pathology is not straightforward. Surgery can potentially increase, decrease, or have no effect on the severity of frailty that an individual is living with.

Our study highlights the close relationship between frailty and multimorbidity whilst also recognising them as distinct conditions. We found 80% of those living with frailty were also living with multimorbidity, whereas 25% of those living with multimorbidity were also living with frailty. Previous research has shown that frailty can contribute to the development or progression of multiple long-term conditions, thereby leading to multimorbidity. The mechanisms behind this relationship may include comorbidities that contribute to reduced activity levels, which in turn lead to frailty, or the possibility that multimorbidity disrupts fundamental biological processes, such as maintaining the balance between the sympathetic and parasympathetic nervous systems or controlling inflammation, ultimately resulting in frailty.^42^ Multimorbidity can also increase the likelihood of developing frailty, as multiple long-term health conditions often lead to a proinflammatory state, decreased mobility, loss of independence, polypharmacy, and more frequent interactions with healthcare services, all of which contribute to increased vulnerability.^43^ Together, these factors create a situation where an individuals' resilience is diminished, making it more difficult to recover from illness or injury, and heightening their risk of frailty over time.

We suggest that these data have significant implications at both the service and patient levels. Intervention in patients living with frailty is associated with improved outcomes.^44^ Older surgical patients are at a higher risk of frailty, reinforcing the rationale for screening all older surgical patients to ensure appropriate resource allocation for individuals and services.^44^ Our description of the patient and clinical characteristics and perioperative details of those living with frailty can help services focus screening and interventions on patients with the greatest risk. Frailty is most common in emergency inpatient settings and among those undergoing orthopaedic, urological, and colorectal surgeries. Both the prevalence and impact of frailty must be considered when designing perioperative services. Previous studies have shown that the effects of frailty on mortality are more pronounced in lower-risk emergency general procedures, such as appendectomies or cholecystectomies, than in higher-risk procedures, such as bowel resection.^45^ Frailty has also been found to impact outcomes in high-frequency procedures, such as elective total joint arthroplasty.^46^ It is therefore appropriate that perioperative resources are initially more focused in these areas, with rolling dissemination to other areas as resources allow. Similar to many high-income countries, most perioperative resources for older people are directed towards orthogeriatrics, but our data demonstrate the need for expansion of services into other areas where frailty is prevalent, such as emergency surgery.

Clinical guidance recommends that all patients living with frailty undergo a preoperative Comprehensive Geriatric Assessment (CGA) and optimisation.^44^ However, SNAP-3 found that only 8.5% of elective surgical patients attended an anaesthetist-led preoperative clinic where some optimisation might be possible, and only 1% were seen in a geriatrician-led or multidisciplinary team (MDT) clinic, where CGA and optimisation are likely to be performed. Despite the prevalence of frailty, these low review rates suggest that many patients miss key opportunities for holistic appraisal, cognitive assessment, medical optimisation, shared decision-making, and thorough perioperative planning. In the study group's experience, nurse-led preoperative assessment clinics do not generally have the expertise or time to comprehensively assess and optimise a patient with frailty or multimorbidity or to facilitate complex shared decision-making. Those who are living with frailty but are not seen by an appropriate perioperative medicine clinician are more likely to experience increased short-term mortality, complications, and longer length of hospital stay.^47^ An unpublished survey conducted by our study group of 186 hospitals in the UK and Republic of Ireland demonstrated that 28.0% (52/186) of hospitals' nurse-led clinics perform routine frailty screening for all individuals aged 60 yr and older. This demonstrated a gap between clinical evidence and implementation of guidance, likely resulting from under-recognition of frailty, inadequate resources amid rising demand, and lack of awareness of the evidence demonstrating clinical and cost-effectiveness of CGA in the perioperative setting.^48^^,^^49^

A gold standard frailty assessment involves a specialist conducting a CGA, but our use of the CFS and REFS demonstrates that there is a role for the identification of frailty using brief screening tools in older surgical patients. The CFS is the tool of choice to identify frailty and is widely used by nonspecialists as it offers a balance of feasibility and accuracy.^26^^,^^38^ Although our data do not support a clinically relevant difference between CFS and REFS, we suggest that adopting CFS universally has advantages in terms of consistency and interpretation within and between places of care. CFS scoring is not a replacement for CGA but provides a pragmatic screening tool within the perioperative pathway.

SNAP-3 recruited patients from almost all NHS hospitals and is the most comprehensive study of frailty and multimorbidity in UK surgical patients to date. As such, the findings are generalisable to NHS hospitals and immediately relevant to clinicians and policymakers.

SNAP-3 does have limitations, including potentially not recruiting all eligible patients because of investigator availability, time constraints, and non-consenting participants. There could have been selection bias derived from the hospitals that chose not to participate; however, given more than 80% (214/263) of eligible UK hospitals were recruiting centres, this is not anticipated to be a significant issue. Data might not be representative of the emergency surgery patient population because recruitment did not occur over the weekend, a decision made to minimise potential investigator burden. Although the recruitment process was designed to include participants without capacity, it could represent an underestimate of this patient group (Supplementary Table S4). Missing data are an unavoidable challenge in large observational studies; our most frequently missing data points were complete assessments of comorbidities (11.6% missing) and educational attainment (11.0% missing). Aside from these variables, <4% of data were missing for participants (Supplementary Table S11). Although missing data have the potential to introduce bias and reduce the precision of estimates, the low overall proportion of missing information minimises this risk. In addition, because the aim of this paper is primarily descriptive, rather than inferring associations, the impact of missing data on the study's findings is limited.

Conducting this cohort study during the COVID-19 pandemic inevitably influenced the cohort's clinical characteristics. Despite the challenges, the decision to proceed with SNAP-3 was essential because of the uncertainty of the pandemic's duration and the anticipation that the NHS would take considerable time to return to a ‘new normal’. Although the pandemic affected sample size and composition, rates of COVID-19 infection in the cohort were low, and we believe the key findings and clinical implications are valid (Supplementary Table S6).

By collecting comorbidity and demographic data contemporaneously, we address some limitations observed in larger, administratively collected datasets. Our data, gathered from a universal healthcare system with broad national research engagement, are representative of the UK population and likely of other similar populations in comparable healthcare systems. We cannot exclude under-reporting of comorbidity.^50^ However, our data should accurately reflect the population familiar to UK clinicians.

### Conclusions

One in five older surgical patients in the UK are living with frailty, and nearly two-thirds of older patients are living with multimorbidity. These findings highlight the need for perioperative frailty screening and identification to use evidence-based, targeted interventions. Improved recognition of frailty and multimorbidity will enhance patient-centred decision-making and clinical care whilst guiding the strategic focus of surgical services for the increasingly older surgical population.

## Authors' contributions

Initiated the collaborative project, serves as guarantor and grant holder, revised the draft paper, cowrote the analysis plan, and analysed the data: IKM

Obtained ethical and regulatory approvals, implemented the study in the UK, designed the data collection tools, monitored data collection for the study, cowrote the statistical analysis plan, cleaned and analysed the data, and drafted and revised the manuscript: CS

Provided statistical expertise in study design and cowrote the analysis manuscript: PM, HB

Provided expertise in geriatric medicine, designed data collection tools and protocol: JP

Revised the draft manuscript: JP, TP, AS, SN

Designed data collection tools: TP

Coordinated site and UK-wide activity and obtained regulatory approvals: KW

Provided expertise in anaesthetics and perioperative medicine,: AS

Designed data collection tools: AS

Designed protocol: AS

Provided insights from a patient and public perspective and helped to design the patient-facing documents: BE

Finalised data collection: SN

Reviewed the final draft and contributed to revisions of the manuscript: all authors

## Funding

The Frances and Augustus Newman Foundation and the Royal College of Anaesthetists. Neither funder had input into the design, conduct, or analysis of the study.

## Declaration of interest

IKM is the Director of the Centre for Research and Improvement at the Royal College of Anaesthetists (London, UK). The other authors declare no conflict of interest.

## References

[1] Robards James, Online report (2022). Population and Household Projections.

[2] Fowler A.J., Abbott T.E.F., Prowle J., Pearse R.M. (2019). Age of patients undergoing surgery. Br J Surg.

[3] Parmar K.L., Law J., Carter B. (2021). Frailty in older patients undergoing emergency laparotomy: results from the UK Observational Emergency Laparotomy and Frailty (ELF) study. Ann Surg.

[4] Fowler A.J., Wahedally M.A.H., Abbott T.E.F. (2022). Death after surgery among patients with chronic disease: prospective study of routinely collected data in the English NHS. Br J Anaesth.

[5] Hamdi A., Al-Zubeidy B., Obirieze A. (2016). Lower extremity arterial reconstruction in octogenarians and older. Ann Vasc Surg.

[6] World Health Organization (WHO) (2016). https://www.who.int/publications/i/item/9789241511650.

[7] Suls J., Bayliss E.A., Berry J. (2021). Measuring multimorbidity: selecting the right instrument for the purpose and the datasSource. Med Care.

[8] Farmer C., Fenu E., O’Flynn N., Guthrie B. (2016). Clinical assessment and management of multimorbidity: summary of NICE guidance. BMJ.

[9] Clegg A., Young J., Iliffe S., Rikkert M.O., Rockwood K. (2013). Frailty in elderly people. Lancet.

[10] Vetrano D.L., Palmer K., Marengoni A. (2019). Frailty and multimorbidity: a systematic review and meta-analysis. J Gerontol A Biol Sci Med Sci.

[11] Lin H.-S., Watts J.N., Peel N.M., Hubbard R.E. (2016). Frailty and post-operative outcomes in older surgical patients: a systematic review. BMC Geriatr.

[12] Simon H.L., Reif de Paula T., Profeta da Luz M.M., Nemeth S.K., Moug S.J., Keller D.S. (2020). Frailty in older patients undergoing emergency colorectal surgery: USA National Surgical Quality Improvement Program analysis. Br J Surg.

[13] Thillainadesan J., Mudge A.M., Aitken S.J. (2021). The prognostic performance of frailty for delirium and functional decline in vascular surgery patients. J Am Geriatr Soc.

[14] Van de Ree C.L.P., Landers M.J.F., Kruithof N. (2019). Effect of frailty on quality of life in elderly patients after hip fracture: a longitudinal study. BMJ Open.

[15] Panayi A.C., Orkaby A.R., Sakthivel D. (2019). Impact of frailty on outcomes in surgical patients: a systematic review and meta-analysis. Am J Surg.

[16] McAdams-DeMarco M.A., King E.A., Luo X. (2017). Frailty, length of stay, and mortality in kidney transplant recipients: a national registry and prospective cohort study. Ann Surg.

[17] Kingston A., Robinson L., Booth H., Knapp M., Jagger C. (2018). Projections of multi-morbidity in the older population in England to 2035: estimates from the Population Ageing and Care simulation (PACSim) model. Age Ageing.

[18] Barnett K., Mercer S.W., Norbury M., Watt G., Wyke S., Guthrie B. (2012). Epidemiology of multimorbidity and implications for health care, research, and medical education: a cross-sectional study. Lancet.

[19] Sinclair D.R., Maharani A., Chandola T. (2022). Frailty among older adults and its distribution in England. J Frailty Aging.

[20] Walsh B., Fogg C., Harris S. (2023). Frailty transitions and prevalence in an ageing population: longitudinal analysis of primary care data from an open cohort of adults aged 50 and over in England, 2006–2017. Age and Ageing.

[21] Kane A.D., Soar J., Armstrong R.A. (2023). Patient characteristics, anaesthetic workload and techniques in the UK: an analysis from the 7th National Audit Project (NAP7) activity survey. Anaesthesia.

[22] Royal College of Anaesthetists (RCoA) (2023).

[23] Hewitt J., McCormack C., Tay H.S. (2016). Prevalence of multimorbidity and its association with outcomes in older emergency general surgical patients: an observational study. BMJ Open.

[24] Swarbrick C., Poulton T., Martin P., Partridge J., Moppett I.K. (2023). Study protocol for a national observational cohort investigating frailty, delirium and multimorbidity in older surgical patients: the third Sprint National Anaesthesia Project (SNAP 3). BMJ Open.

[25] Frailty Guideline Working Group (2021).

[26] Aucoin S.D., Hao M., Sohi R. (2020). Accuracy and feasibility of clinically applied frailty instruments before surgery: a systematic review and meta-analysis. Anesthesiology.

[27] Clegg A., Bates C., Young J. (2016). Development and validation of an electronic frailty index using routine primary care electronic health record data. Age Ageing.

[28] R Core Team (2023).

[29] Davies K., Maharani A., Chandola T., Todd C., Pendleton N. (2021). The longitudinal relationship between loneliness, social isolation, and frailty in older adults in England: a prospective analysis. Lancet Healthy Longev.

[30] Office for National Statistics (2022). https://www.ons.gov.uk/peoplepopulationandcommunity/culturalidentity/ethnicity/bulletins/ethnicgroupenglandandwales/census2021.

[31] (2020). Populations by Index of Multiple Deprivation (IMD) decile, England and Wales, Census 2021, Office for National Statistics. https://www.ons.gov.uk/peoplepopulationandcommunity/populationandmigration/populationestimates/adhocs/13773populationsbyindexofmultipledeprivationimddecileenglandandwales2020.

[32] NHS England (2019). https://digital.nhs.uk/data-and-information/publications/statistical/health-survey-for-england/2019.

[33] Abbott T.E.F., Fowler A.J., Dobbs T.D., Harrison E.M., Gillies M.A., Pearse R.M. (2017). Frequency of surgical treatment and related hospital procedures in the UK: a national ecological study using hospital episode statistics. Br J Anaesth.

[34] NHS England (2022). Hospital Admitted Patient Care Activity 2022-23: Open Data - Procedures. https://digital.nhs.uk/data-and-information/publications/statistical/hospital-admitted-patient-care-activity/2022-23.

[35] Chalitsios C.V., Luney M.S., Lindsay W.A., Sanders R.D., McKeever T.M., Moppett I. (2024). Risk of mortality following surgery in patients with a previous cardiovascular event. JAMA Surg.

[36] Harrison S., Harvie D.A., Wensley F. (2022). Frailty in the over 65’s undergoing elective surgery (FIT-65)—a three-day study examining the prevalence of frailty in patients presenting for elective surgery. Perioper Med (Lond).

[37] (2023). Perioperative Quality Improvement Programme Report 4, NIAA Health Services Research Centre.

[38] McIsaac D.I., Taljaard M., Bryson G.L. (2020). Frailty as a predictor of death or new disability after surgery: a prospective cohort study. Ann Surg.

[39] Pitter J.G., Zemplényi A., Babarczy B., Németh B., Kaló Z., Vokó Z. (2024). Frailty prevalence in 42 European countries by age and gender: development of the SHARE Frailty Atlas for Europe. GeroScience.

[40] Hewitt J., Long S., Carter B., Bach S., McCarthy K., Clegg A. (2018). The prevalence of frailty and its association with clinical outcomes in general surgery: a systematic review and meta-analysis. Age Ageing.

[41] Fowler A.J., Wahedally M.A.H., Abbott T.E.F., Prowle J.R., Cromwell D.A., Pearse R.M. (2023). Long-term disease interactions amongst surgical patients: a population cohort study. Br J Anaesth.

[42] Fried L.P., Ferrucci L., Darer J., Williamson J.D., Anderson G. (2004). Untangling the concepts of disability, frailty, and comorbidity: implications for improved targeting and care. J Gerontol A Biol Sci Med Sci.

[43] Whitty C.J.M., Holden B. (2023). Chief Medical Officer’s Annual Report 2023 Health in an Ageing Society.

[44] Perioperative Care of People Living with Frailty, Centre for Perioperative Care, Centre for Perioperative Care and British Geriatrics Society, Royal College of Anaesthetists, date 22/09/21, Frailty Guideline Working Group, CPOC BGS Frailty Guideline 2021, URL https://www.cpoc.org.uk/sites/cpoc/files/documents/2021-09/CPOC-BGS-Frailty-Guideline-2021.pdf, [Accessed 18 September 2021].

[45] Castillo-Angeles M., Cooper Z., Jarman M.P., Sturgeon D., Salim A., Havens J.M. (2021). Association of Frailty with morbidity and mortality in emergency general surgery by procedural risk level. JAMA Surg.

[46] McIsaac D.I., Bryson G.L., van Walraven C. (2016). Association of frailty and 1-year postoperative mortality following major elective noncardiac surgery: a population-based cohort study. JAMA Surg.

[47] Subramaniam A., Tiruvoipati R., Lodge M., Moran C., Srikanth V. (2020). Frailty in the older person undergoing elective surgery: a trigger for enhanced multidisciplinary management—a narrative review. ANZ J Surg.

[48] Eamer G., Taheri A., Chen S.S. (2018). Comprehensive geriatric assessment for older people admitted to a surgical service. Cochrane Database Syst Rev.

[49] Partridge J.S.L., Harari D., Martin F. (2017). Randomized clinical trial of comprehensive geriatric assessment and optimization in vascular surgery. Br J Surg.

[50] Shahab R., Lochrie N., Moppett I.K., Dasgupta P., Partridge J.S.L., Dhesi J.K. (2022). A Description of interventions prompted by preoperative comprehensive geriatric assessment and optimization in older elective noncardiac surgical patients. J Am Med Dir Assoc.

[51] National Confidential Enquiry into Patient Outcome and Death (2004). The NCEPOD Classification of Intervention Place Published Abbey House, 74-76 John Street. https://www.ncepod.org.uk/classification.html.

[52] Wong D.J.N., Harris S., Sahni A. (2020). Developing and validating subjective and objective risk-assessment measures for predicting mortality after major surgery: An international prospective cohort study. PLoS Med.

[53] The Faculty of Intensive Care Medicine ENHANCED CARE: Guidance on service development in the hospital setting Critical Futures. https://www.ficm.ac.uk/sites/ficm/files/documents/2021-10/enhanced_care_guidance_final_-_may_2020-.pdf.

