# Supplementary material for: Characteristics of older patients undergoing surgery in the UK: SNAP-3, a snapshot observational study
Source: Br J Anaesth. 2025 Jan 6;134(2):328–40. doi: 10.1016/j.bja.2024.11.024 (PMC11775840; doi:10.1016/j.bja.2024.11.024)
Supplement: Multimedia component 1 [file mmc1.docx]

# The characteristics of older patients undergoing surgery in the UK: a snapshot observational study - SNAP-3

## Supplementary information

### Contents

#### Supplementary tables

- Supplementary table 1: examples of included and excluded procedures for SNAP-3
- Supplementary table 2: list of comorbidities identified in the SNAP-3 study
- Supplementary table 3: STROBE checklist
- Supplementary table 4: additional information regarding the demographics and characteristics of the SNAP-3 cohort, those living with and without frailty (as defined by a CFS ≥5) and those living with and without multimorbidity (as defined by a count of ≥2 comorbidities)
- Supplementary table 5 prevalence of surgical specialties in the SNAP-3 cohort, those living with and without frailty (as defined by a CFS ≥5) and those living with and without multimorbidity (as defined by a count of ≥2 comorbidities)
- Supplementary table 6: additional information regarding the perioperative details of the SNAP-3 cohort, for those living with and without frailty (as defined by CFS≥5) and for those living with and without multimorbidity (as defined by ≥2 comorbidities)
- Supplementary table 7: the preoperative assessment of elective patients in the SNAP-3 cohort by anaesthetist and physician-led services
- Supplementary table 8: the preoperative assessment of elective patients in the SNAP-3 cohort, for those living with and without frailty (as defined by CFS≥5) and for those living with and without multimorbidity (as defined by ≥2 comorbidities)
- Supplementary table 9: anaesthetic techniques used for older surgical patients for the whole cohort and those living with and without frailty (as defined by a CFS ≥5)
- Supplementary table 10: anaesthetic techniques used for older surgical patients for the whole cohort and those living with and without multimorbidity (as defined by a count of ≥2 comorbidities)
- Supplementary table 11: prevalence of missing data in SNAP-3
- Supplementary table 12: SNAP-3 collaborators (in separate file)

#### Supplementary figures

- Supplementary figure 1: participant flow diagram
- Supplementary figure 2: patient demographics and clinical characteristics of those living with and without frailty (as defined by a CFS ≥5) and those living with and without multimorbidity (as defined by a count of ≥2 comorbidities)
- Supplementary figure 3: perioperative details of those living with and without frailty (as defined by a CFS ≥5) and those living with and without multimorbidity (as defined by a count of ≥2 comorbidities)
- Supplementary figure 4: the intersection of frailty (as defined by a CFS ≥5) and multimorbidity (as defined by a count of ≥2 comorbidities) in surgical patients aged ≥60 years
- Supplementary figure 5: bubble plot showing the agreement between Clinical Frailty Scale (frailty defined by a CFS ≥5) and Reported Edmonton Frailty Scale (frailty defined by a REFS ≥8)

### Supplementary table 1: examples of included and excluded procedures for SNAP-3

This list contains examples of included and excluded procedures for SNAP 3. We hope that it will be useful when making decisions regarding whether a participant should be approached for the study. It is not designed to be comprehensive, most surgical procedures are included. We have tried to not include the very minor procedures, but it is challenging to know where to draw the line. We hope this guidance is useful.

**Ophthalmology**

| **Include** | **Exclude** |
| --- | --- |
| Corneal grafts | Any procedure under topical anaesthesia |
| Scleral buckle | LASER (cornea, medical retina) |
| Eyelid reconstruction | Adnexal (eyelid surgery inc. ptosis, blepharoplasty) |
| Keratoplasty | Removal of oil from vitreous body |
| Excision of scalp/skin lesions **if require a split skin graft (SSG) or flap** | Excision of scalp/skin lesions **not requiring a SSG or flap** |
| Vitreoretinal surgery | Superficial eye lid surgery |
| Strabismus surgery | Vitrectomy using pars plana approach |
| Enucleation/eviscerations/orbital decompression | Correction of entropion of lower eyelid |
| Radioactive plaque insertion & removal | Dacryocystorhinostomy |
| Tantalum markers | Cataract surgery |
| Glaucoma surgery | Removal of sutures |
| Anterior orbitotomy | Needling |
| Trabeculectomy | Preserflo microshunt & mitomycin-C |
| Retinal surgery anaesthesia | Cataract surgery (regardless of  anaesthesia mode) |

**General Surgery**

| **Include** | **Exclude** |
| --- | --- |
| Inguinal hernia repair under local anaesthesia +/- sedation | Lymph node biopsy |
| VAC dressing change | Simple dressing change |
| Perianal excision of rectal polyp | Diagnostic and therapeutic endoscopy regardless of anaesthesia mode |
| EUA rectum |  |
| Manual evacuation |  |
| Axillary clearance |  |
| Oesophageal dilation/stenting |  |

**ENT**

| **Include** | **Exclude** |
| --- | --- |
| Excision of larger lesions e.g basal cell carcinoma (BCC)/squamous cell carcinoma (SCC) **e.g. requiring more than primary closure, SSG/flap.**  NB. Mode of anaesthetic here does not influence decision | Excision of smaller BCC/SCC **e.g. no SSG/flap required.**  NB. Mode of anaesthetic here does not influence decision |
| Microlaryngoscopy | Biopsy of tongue |
| Minimally invasive parathyroidectomy | Frenuloplasty |
| Manipulation or examination under anaesthetic nose | Removal salivary tube |
| Cervical lymph node biopsy if GA | Tracheostomy insertion/change |
| Panendoscopy | Grommets |
|  | Anaesthesia for diagnostic procedures |
|  | Tracheo-oesophageal puncture |
|  | Thyroplasties |
|  | Tracheostomy insertion/change |

**Thoracics**

| **Include** | **Exclude** |
| --- | --- |
| Diagnostic bronchoscopy if with other procedure | Endobronchial ultrasound (EBUS) |
| Tracheal stenting | Diagnostic bronchoscopy alone |
| Rigid bronchoscopy | Diagnostic and therapeutic bronchoscopy/pleuroscopy |
| Mediastinoscopy | Chest drain as sole procedure |
| Video assisted thoracoscopic surgery (VATS) |  |
| Endoscopic procedures performed ancillary to surgical procedure ○ Bronchoscopy prior to lung resection |  |

**Cardiac**

| **Include** | **Exclude** |
| --- | --- |
| Transcatheter aortic valve implantation (TAVI) | Ablations |
| Other minimally invasive valve replacement procedures carried out under general anaesthesia | PPM lead extractions |
|  | Angiography, percutaneous coronary intervention (PCI) |
|  | Insertion of permanent pacemaker (PPM) / implantable cardioverter defibrillator (ICD) |
|  | Cardioversion |
|  | Electrophysiology (diagnostic or therapeutic) |
|  | Insertion of intra-aortic balloon pump (IABP) |

**Hands**

| **Include** | **Exclude** |
| --- | --- |
|  | Carpal tunnel decompression under local anaesthetic |
|  | Dupuytren’s palmar fasciectomy |
|  | Trigger finger release |
|  | Excision of hand lesion if small |

**Trauma & Orthopaedics Emergency Department**

| **Include** | **Exclude** |
| --- | --- |
| Ulnar nerve transposition | Aspiration of knee under local anaesthetic |
| Removal of metal work | Cheilectomy |
| Excision of olecranon bursa | Trigger point injections |
| Vertebroplasty | Therapeutic epidural injection |
| Trapeziectomy | Intra-articular joint injections |
| Knee replacement | Dupuytren's fasciectomy |
| Osteotomy of any bone | MUA joint |
| Replacement of hip joint | MUA fracture in ED |
| Replacement of shoulder joint | General anaesthesia/sedation for scanning/ICU management only |
| Small joint fusion | Post-arrest management |
| Insertion K wire | Erector spinae catheters |
| MUA fracture in theatre | Joint injections |
| Surgery for trauma | Joint aspiration |
| MUA fractures/dislocations in theatre |  |
| Joint washout |  |

**Urology**

| **Include** | **Exclude** |
| --- | --- |
| Rigid cystoscopy | Flexible cystoscopy |
| Urethral dilatation | Circumcision under local anaesthetic |
| Transurethral resection of bladder tumour | Standard circumcision under general anaesthetic |
| Transurethral resection of prostate | Transperineal prostate biopsy |
| Hydrocele under general anaesthetic | Flexible ureteroscopy |
| Laser fragmentation of stone | Cystoscopy under local anaesthesia |
| Nephrostomy | Prostate brachytherapy |
| TURP/TURBT |  |
| Rigid diagnostic/surveillance cystoscopy |  |
| Stent change |  |

**Vascular**

| **Include** | **Exclude** |
| --- | --- |
| Fistula ligation and banding | Varicose veins under local anaesthetic |
| Fistula creation |  |
| Endovascular aneurysm repair (EVAR) |  |

**Interventional Radiology**

| **Include** | **Exclude** |
| --- | --- |
| EVAR | CT guided biopsies |
| Angioplasty | IV access/line insertion |
| CT guided drain | Endoscopic retrograde cholangiopancreatography (ERCP) |

**Dental**

| **Include** | **Exclude** |
| --- | --- |
| Extractions |  |

**Gynaecology**

| **Include** | **Exclude** |
| --- | --- |
| **Therapeutic** hysteroscopy | **Diagnostic** hysteroscopy +/- biopsy |
| Laparoscopic hysterectomy | Hysteroscopy and smear |
| Cervical polypectomy |  |

**Neurosurgery**

| **Include** | **Exclude** |
| --- | --- |
| Sympathetic nerve stimulator insertion or removal | SNS battery or lead change |
| Spinal cord stimulator insertion | SNS reprogramming |
|  | SCS trial if purely percutaneous |

### Supplementary table 2: list of comorbidities identified in the SNAP-3 study

| **Comorbidity** | **Description** |
| --- | --- |
| Myocardial infarction | History of MI based on patient history, notes, history of stent |
| Heart failure | Dyspnoea that has responded to heart failure treatment |
| Atrial Fibrillation | Paroxysmal/permanent AF, not if successfully ablated |
| Valvular heart disease | Of any type |
| Hypertension | Even if treated, do not include those with one isolated episode |
| Peripheral vascular disease | Treated and untreated |
| Chronic Obstructive Pulmonary Disease | Probable clinical diagnosis |
| Other chronic lung disease |  |
| Obstructive Sleep Apnoea/obesity hypoventilation syndrome | Symptomatic, not purely positive STOP-BANG |
| Cerebrovascular disease with mild or no residual symptoms | Includes TIA, intracerebral/subarachnoid haemorrhage and stroke diagnosed on CT with no symptoms |
| Hemiplegia or paraplegia | From any cause |
| Dementia |  |
| Mild cognitive impairment |  |
| Anxiety or depression | On treatment |
| Parkinson's disease or parkinsonism |  |
| Diabetes | Not just impaired glucose tolerance or if in remission |
| Moderate or severe renal disease | Acute or chronic, stage 3A+, eGFR< 60 |
| Benign prostatic hypertrophy | Can be self-reported |
| Liver disease | With or without portal hypertension |
| Peptic ulcer disease | Even if treated and not symptomatic |
| Malignancy |  |
| Lymphoma | Of any type, acute or chronic |
| Leukaemia | Of any type, acute or chronic |
| Connective tissue/rheumatological disease | Systemic lupus erythematosus, polymyositis, mixed connective tissue disease, polymyalgia rheumatica, psoriatic arthropathy or rheumatoid arthritis |
| Osteoarthritis | Include self-reported |
| Acquired immunodeficiency syndrome (AIDs) |  |
| Hearing impairment | Uses hearing aids or struggles to manage a conversation at usual volumes of speech |
| Visual impairment | Registered partially sighted |

### Supplementary table 3: STROBE checklist

STROBE Statement—Checklist of items that should be included in reports of ***cohort studies***

|  | Item No | Recommendation | SNAP-3 |
| --- | --- | --- | --- |
| **Title and abstract** | 1 | (*a*) Indicate the study’s design with a commonly used term in the title or the abstract | Page 1 |
|  |  | (*b*) Provide in the abstract an informative and balanced summary of what was done and what was found | Page 2 |
| Introduction | | |  |
| Background/rationale | 2 | Explain the scientific background and rationale for the investigation being reported | Page 4 |
| Objectives | 3 | State specific objectives, including any prespecified hypotheses | Page 4 |
| Methods | | |  |
| Study design | 4 | Present key elements of study design early in the paper | Page 5 |
| Setting | 5 | Describe the setting, locations, and relevant dates, including periods of recruitment, exposure, follow-up, and data collection | Page 5 |
| Participants | 6 | (*a*) Give the eligibility criteria, and the sources and methods of selection of participants. Describe methods of follow-up | Page 5 |
|  |  | (*b*) For matched studies, give matching criteria and number of exposed and unexposed | NA |
| Variables | 7 | Clearly define all outcomes, exposures, predictors, potential confounders, and effect modifiers. Give diagnostic criteria, if applicable | Page 5 (methods paper for details) |
| Data sources/ measurement | 8* | For each variable of interest, give sources of data and details of methods of assessment (measurement). Describe comparability of assessment methods if there is more than one group | Page 5 (methods paper for details) |
| Bias | 9 | Describe any efforts to address potential sources of bias | Page 5 (methods paper for details) |
| Study size | 10 | Explain how the study size was arrived at | Page 5 (methods paper for details) |
| Quantitative variables | 11 | Explain how quantitative variables were handled in the analyses. If applicable, describe which groupings were chosen and why | Page 5 (methods paper for details) |
| Statistical methods | 12 | (*a*) Describe all statistical methods, including those used to control for confounding | Page 5 (methods paper for details) |
|  |  | (*b*) Describe any methods used to examine subgroups and interactions | Page 5 (methods paper for details) |
|  |  | (*c*) Explain how missing data were addressed | NA |
|  |  | (*d*) If applicable, explain how loss to follow-up was addressed | NA |
|  |  | (*e*) Describe any sensitivity analyses | NA |
| Results | | |  |
| Participants | 13* | (a) Report numbers of individuals at each stage of study—eg numbers potentially eligible, examined for eligibility, confirmed eligible, included in the study, completing follow-up, and analysed | Page 7 |
|  |  | (b) Give reasons for non-participation at each stage | Page 7 |
|  |  | (c) Consider use of a flow diagram | Page 7 |
| Descriptive data | 14* | (a) Give characteristics of study participants (eg demographic, clinical, social) and information on exposures and potential confounders | Page 7-24 |
|  |  | (b) Indicate number of participants with missing data for each variable of interest | Page 50 |
|  |  | (c) Summarise follow-up time (eg, average and total amount) | NA |
| Outcome data | 15* | Report numbers of outcome events or summary measures over time | NA |
| Main results | 16 | (*a*) Give unadjusted estimates and, if applicable, confounder-adjusted estimates and their precision (eg, 95% confidence interval). Make clear which confounders were adjusted for and why they were included | Page 7-24 |
|  |  | (*b*) Report category boundaries when continuous variables were categorized | 7-24 |
|  |  | (*c*) If relevant, consider translating estimates of relative risk into absolute risk for a meaningful time period | NA |
| Other analyses | 17 | Report other analyses done—eg analyses of subgroups and interactions, and sensitivity analyses | NA |
| Discussion | | |  |
| Key results | 18 | Summarise key results with reference to study objectives | Page 25 |
| Limitations | 19 | Discuss limitations of the study, taking into account sources of potential bias or imprecision. Discuss both direction and magnitude of any potential bias | Page 27 |
| Interpretation | 20 | Give a cautious overall interpretation of results considering objectives, limitations, multiplicity of analyses, results from similar studies, and other relevant evidence | Page 25-28 |
| Generalisability | 21 | Discuss the generalisability (external validity) of the study results | Page 25 |
| Other information | | |  |
| Funding | 22 | Give the source of funding and the role of the funders for the present study and, if applicable, for the original study on which the present article is based | Page 29 |

*Give information separately for exposed and unexposed groups.

**Note:** An Explanation and Elaboration article discusses each checklist item and gives methodological background and published examples of transparent reporting. The STROBE checklist is best used in conjunction with this article (freely available on the Web sites of PLoS Medicine at http://www.plosmedicine.org/, Annals of Internal Medicine at http://www.annals.org/, and Epidemiology at http://www.epidem.com/). Information on the STROBE Initiative is available at <http://www.strobe-statement.org>.

### Supplementary table 4: additional information regarding the demographics and characteristics of the SNAP-3 cohort, those living with and without frailty (as defined by a CFS ≥5) and those living with and without multimorbidity (as defined by a count of ≥2 comorbidities)

|  | ***Overall cohort*** | | ***Frail (Clinical Frailty Score ≥5)*** | | ***Not frail (Clinical Frailty Score <5)*** | | **Multimorbid (≥2 comorbidities)** | | **Not multimorbid (<2 comorbidities** | |
| --- | --- | --- | --- | --- | --- | --- | --- | --- | --- | --- |
| ***Characteristic*** | ***N = 7134*** | ***n*** | ***N = 1369*** | ***n*** | ***N = 5628*** | ***n*** | **N = 3978** | **n** | **N = 2325** | **n** |
| ***Highest educational level*** |  |  |  |  |  |  |  |  |  |  |
| Degree level | 22.7 (21.7-23.7) % | 1601 | 13.4 (11.5-15.2) % | 183 | 25.1 (24-26.3) % | 1412 | 19.4 (18.2-20.6) % | 772 | 27.1 (25.3-28.9) % | 629 |
| A levels / NVQ 3 | 12.1 (11.3-12.9) % | 855 | 10.2 (8.6-11.8) % | 139 | 12.6 (11.8-13.5) % | 711 | 11.3 (10.4-12.3) % | 451 | 13.3 (11.9-14.6) % | 308 |
| Apprenticeship | 6.6 (6.1-7.2) % | 468 | 6.3 (5-7.5) % | 86 | 6.8 (6.1-7.4) % | 382 | 6.8 (6-7.6) % | 270 | 6.2 (5.3-7.2) % | 145 |
| GCSEs / NVQ 2 | 14.2 (13.4-15.1) % | 1004 | 11.1 (9.5-12.7) % | 152 | 15.1 (14.1-16.1) % | 851 | 13.1 (12.1-14.3) % | 522 | 15.5 (14.1-17) % | 360 |
| O level / NVQ 1 | 11.7 (11-12.4) % | 825 | 12 (10.2-13.7) % | 164 | 11.7 (10.9-12.6) % | 657 | 12.2 (11.1-13.2) % | 483 | 11.2 (10-12.5) % | 261 |
| No formal qualifications | 22.7 (21.7-23.6) % | 1598 | 32.9 (30.5-35.3) % | 450 | 20.3 (19.3-21.5) % | 1144 | 25.8 (24.4-27.1) % | 1025 | 18.2 (16.6-19.7) % | 423 |
| ***Polypharmacy*** |  |  |  |  |  |  |  |  |  |  |
| Polypharmacy (≥5 medications) | 47 (45.8-48.1) % | 3284 | 75.4 (73.1-77.7) % | 1016 | 40.1 (38.9-41.4) % | 2241 | 64.6 (63.1-66.2) % | 2547 | 19.2 (17.6-20.8) % | 443 |
| No polypharmacy (<5 medications) | 53 (51.8-54.2) % | 3709 | 24.6 (22.3-26.7) % | 331 | 59.9 (58.6-61.1) % | 3343 | 35.4 (33.8-37) % | 1395 | 80.8 (79.1-82.3) % | 1860 |
| ***Source of admission*** |  |  |  |  |  |  |  |  |  |  |
| Own home | 95.1 (94.5-95.5) % | 6708 | 86.8 (84.9-88.6) % | 1188 | 97.2 (96.8-97.7) % | 5468 | 94 (93.3-94.7) % | 3736 | 96.9 (96.1-97.5) % | 2251 |
| Sheltered housing | 1.4 (1.1-1.7) % | 100 | 3.7 (2.7-4.7) % | 51 | 0.9 (0.6-1.1) % | 49 | 1.7 (1.3-2.1) % | 68 | 0.8 (0.5-1.2) % | 19 |
| Residential or nursing home | 1.6 (1.3-1.9) % | 111 | 7.1 (5.8-8.5) % | 97 | 0.2 (0.1-0.4) % | 13 | 2.3 (1.9-2.8) % | 91 | 0.5 (0.3-0.8) % | 12 |
| Rehabilitation facility | 0.1 (0-0.2) % | 8 | 0.5 (0.1-0.9) % | 7 | - | - | 0.1 (0-0.2) % | 4 | 0.1 (0-0.3) % | 3 |
| Another secondary care hospital | 0.6 (0.4-0.8) % | 44 | 0.5 (0.1-0.9) % | 7 | 0.6 (0.4-0.8) % | 35 | 0.8 (0.5-1) % | 30 | 0.5 (0.3-0.8) % | 12 |
| ***Capacity to consent to the study*** |  |  |  |  |  |  |  |  |  |  |
| Capacity | 96.6 (96.1-97) % | 6888 | 84.8 (82.9-86.7) % | 1161 | 99.5 (99.3-99.7) % | 5600 | 95 (94.3-95.7) % | 3778 | 99 (98.5-99.4) % | 2301 |
| Consultee | 3.4 (3-3.9) % | 246 | 15.2 (13.4-17.2) % | 208 | 0.5 (0.3-0.7) % | 28 | 5 (4.3-5.7) % | 200 | 1 (0.6-1.5) % | 24 |

Data are reported as percentage (95% CI). ADL, Activities of Daily Living; BMI, Body Mass Index; CFS, Clinical Frailty Score; IMD, Index of Multiple Deprivation. Percentages have been rounded so may not total 100% exactly. Missing data are omitted from this table but reported in supplementary table 11.

Education definitions were defined as the UK census:

- Degree level included those taking under or postgraduate degrees, NVQ Level 4-5, Higher National Certificate, Higher National Diploma, BTEC Higher Level, professional qualifications (e.g. teaching or nursing) or other equivalent higher education qualifications. These are usually taken ≥18 years.
- A levels / NVQ 3 included those with ≥2 A levels/VCEs, ≥4 AS Levels, Higher School Certificate, NVQ Level 3, Advanced GNVQ, City and Guilds Advanced Craft, BTEC National, Scottish Higher National Diploma, Scottish Higher National Certificate, SVQ level 4+) or equivalent. These are usually taken aged 16-19 years old.
- Apprenticeship includes those undertaking supervised training usually in a trade or skill, usually accompanied by a course from an institution of further education. These are usually taken aged 16-19 years old.
- GCSEs / NVQ 2 included those attaining ≥5 O Levels (passes)/GCSEs (grade 1), School Certificate, 1 A Level, 2-3 AS Levels/VCEs, NVQ Level 2, Intermediate GNVQ, City and Guilds Craft, BTEC, Scottish Higher, Scottish Advanced Higher or equivalent qualifications. These are usually taken aged 15-16 years old.
- O level / NVQ 1 includes those attaining O levels/GCSEs (any grade), Foundation Diploma, NVQ level 1, Foundation GNVQ, O grade, Scottish Standard Grade or equivalent qualifications. These are usually taken aged 15-16 years old.

### Supplementary table 5 prevalence of surgical specialties in the SNAP-3 cohort, those living with and without frailty (as defined by a CFS ≥5) and those living with and without multimorbidity (as defined by a count of ≥2 comorbidities)

|  | ***Overall cohort*** | | ***Frail (Clinical Frailty Score ≥5)*** | | ***Not frail (Clinical Frailty Score <5)*** | | ***Multimorbid (≥2 comorbidities)*** | | ***Not multimorbid (<2 comorbidities*** | |
| --- | --- | --- | --- | --- | --- | --- | --- | --- | --- | --- |
| ***Characteristic*** | ***N = 7134*** | ***n*** | ***N = 1369*** | ***n*** | ***N = 5628*** | ***n*** | ***N = 3978*** | ***n*** | ***N = 2325*** | ***n*** |
| *Bariatric* | *0.1 (0.1-0.2) %* | *10* | *0.1 (0-0.2) %* | *1* | *0.2 (0.1-0.3) %* | *9* | *0.1 (0-0.2) %* | *4* | *0.2 (0-0.3) %* | *4* |
| *Breast* | *5.5 (5-6.1) %* | *386* | *2.2 (1.5-3) %* | *30* | *6.4 (5.7-7) %* | *353* | *5.2 (4.5-6) %* | *205* | *6.5 (5.5-7.6) %* | *149* |
| *Cardiology* | *0 (0-0.1) %* | *2* | *-* | *-* | *0 (0-0.1) %* | *2* | *0.1 (0-0.1) %* | *2* | *-* | *-* |
| *Colorectal* | *12.2 (11.4-12.9) %* | *850* | *8.2 (6.8-9.7) %* | *111* | *13.2 (12.3-14) %* | *732* | *11.4 (10.4-12.4) %* | *448* | *14.4 (12.9-15.9) %* | *329* |
| *Endocrine* | *0.9 (0.7-1.1) %* | *61* | *0.4 (0.1-0.7) %* | *5* | *1 (0.7-1.2) %* | *55* | *0.7 (0.4-0.9) %* | *26* | *1.2 (0.7-1.7) %* | *27* |
| *Endoscopic* | *0.4 (0.2-0.5) %* | *25* | *0.6 (0.2-1) %* | *8* | *0.3 (0.2-0.5) %* | *17* | *0.4 (0.2-0.6) %* | *14* | *0.4 (0.2-0.7) %* | *9* |
| *Ear, nose & throat* | *3.7 (3.3-4.2) %* | *259* | *1.8 (1.1-2.4) %* | *24* | *4.2 (3.6-4.7) %* | *231* | *2.8 (2.3-3.4) %* | *111* | *4.5 (3.7-5.4) %* | *103* |
| *Gynaecology* | *4.8 (4.4-5.4) %* | *337* | *3.4 (2.4-4.4) %* | *46* | *5.2 (4.6-5.8) %* | *289* | *3.6 (3-4.2) %* | *141* | *6.5 (5.5-7.6) %* | *150* |
| *Hepato-pancreato-biliary* | *0.6 (0.4-0.8) %* | *43* | *0.1 (0-0.4) %* | *2* | *0.7 (0.5-1) %* | *41* | *0.6 (0.4-0.8) %* | *23* | *0.8 (0.4-1.2) %* | *18* |
| *Interventional radiology* | *1.1 (0.9-1.4) %* | *78* | *1.6 (1-2.4) %* | *22* | *1 (0.8-1.3) %* | *56* | *1.5 (1.1-1.9) %* | *58* | *0.8 (0.4-1.1) %* | *18* |
| *Maxillo-facial and dental* | *1.3 (1.1-1.6) %* | *93* | *0.8 (0.4-1.3) %* | *11* | *1.5 (1.2-1.8) %* | *82* | *1.2 (0.9-1.5) %* | *47* | *1.5 (1-2.1) %* | *35* |
| *Neurosurgery* | *0.9 (0.7-1.2) %* | *66* | *1.3 (0.7-2) %* | *18* | *0.9 (0.6-1.1) %* | *48* | *0.8 (0.6-1.1) %* | *32* | *1.2 (0.8-1.7) %* | *28* |
| *Ophthalmic* | *1.2 (0.9-1.5) %* | *83* | *1 (0.5-1.6) %* | *14* | *1.2 (0.9-1.5) %* | *68* | *0.7 (0.5-1) %* | *29* | *0.6 (0.3-0.9) %* | *13* |
| *Orthopaedics* | *29.8 (28.6-30.8) %* | *2072* | *46.5 (43.9-49.3) %* | *630* | *25.7 (24.6-26.9) %* | *1426* | *30.9 (29.5-32.4) %* | *1216* | *27.1 (25.3-28.9) %* | *621* |
| *Plastics* | *3.8 (3.4-4.3) %* | *266* | *4.9 (3.8-6.1) %* | *66* | *3.6 (3.1-4.1) %* | *198* | *3.1 (2.5-3.6) %* | *121* | *3.4 (2.7-4.2) %* | *78* |
| *Spine* | *2.1 (1.7-2.4) %* | *143* | *3.3 (2.4-4.4) %* | *45* | *1.7 (1.4-2.1) %* | *97* | *2.2 (1.8-2.7) %* | *88* | *2.1 (1.6-2.7) %* | *48* |
| *Thoracic* | *4.2 (3.7-4.7) %* | *290* | *2 (1.3-2.8) %* | *27* | *4.7 (4.1-5.2) %* | *259* | *5.2 (4.5-5.9) %* | *204* | *3.1 (2.4-3.9) %* | *72* |
| *Transplant* | *0 (0-0.1) %* | *3* | *0.1 (0-0.2) %* | *1* | *0 (0-0.1) %* | *2* | *0.1 (0-0.2) %* | *3* | *-* | *-* |
| *Upper gastrointestinal* | *5.3 (4.8-5.8) %* | *368* | *2.6 (1.8-3.5) %* | *35* | *5.9 (5.3-6.5) %* | *328* | *4.6 (4-5.3) %* | *182* | *7 (6-8.1) %* | *160* |
| *Urology* | *19.2 (18.3-20.1) %* | *1337* | *15.1 (13.1-17.1) %* | *204* | *20.2 (19.1-21.2) %* | *1119* | *21.3 (20-22.6) %* | *838* | *17.2 (15.7-18.9) %* | *395* |
| *Vascular* | *2.7 (2.3-3) %* | *186* | *4 (3-5.1) %* | *54* | *2.3 (1.9-2.7) %* | *129* | *3.5 (2.9-4.1) %* | *137* | *1.4 (1-1.9) %* | *32* |

Data are reported as percentage (95% CI). Percentages have been rounded so may not total 100% exactly. Missing data are omitted from this table but reported in supplementary table 11.

### Supplementary table 6: additional information regarding the perioperative details of the SNAP-3 cohort, for those living with and without frailty (as defined by CFS≥5) and for those living with and without multimorbidity (as defined by ≥2 comorbidities)

|  | ***Overall cohort*** | | ***Frail (Clinical Frailty Score ≥5)*** | | ***Not frail (Clinical Frailty Score <5)*** | | ***Multimorbid (≥2 comorbidities)*** | | ***Not multimorbid (<2 comorbidities*** | |
| --- | --- | --- | --- | --- | --- | --- | --- | --- | --- | --- |
| ***Characteristic*** | ***N = 7134*** | ***n*** | ***N = 1369*** | ***n*** | ***N = 5628*** | ***n*** | ***N = 3978*** | ***n*** | ***N = 2325*** | ***n*** |
| ***SORT*** |  |  |  |  |  |  |  |  |  |  |
| *30-day predicted morbidity %* | *22.7 (17.8)* | *6960* | *27.7 (16.2)* | *1353* | *21.6 (18)* | *5544* | *26.1 (17.9)* | *3929* | *18.8 (16)* | *2290* |
| *30-day predicted mortality %* | *1.9 (4.3)* | *7046* | *4.3 (6.1)* | *1367* | *1.3 (3.2)* | *5615* | *2.5 (5.1)* | *3971* | *1 (2.5)* | *2322* |
| ***Urinary catheter*** |  |  |  |  |  |  |  |  |  |  |
| *No catheter* | *63 (62-64.2) %* | *4443* | *56.2 (53.5-58.8) %* | *768* | *64.7 (63.5-66) %* | *3637* | *58.8 (57.3-60.3) %* | *2335* | *64.9 (62.9-66.9) %* | *1507* |
| *Long-term/pre-admission catheter* | *2.1 (1.8-2.5) %* | *149* | *5.2 (4.1-6.4) %* | *71* | *1.4 (1.1-1.7) %* | *77* | *2.6 (2.1-3.1) %* | *102* | *1.7 (1.2-2.2) %* | *40* |
| *Electively catheterised pre/intra-op* | *30.9 (29.8-31.9) %* | *2176* | *34 (31.4-36.5) %* | *465* | *30.1 (28.8-31.2) %* | *1689* | *34.8 (33.3-36.2) %* | *1380* | *28.9 (27.1-30.8) %* | *671* |
| *Catheterised post-op* | *2 (1.7-2.3) %* | *140* | *2.3 (1.6-3.2) %* | *32* | *1.9 (1.5-2.3) %* | *108* | *2.2 (1.8-2.7) %* | *89* | *1.9 (1.3-2.4) %* | *43* |
| ***Laboratory data*** |  |  |  |  |  |  |  |  |  |  |
| *Haemoglobin (g L^-1^)* | *132 (17.8)* | *6392* | *123 (20.2)* | *1295* | *134.4 (16.5)* | *5042* | *130.1 (18.8)* | *3901* | *135.6 (15.8)* | *2267* |
| *WCC (g L^-1^)* | *8.2 (4.2)* | *6392* | *9.1 (3.9)* | *1295* | *7.9 (4.1)* | *5042* | *8.4 (4.5)* | *3901* | *7.8 (3.2)* | *2267* |
| *Neutrophil count (x10^9 L^-1^)* | *5.5 (3)* | *6353* | *6.7 (3.5)* | *1287* | *5.2 (2.8)* | *5011* | *5.7 (3.1)* | *3873* | *5.2 (2.8)* | *2257* |
| *Sodium (mmol L^-1^)* | *139 (137-141)* | *6425* | *139 (136-141)* | *1292* | *139 (137-141)* | *5077* | *139 (137-141)* | *3975* | *140 (138-141)* | *2323* |
| *Potassium (mmol L^-1^)* | *4.4 (0.4)* | *6395* | *4.4 (0.5)* | *1287* | *4.4 (0.4)* | *5052* | *4.4 (0.4)* | *3961* | *4.3 (0.4)* | *2307* |
| *Creatinine (micromol L^-1^)* | *77 (64-94)* | *6416* | *78 (62-100)* | *1289* | *77 (65-93)* | *5071* | *80 (66-99)* | *3969* | *73 (62-87)* | *2321* |
| *eGFR (ml/min)* | *75 (61-88)* | *6363* | *68 (53-86)* | *1256* | *76 (61-88)* | *4992* | *71 (57-86)* | *3978* | *80 (65-90)* | *2325* |
| ***COVID-19*** |  |  |  |  |  |  |  |  |  |  |
| *Tested negative or not tested and treated as negative* | *98.5 (98.2-98.8) %* | *6171* | *96.9 (96-97.9) %* | *1224* | *98.9 (98.6-99.2) %* | *4894* | *98.2 (97.8-98.6) %* | *3830* | *99.1 (98.7-99.5) %* | *2231* |
| *Tested positive or not tested and treated as positive* | *1.5 (1.2-1.8) %* | *92* | *3.1 (2.1-4.1) %* | *39* | *1.1 (0.8-1.4) %* | *52* | *1.8 (1.4-2.2) %* | *70* | *0.9 (0.5-1.3) %* | *20* |

Data are reported as percentage (95% CI). ADL, Activities of Daily Living; BMI, Body Mass Index; CFS, Clinical Frailty Score; IMD, Index of Multiple Deprivation. Percentages have been rounded so may not total 100% exactly. Where percentages do not total 100 there are missing data. Missing data are omitted from this table but reported in supplementary table 11. Surgical urgency is defined using NCEPOD categorisations. ^51^ AHP, Allied Health Professional; ASA, American Society of Anesthesiology; eGFR, estimated Glomerular Filtration Rate; PACU, Post-Anaesthetic Care Unit; SORT – Surgical Outcome Risk Tool.^52^

### Supplementary table 7: the preoperative assessment of elective patients in the SNAP-3 cohort by anaesthetist and physician-led services

|  |  | **Preoperative medical clinic** | | |  |
| --- | --- | --- | --- | --- | --- |
|  |  | ***No medical clinic review*** | ***Physician led clinic*** | ***Geriatrician led or MDT clinic*** | **Totals** |
| **Preoperative anaesthetic clinic** | ***A*naesthetic assessment on day of surgery only** | 1196 (24.4) | 59 (1.2) | 13 (0.3) | 1268 (25.9) |
|  | ***Nurse led clinic,*** *no anaesthetist led clinic* | 2469 (50.4) | 38 (0.8) | 12 (0.2) | 2519 (51.5) |
|  | ***Anaesthetist led clinic,*** *either alone or in addition to nurse led clinic* | 814 (16.6) | 49 (1.0) | 29 (0.6) | 892 (18.2) |
|  | ***Unknown*** | 102 (2.1) | 59 (1.2) | 55 (1.1) | 216 (4.4) |
|  | **Totals** | 4581 (93.6) | 205 (4.2) | 109 (2.2) |  |

The number of elective participants reviewed in preoperative assessment clinics or on the day of surgery. Total number of elective participants is 4922, preoperative assessment clinic information available in 4895 elective participants. N (%)

### Supplementary table 8: the preoperative assessment of elective patients in the SNAP-3 cohort, for those living with and without frailty (as defined by CFS≥5) and for those living with and without multimorbidity (as defined by ≥2 comorbidities)

|  | ***Overall cohort*** | | ***Frail (Clinical Frailty Score ≥5)*** | | ***Not frail (Clinical Frailty Score <5)*** | | **Multimorbid (≥2 comorbidities)** | | **Not multimorbid (<2 comorbidities** | |
| --- | --- | --- | --- | --- | --- | --- | --- | --- | --- | --- |
| ***Preoperative assessment*** | ***N = 7134*** | ***n*** | ***N = 1369*** | ***n*** | ***N = 5628*** | ***n*** | **N = 3978** | **n** | **N = 2325** | **n** |
| Anaesthetist and geriatrician led clinics (in addition to a nurse-led clinic) | 0.2 (0.1-0.4) % | 11 | 0.4 (0-1) % | 3 | 0.2 (0-0.3) % | 7 | 0.3 (0.1-0.5) % | 7 | 0.2 (0-0.4) % | 3 |
| Anaesthetist led clinic (either alone or in addition to a nurse-led clinic) | 8.7 (7.9-9.5) % | 418 | 12 (9.7-14.5) % | 82 | 8.2 (7.4-9) % | 335 | 10.3 (9.2-11.5) % | 267 | 6.4 (5.2-7.6) % | 106 |
| Geriatrician led or MDT clinic (in addition to a nurse-led clinic) | 1.1 (0.8-1.3) % | 51 | 0.7 (0.1-1.5) % | 5 | 1 (0.7-1.3) % | 42 | 1.3 (0.8-1.7) % | 33 | 0.5 (0.2-0.9) % | 9 |
| Physician (non-geriatrician) led clinic (in addition to a nurse-led clinic) | 1.3 (1-1.6) % | 63 | 1.5 (0.6-2.5) % | 10 | 1.2 (0.9-1.5) % | 49 | 1.2 (0.8-1.6) % | 31 | 1.6 (1-2.3) % | 27 |
| Nurse or AHP led clinic | 44.9 (43.5-46.3) % | 2165 | 44.7 (41-48.3) % | 305 | 45 (43.5-46.5) % | 1841 | 47 (45.1-48.9) % | 1220 | 43 (40.5-45.4) % | 714 |
| On the day assessment | 43.8 (42.4-45.2) % | 2112 | 40.7 (37-44.2) % | 278 | 44.4 (42.8-45.9) % | 1815 | 40 (38.2-41.9) % | 1039 | 48.3 (45.8-50.7) % | 801 |

Data are reported as proportion (95% CI). AHP, Allied Healthcare Professional, MDT, Multidisciplinary Team. Percentages have been rounded so may not total 100% exactly. Where percentages do not total 100 there are missing data. Missing data are omitted from this table but reported in supplementary table 11.

### Supplementary table 9: anaesthetic techniques used for older surgical patients for the whole cohort and those living with and without frailty (as defined by a CFS ≥5)

| ***Anaesthetic technique*** | ***Awake*** | | ***General*** | | ***Sedation*** | |
| --- | --- | --- | --- | --- | --- | --- |
|  | ***Frail*** | ***Not frail*** | ***Frail*** | ***Not frail*** | ***Frail*** | ***Not frail*** |
| *Neuraxial* | *147 (51.6)* | *302 (48.0)* | *65 (7.4)* | *297 (6.8)* | *91 (47.2)* | *265 (54.2)* |
| *Neuraxial and regional* | *20 (7.0)* | *44 (7.0)* | *3 (0.3)* | *23 (0.5)* | *36 (18.7)* | *56 (11.5)* |
| *Regional* | *71 (24.9)* | *148 (23.5)* | *166 (19.0)* | *448 (10.2)* | *39 (20.2)* | *91 (18.6)* |
| *No additional technique other than local* | *47 (16.5)* | *135 (21.5)* | *639 (73.2)* | *3632 (82.5)* | *27 (14.0)* | *77 (15.7)* |

Data are reported as a cross tabulation with count and column percentage of the subgroups described, n (%).

### Supplementary table 10: anaesthetic techniques used for older surgical patients for the whole cohort and those living with and without multimorbidity (as defined by a count of ≥2 comorbidities)

| ***Anaesthetic technique*** | ***Awake*** | | ***General*** | | ***Sedation*** | |
| --- | --- | --- | --- | --- | --- | --- |
|  | ***Multimorbid*** | ***Not multimorbid*** | ***Multimorbid*** | ***Not multimorbid*** | ***Multimorbid*** | ***Not multimorbid*** |
| *Neuraxial* | 316 (55.1) | 106 (54.6) | 228 (7.8) | 121 (6.4) | 226 (53.3) | 103 (52.0) |
| *Neuraxial and regional* | 53 (9.2) | 9 (4.6) | 15 (0.5) | 9 (0.5) | 66 (15.6) | 22 (11.1) |
| *Regional* | 120 (20.9) | 59 (30.4) | 359 (12.3) | 198 (10.5) | 79 (18.6) | 38 (19.2) |
| *No additional technique other than local* | 85 (14.8) | 20 (10.3) | 2315 (79.4) | 1551 (82.5) | 53 (12.5) | 35 (17.7) |

Data are reported as a cross tabulation with count and column percentage of the subgroups described, n (%).

### Supplementary table 11: prevalence of missing data in SNAP-3

| **Variable of interest** | **Missing data** |
| --- | --- |
| Age | 84 |
| Sex assigned at birth | 78 |
| Ethnicity | 122 |
| BMI | 127 |
| IMD deciles | 250 |
| Education | 783 |
| ADLs | 288 |
| Polypharmacy | 141 |
| Surgical specialty | 170 |
| Surgical procedure | 165 |
| Urgency | 74 |
| Day case | 0 |
| SORT morbidity | 174 |
| SORT mortality | 88 |
| Preoperative clinic | 162 |
| Anaesthetic technique | 202 |
| Postoperative level of care | 86 |
| CFS | 137 |
| REFS | 304 |
| Number of comorbidities | 831 |
| ASA | 135 |

Number of participants with missing data for each variable of interest.

Supplementary figure 1: participant flow diagram


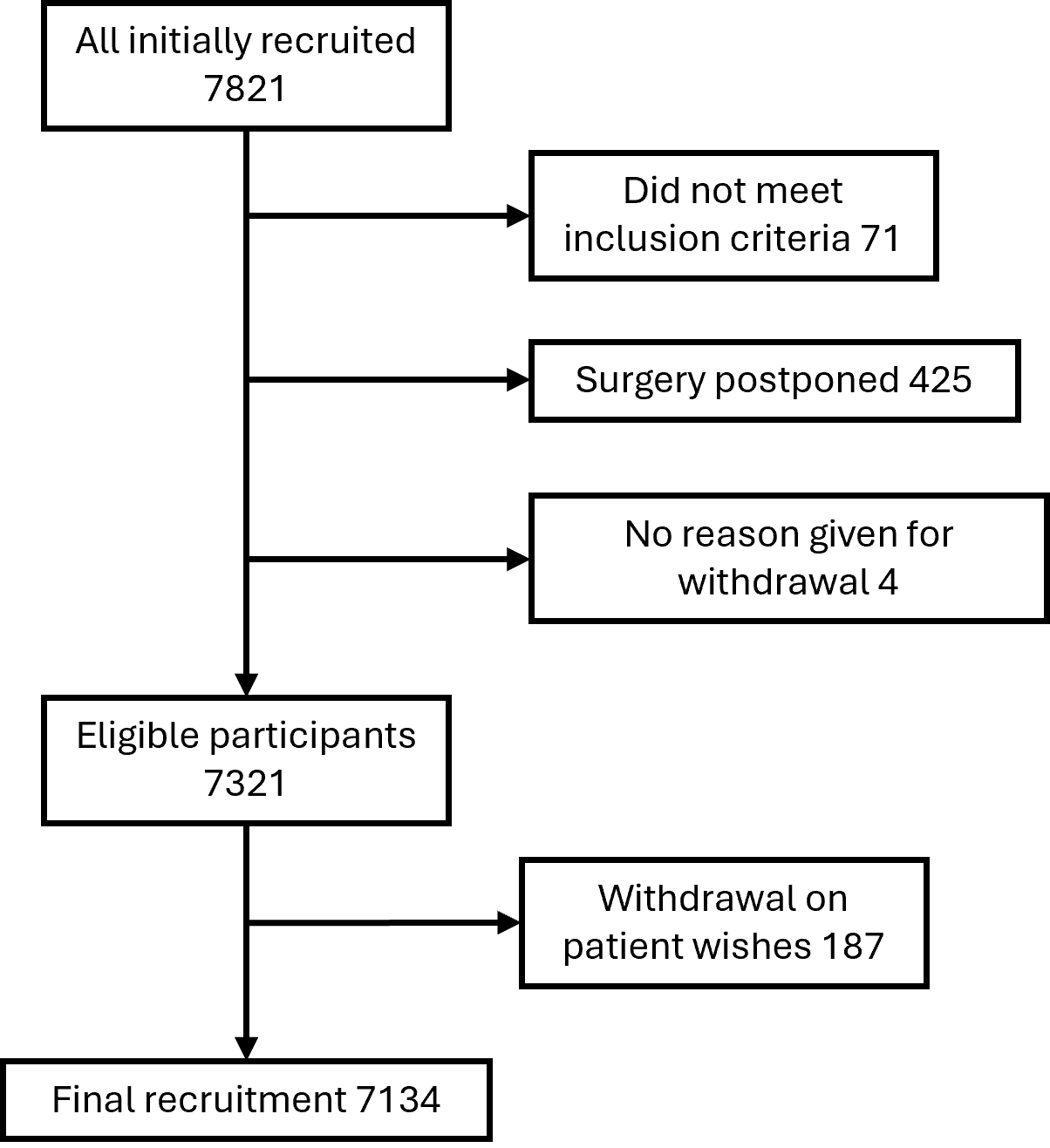


### Supplementary figure 2: patient demographics and clinical characteristics of those living with and without frailty (as defined by a CFS ≥5) and those living with and without multimorbidity (as defined by a count of ≥2 comorbidities)

2a.


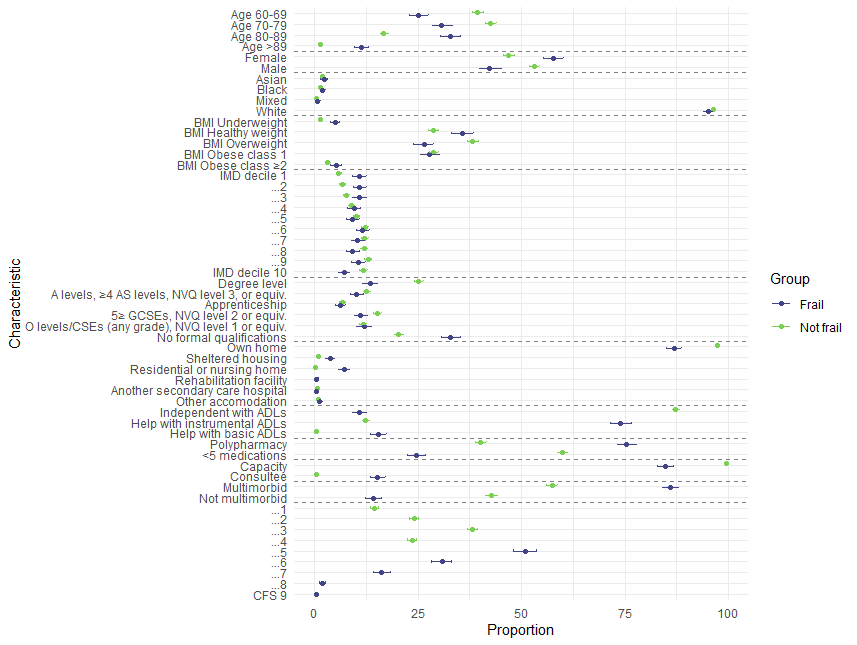


2b.


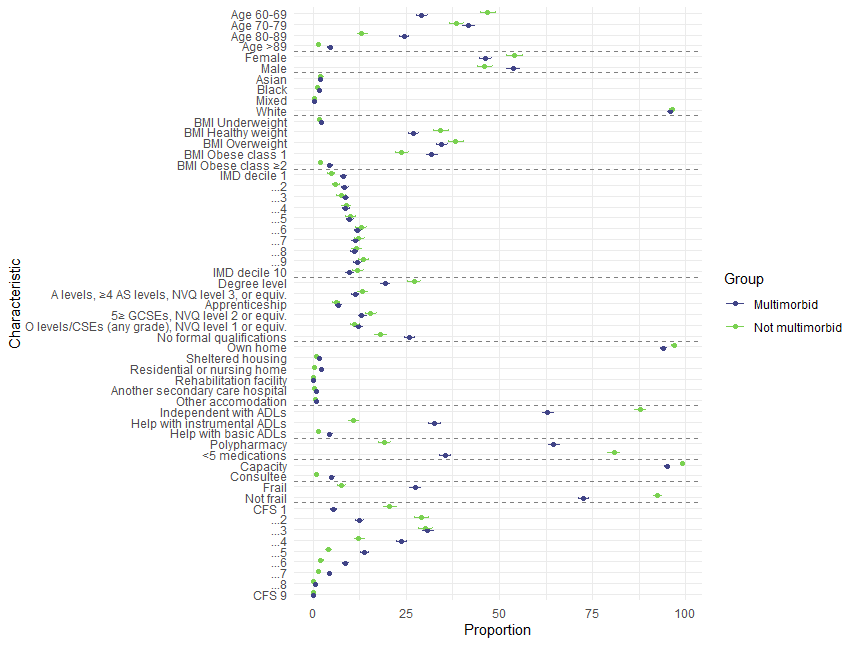


The proportion of patients with and without frailty (panel 2a), and with and without multimorbidity (panel 2b), by demographic and clinical characteristics. Activities of daily living (ADLs), Index of Multiple Deprivation (IMD) decile, body mass index (BMI), National Vocational Qualification (NVQ), General Certificate of Secondary Education (GCSE), and sex assigned at birth. The central dot represents the proportion of patients within each group. The bar extending from the dot shows the 95% confidence interval around this proportion.

### Supplementary figure 3: perioperative details of those living with and without frailty (as defined by a CFS ≥5) and those living with and without multimorbidity (as defined by a count of ≥2 comorbidities)

3a.


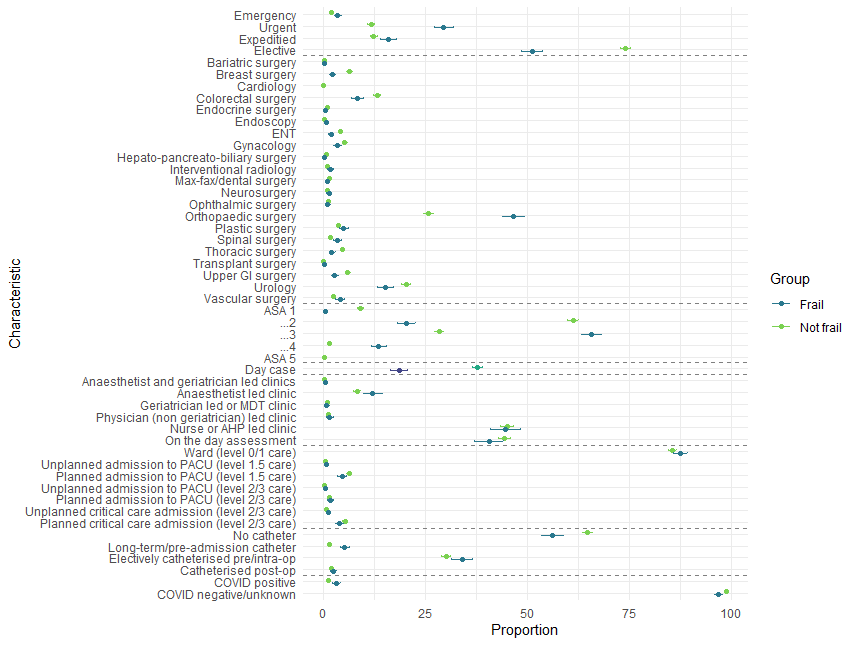


3b.


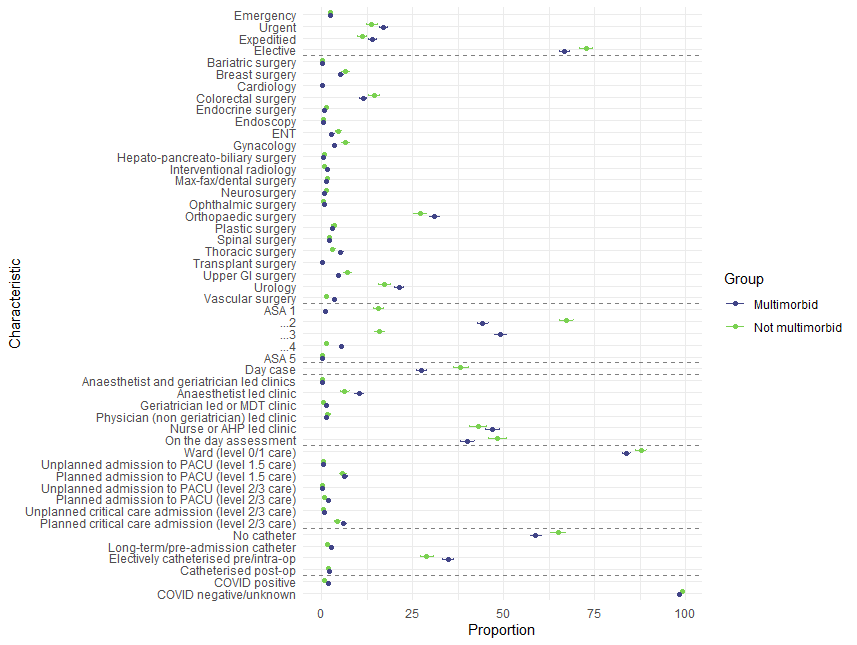


The proportion of patients with and without frailty (panel 2a), and with and without multimorbidity (panel 2b), by perioperative details. Ear nose and throat (ENT), gastrointestinal (GI), American Society of Anesthesiologists physical status (ASA), Alledi Health Professionals (AHP), Post-anaesthetic care unit (PACU). The central dot represents the proportion of patients within each group. The bar extending from the dot shows the 95% confidence interval around this proportion.

### Supplementary figure 4: the intersection of frailty (as defined by a CFS ≥5) and multimorbidity (as defined by a count of ≥2 comorbidities) in surgical patients aged ≥60 years


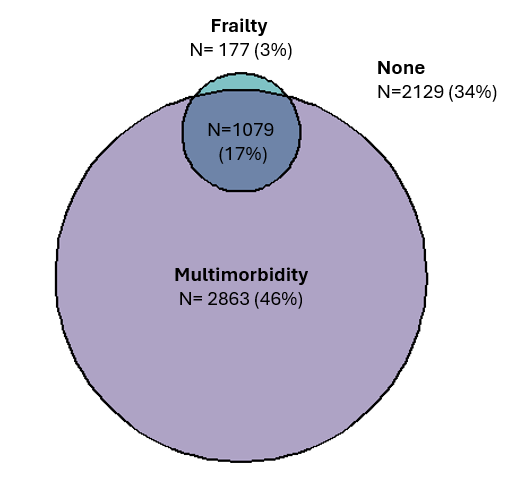


A diagram describing the intersection of frailty and multimorbidity for those who had both the Clinical Frailty Scale and their multimorbidity count completed (N=6248).

### Supplementary figure 5: bubble plot showing the agreement between Clinical Frailty Scale (frailty defined by a CFS ≥5) and Reported Edmonton Frailty Scale (frailty defined by a REFS ≥8)


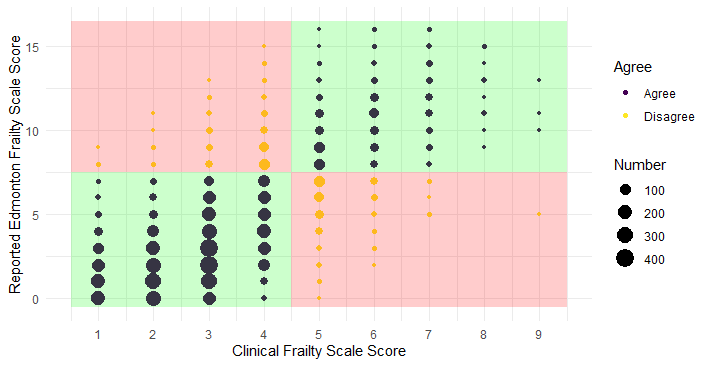


The number of patients (as illustrated by the area of bubble) for those whose Clinical Frailty Scale (CFS) and Reported Edmonton Frailty Scale (REFS) agree (green) and disagree (red).
