# Supplementary material for: Characteristics of older patients undergoing surgery in the UK: SNAP-3, a snapshot observational study
Source: Br J Anaesth. 2025 Jan 6;134(2):328–40. doi: 10.1016/j.bja.2024.11.024 (PMC11775840; doi:10.1016/j.bja.2024.11.024)
Supplement: Multimedia component 2 [file mmc2.docx]

# The characteristics of older patients undergoing surgery in the UK: a snapshot observational study - SNAP-3

### Supplementary table 12: SNAP-3 collaborators

A list of collaborators to be recognised for the input into SNAP-3.

| **Organisation** | **Collaborators** |
| --- | --- |
| **Addenbrookes Hospital** | Alexandre Field |
| Principal Investigator Svet Petkov | Amit Deshmukh |
|  | Amy Frayling |
|  | Andrea Ortu |
|  | Bogdan Petrisor |
|  | Chamika Abayasinghe |
|  | Charles Noonan |
|  | George Couch |
|  | Harsh Nimaiyar |
|  | Israa Elfouli |
|  | Joanne Outtrim |
|  | Katharina Kohler |
|  | Konul Hajiyeva |
|  | Laura Graham |
|  | Mark Forth |
|  | Mark Gotecha |
|  | Nader Alrefaii |
|  | Poppy Aldam |
|  | Priti Morzaria |
|  | Rachel Ariyanayagam |
|  | Rajan Mehta |
|  | Reece Cordy |
|  | Samudu Wahappulige |
|  | Sandeep Sharma |
|  | Sanika Patil |
|  | Shayan Arshed |
|  | Sonia Boktor |
| **Aintree Hospital** | Antony Berridge |
| Principal Investigator Dermot Moloney | Bisanth Batuwitage |
|  | Chris Marsh |
|  | Claire Davis |
|  | Dermot Moloney |
|  | Ellie Quilliam |
|  | Ellie Quillium |
|  | James Cassidy |
|  | James Pratt |
|  | Laura Bridge |
|  | Matthew Palethorpe |
|  | Melanie Harrison |
|  | Michelle Linforth |
|  | Nadim Kozman |
|  | Nicki Russell |
|  | Saskia Van |
|  | Shirley Cooper |
| **Airedale General Hospital** | Amy Kitching |
| Principal Investigator Tamsin Gregory | Andrew Pearson |
|  | Chantel Mcparland |
|  | Emma Dooks |
|  | Lisa Armstrong |
|  | Tamsin Gregory |
|  | Patrick Winstanley |
| **Arrowe Park Hospital** | Abigail Carey |
| Principal Investigator David Blackman | Billy Holt |
|  | David Blackman |
|  | Callum Hammond |
|  | Florence Wilson |
|  | Julie Grindey |
|  | Kieran Walker |
|  | Lewis Mcintyre |
|  | Molly Wyche |
|  | Natalie Ash |
|  | Sam Freeborn |
|  | Siofra Daly |
|  | Taqua Omer |
|  | Tilemachos Zaimis |
| **Ashford and St Peters Hospital** | Ana Glennon |
| Principal investigator Mark Macgregor | Caroline Ellis |
|  | Caroline Pocknall |
|  | Gayatri Saxena |
|  | Isaac John |
|  | Jigneshkumar Patel |
|  | Kashif Jabbar |
|  | Keshnie Govender |
|  | Louise Renouf |
|  | Lynn Drummond |
|  | Margaret Dsouza |
|  | Margaret Walsh |
|  | Marie Buckley |
|  | Mark Macgregor |
|  | Matilda Ravidram |
|  | Meera Nadir |
|  | Rita Pereira |
|  | Seliat Sanusi |
|  | Sophia Khaleeq |
|  | Thomas Hall |
| **Barnet General Hospital** | Ailidh Lang |
| Principal Investigator Claire King | Ben Tilley |
|  | Claire King |
|  | Emily Lachmann |
|  | Keerthi Senthil |
|  | Lucy Owen |
|  | Nael Alavi |
|  | Su Ling |
| **Barnsely Hospital** | Abigail Crewe |
| Principal Investigator Sunil Chaurasia | Alice Nicholson |
|  | Anna Galvin |
|  | Christopher Harrison |
|  | Emma Stoner |
|  | Jenny Mullan |
|  | Jodie Macdonald |
|  | Lisa Proudfoot |
|  | Natalie Lake |
|  | Nicola Lancaster |
|  | Ruth Nanda |
|  | Sarah Cuts |
|  | Sunil Chaurasia |
|  | Susan Hope |
|  | Wei Low |
| **Basildon University Hospital** | Angelo Ramos |
| Principal Investigator Pallavi Marghade | Angukumar Thangamuthu |
|  | Anne Nicholson |
|  | Chanaka Karunaratne |
|  | Claire Mccormick |
|  | Emily Redman |
|  | Gladys Emmanuel |
|  | Jean Byrne |
|  | Joanne Riches |
|  | Jo-anne Cartwright |
|  | Jonaifah Ramirez |
|  | Kai Li |
|  | Kelly Musson |
|  | Kerry Goodsell |
|  | Kriti Vig |
|  | Luke Hounsom |
|  | Lushen Pillay |
|  | Miranda Forsey |
|  | Moroti Abioye |
|  | Muhammed Faheem |
|  | Pallavi Marghade |
|  | Samuel Rowe |
|  | Sofia Alexandra |
|  | Sohail Omar |
|  | Stacey Pepper |
|  | Steph Gumus |
|  | Vivek Sharma |
| **Bedford Hospital** | Anisha Roopram |
| Principal Investigator Peter Knowlden | Joshua Mcgillicuddy |
|  | Melchizedek Penacerrada |
|  | Muhammad Haseeb |
|  | Peter Knowlden |
|  | Rachel Lorusso |
|  | Ridwan Sharif |
|  | Ruby Fronda |
| **Birmingham Heartlands, Good Hope and Solihull Hospitals** | Aaron Klair |
| Principal Investigator Joyce Yeung | Abdul Hai |
|  | Akhil Chellapuri |
|  | Alexandra Timperley |
|  | Ali Abusbaeh |
|  | Annapoorani Subramani |
|  | Ayesha Khalid |
|  | Ayisha Afzal |
|  | Caitlin Stevens |
|  | Christina Thomas |
|  | Ciara Gibson |
|  | Dan Newport |
|  | Daniel Lenton |
|  | David Brennan |
|  | Deepika Chivukula |
|  | Dineshwaran Rajendran |
|  | Eleanor Reeves |
|  | Emily Butler |
|  | Farhaana Surti |
|  | Faye Moore |
|  | Georgina Kelly |
|  | Girgis Awadalla |
|  | Harry Bhalla |
|  | Hassan Abdullah |
|  | Heather Willis |
|  | Hnin Haymahn Htun |
|  | Hoi Yan |
|  | Joanne Gresty |
|  | Joseph Son-leong |
|  | Joseph Leong-son |
|  | Joyce Yeung |
|  | Juliet Sebastian |
|  | Kiren Parkash |
|  | Komal Bodhke |
|  | Kristina Gallagher |
|  | Maahi Qureshi |
|  | Mable Kurian Chalil |
|  | Mahmoud Khedr |
|  | Manahil Bashir |
|  | Maria Ghani |
|  | Marwel Alyssa Jones |
|  | Mary Bellamy |
|  | Matt Beck |
|  | Matthew Sibley |
|  | Merna Ebrahim |
|  | Mike Seyani |
|  | Mirriam Sangombe |
|  | Mohammed Al-zubayel |
|  | Muhammad Abdulaziz |
|  | Muhammad Ansari |
|  | Muhammad Mujeeb |
|  | Murshid Ali |
|  | Murshid Ali Mohamed Maheen |
|  | Olivia Babaua |
|  | Patience Juru |
|  | Preethi George Pandeth |
|  | Rana Said |
|  | Rebecca Hatchard |
|  | Rehman Crisp |
|  | Rochelle Velho |
|  | Ruth Joslyn |
|  | Safwaan Patel |
|  | Sam Stafford |
|  | Sant Leelamanthep |
|  | Sarang Pathak |
|  | Sharvari Mahajan |
|  | Sharvari Vadeyar |
|  | Sunera Khan |
|  | Suvojit Misra |
|  | Syra Kazmi |
|  | Tammy Bellamy |
|  | Teresa Melody |
|  | Yash Tyagi |
| **Birmingham Women's Hospital** | Clare Mcpake |
| Principal Investigator Jane Pilsbury | Faye Andrews |
|  | Jane Pilsbury |
|  | Parminder Chana |
|  | Rosy Dunham |
|  | Samantha Bull |
|  | Kerry Cullis |
|  | Sandeep Kapur |
|  | Lyndon Harkett |
|  | Emma Plunkett |
|  | Tim Molitor |
| **Blackpool Victoria Hospital** | Alanna Beasley |
| Principal Investigator Anwar Ulhaq | Amy Barnett |
|  | Andrea Cinconze |
|  | Andrew Donohue |
|  | Aniko Babits |
|  | Anukiran Ravichandran |
|  | Anwar Ulhaq |
|  | Ashleigh Wignall |
|  | Audrey Hellen |
|  | Barbara Lord |
|  | Carol Jeffs |
|  | Deepa Sebastian |
|  | Denise Bennett |
|  | Dzmitry Zabauski |
|  | Emma Ward |
|  | Hannah Walsh |
|  | Jayne Windebank |
|  | Joanna Brown |
|  | John Barrett |
|  | Julia Mason |
|  | Karen Williams |
|  | Katherine Finch |
|  | Kayleigh Mountford |
|  | Kiran Nadiger |
|  | Leonie Benham |
|  | Lisa Elawamy |
|  | Marium Khan |
|  | Melanie Caswell |
|  | Natalie Irvine |
|  | Pinar Sheard |
|  | Robert Davidson |
|  | Samuel Remnant |
|  | Sarah Traynor |
|  | Scott Warden |
|  | Senka Baranovic |
|  | Shamina Hankinson |
|  | Steph Reed |
|  | Stephen Davies |
|  | Stephen Preston |
|  | Steve Wiggans |
|  | Steven Dixon |
|  | Vasanthi Vasudevan |
|  | Victoria Cunliffe |
|  | Zena Bradshaw |
| **Borders General Hospital** | Chengyuan Zhang |
| Principal Investigator Stephen Alcorn | Ella Bennett |
|  | Harriet Briggs |
|  | Heather Matthews |
|  | Katie Stewart |
|  | Michael Wild |
|  | Nick Spencer |
|  | Nicola Goldmann |
|  | Peter Carson |
|  | Rachel Harvey |
|  | Stephen Alcorn |
|  | Vanessa MacKenzie |
| **Bradford Royal Infirmary** | Edward Tam |
| Principal Investigator Robert Spencer | Estelle Tan |
|  | Louise Akeroyd |
|  | Michael Tattersfield |
|  | Michael Kitchen |
|  | Muhammad Haider |
|  | Robert Spencer |
|  | Robert Palin |
|  | Sangavy Loganathan |
|  | Satish Bharti |
|  | Simon Cousins |
|  | Thomas Knapp |
| **Bristol Royal Infirmary and St Michaels Hospital** | Amy Ashford |
| Principal Investigator Hannah Wilson | Andy Bartlett |
|  | Angeliki Kolovou |
|  | Anna Simpson |
|  | Annie Wood |
|  | Becky Woolf |
|  | Chintan Vora |
|  | Denise Webster |
|  | Georgia Efford |
|  | Hannah Wilson |
|  | Henry House |
|  | Josephine Bonnici |
|  | Kathleen Corcoran |
|  | Katie Pass |
|  | Katie Sweet |
|  | Kim Wright |
|  | Mike Peacock |
|  | Neil Choudhuri |
|  | Patrick Liddicoat |
|  | Rachael Meredith |
|  | Richard Cassar White |
|  | Steph Brown |
|  | Suzi Braggins |
|  | Tom Woodland |
|  | Will Gatfield |
| **Bronglais General Hospital** | Gabor Dudas |
| Principal Investigator Gabor Dudas | Heather Mcguinness |
|  | Ronda Loosley |
|  | Tanya Sims |
| **Broomfield Hospital** | Amanda Lyle |
| Principal Investigator Al Hughes | Anna Beckwith |
|  | Caroline Fox |
|  | Al Hughes |
|  | Christina Williams |
|  | Conor Barrett Nnochiri |
|  | Elizabeth Dawson |
|  | Joanne Wootton |
|  | Karen Cranmer |
|  | Katherine Rao |
|  | Lauren Sach |
|  | Lorraine James |
|  | Lucy Willsher |
|  | Lucy Westcott |
|  | Martina Vitaglione |
|  | Nicola Cutmore |
|  | Nicola Bulleta |
|  | Nikolett Hunyadvari |
|  | Rachel Arnold |
|  | Sharon Reid |
|  | Stacey Cotterell |
|  | Tracey Camburn |
|  | Victoria Apps |
|  | Yvonne Lester |
| **Buckinghamshire Healthcare Trust** | Abdullah Alkhudhayri |
| Principal Investigator Tamsin Mcallister & Jeremy Drake | Adam Taylforth |
|  | Adrianna Zembrzycka |
|  | Ajay Sanghvi |
|  | Alice Ngumo |
|  | Anita Cserbane |
|  | Aruna Naire |
|  | Asia Joseph |
|  | Bernard Nyemitei |
|  | Bethan Davies |
|  | Bobbie Sanghera |
|  | Geraldine Hambrook |
|  | Gregory Manning |
|  | Hannah Catton |
|  | James Goetz |
|  | James Winchester |
|  | Jeremy Drake |
|  | Jonathan Blake |
|  | Lucy Godfrey |
|  | Maja Rakic |
|  | Natalie Smith |
|  | Paula Jenkins |
|  | Ruth Penn |
|  | Saba Syed |
|  | Saranya Thurairatnam |
|  | Sonia Mariampillai |
|  | Sophie Jackman |
|  | Tamsin Mcallister |
|  | Victoria Whittaker |
| **Calderdale and Huddersfield FT** | Aaquid Akram |
| Principal Investigator Sophie Lawton & Pnt Laloe | Abigail Atkin |
|  | Alice Wheeler |
|  | Andrew Haigh |
|  | Ben Green |
|  | Bethany Spencer-lane |
|  | Eve Braithwaite |
|  | Harriet Watson |
|  | Hedd Carden |
|  | Jack Hogg |
|  | Jason Auguste |
|  | Jessica Morgan |
|  | Jithu Jayan |
|  | Jodie Bellwood |
|  | Matthew Robinson |
|  | Pnt Laloe |
|  | Sam Doyle |
|  | Sophie Lawton |
| **Charing Cross Hospital** | Ajantheny Naguleswaran |
| Principal Investigator Kenneth Murray | Ashley Guilliam |
|  | Dharshini Rajasooriyer |
|  | Elaine Viloria |
|  | Patrick Daly |
|  | Samuel Mindel |
|  | Stephanie Ivie |
|  | kenneth Murray |
|  | Vidhya Nagaratnam |
| **Chelsea and Westminster Hospital** | Alex Schoolmeesters |
| Principal Investigator Marcela Vizcaychipi | Anan Bomfim |
|  | Carina Bautista |
|  | Eleanor Giles |
|  | Elena Noval |
|  | Emily Futter |
|  | Florison Canlas |
|  | Jaime Carungcong |
|  | Kribashnie Nundlall |
|  | Laura Martins |
|  | Leah Flores |
|  | Marcela Vizcaychipi |
|  | Mari Rose |
|  | Patricia Costa |
|  | Rhian Bull |
| **Chesterfield Royal Hospital** | Adam Mendelski |
| Principal Investigator Julie Hui | Amanda Whileman |
|  | Cindy Cart |
|  | Claire Sampson |
|  | Elizabeth Blythe |
|  | Emily Jolly |
|  | Emma Moakes |
|  | Julie Hui |
|  | Katarina Djapic |
|  | Kelly Pritchard |
|  | Lauren Bishop |
|  | Leanne Lowe |
|  | Lesley Stevenson |
|  | Linda Bishop |
|  | Mary Kelly-baxter |
|  | Nicky Ford |
|  | Rachel Gascoyne |
|  | Rheanna Smith |
|  | Roshani Deorukhkar |
|  | Sarah Broadhead |
|  | Stephanie Wright |
|  | Thomas Hughes-gooding |
| **Colchester Hospital** | Alison Ghosh |
| Principal Investigator Joanna Simpson | Alison O'kelly |
|  | Anita Immanuel |
|  | Ashley Elden |
|  | Bindiya Shah |
|  | Celine Driscoll |
|  | Devraj Kathwadia |
|  | Edyta Klata |
|  | Emma Williams |
|  | Hazel Yeoh |
|  | Hnin Shwe |
|  | Jennifer Abaddulay |
|  | Joanna Simpson |
|  | Justyna Kapera |
|  | Kali-jade Gunfield |
|  | Katrina Cooke |
|  | Marianne Morgan |
|  | Michelle Dotchin |
|  | Nyasha Nago |
|  | Samuel Rowles |
|  | Sara Scott |
|  | Tracy Abery |
| **Countess of Chester Hospital** | Alex Moore |
| Principal Investigator Woei Lin Yap | Chloe Haylett |
|  | Chloe Donaldson |
|  | Conor Steele |
|  | Hannah Mulgrew |
|  | Imogen Watkins |
|  | Kieran Kelly |
|  | Laura Wilson |
|  | Naomi Slater |
|  | Nick Roberts |
|  | Woei Lin Yap |
|  | Yahya Abdullah |
| **Croydon University Hospital** | Agnes Fong |
| Principal Investigator Agnes Fong | Alexander Foreman |
|  | Ashok Sundar |
|  | Christopher Cregg |
|  | Darren Caldow |
|  | Jennifer Haugh |
|  | Laura Ashton-Edwards |
|  | Natashia Schneider |
|  | Rana Mallah |
|  | Reena Khade |
|  | Sheun Fang Cheng |
|  | Sonali Mohite |
|  | Sonia Rasoli |
|  | Vasileios Bafitis |
| **Cumberland Infirmary, Carlisle** | Emma Mawson |
| Principal Investigator Geetanjali Verma | Geetanjali Verma |
|  | Laura Chapman |
|  | Theresa Cooper |
| **Darent Valley Hospital** | Asad Zafar |
| Principal Investigator Mansoor Sange | Bridget Fuller |
|  | Caspar Briault |
|  | Conor Walsh |
|  | Daniel Lake |
|  | Ee Lyn Chan |
|  | Georgia Monantera |
|  | Hoda AbouGhoneim |
|  | Mansoor Sange |
|  | Matthew Smith |
|  | Medappa Kaliyanda |
|  | Naomi Oakley |
|  | Nesma Abdelaziz |
|  | Olumide Olufuwa |
|  | Rajendra Pun |
|  | Sara Yousaf |
|  | Sean Cardoso |
|  | Sean Warburton |
|  | Shamini Sivakumaran |
|  | Sophie Hill |
|  | Sze So |
| **Darlington Memorial Hospital** | Amanda Cowton |
| Principal Investigator Victoria Craig | Andrew Shepperson |
|  | Christopher Taylor |
|  | Clare Hutton |
|  | David Ginty |
|  | Raviprakesh Hodigere |
|  | Roshan Sebastian |
|  | Sally Roscoe |
|  | Sanjeev Kumar |
|  | Victoria Craig |
|  | Zachary Bowen-Davies |
| **Derriford Hospital** | Alice King |
| Principal Investigator Gavin Werrett | Alina Van-Hien |
|  | Amy Turner |
|  | Andy Savva |
|  | Anna Ratcliffe |
|  | Anneliese Crome |
|  | Chris Gordon |
|  | Elaine Jones |
|  | Emily May |
|  | Emma Bishop |
|  | Fiona Reed |
|  | Gary Minto |
|  | Gavin Werrett |
|  | Gunarathna Perumbadage |
|  | Helen Anderson |
|  | Holly Notman |
|  | Jennifer Moran |
|  | Juleen Fasham |
|  | Julie Alderton |
|  | Karen Friendship |
|  | Kelly Whitehorn |
|  | Lorraine Madziva |
|  | Louise Jose |
|  | Lucy Guile |
|  | Martin Mills |
|  | Memory Mwadeyi |
|  | Natasha Wilmshurst |
|  | Rebecca Allott |
|  | Shivesh Tewari |
|  | Shun Yamanaka |
|  | Tracey Ward |
|  | Will Hare |
|  | William Peagam |
| **Doncaster Hospital** | Alasdair Strachan |
| Principal Investigator Alasdair Strachan | Angela Waddingham |
|  | Emily Hall |
|  | Fiona Dunning |
|  | Gemma Rook |
|  | Kerry Dooley |
|  | Lisa Warren |
|  | Rebecca Pugh |
|  | Sarah Farmer |
|  | Thomas Bidmead |
|  | Vikki Cooke |
| **East Surrey Hospital** | Antony Ratnasingham |
| Principal Investigator Anthony Ratnasingham | Edward Combes |
|  | Ellen Jessup-dunton |
|  | Emily Watts |
|  | Gail Murphy |
|  | Indhuja Rajkumar |
|  | Julie Houghton |
|  | Leigh-james Spurling |
|  | Lisa Clutterbuck |
|  | Louise Nimako |
|  | Merlin James |
|  | Michele Poole |
|  | Nancy Jones |
|  | Ruth Habibi |
|  | Sallyanne Trotman |
|  | Samantha Weller |
|  | Sarah Davies |
|  | Sophie Beverley |
|  | Sophie Holden |
|  | Valerie Beech |
|  | Yvonne Izzard |
| **Eastbourne District General Hospital** | Amr Elmosalamy |
| Principal Investigator Tara Bolton | Charlotte Crossland |
|  | James Hartley |
|  | Janet Sinclair |
|  | Matthew Farrant |
|  | Nicola Deacy |
|  | Paul Jackson |
|  | Penny Boxall |
|  | Ross Holcombe-law |
|  | Sang Yob |
|  | Tara Bolton |
|  | Thomas Kavanagh |
|  | Timothy Faccini |
|  | Toni De Freitas |
| **Edinburgh Royal Infirmary** | Ajit Singh Obhrai |
| Principal Investigator Thomas Ballantyne | Anna Te |
|  | Arlena Kuenzel |
|  | Bartlomiej Ordys |
|  | Elizabeth Steel |
|  | Emma Mann |
|  | Flora Mclennan |
|  | Kirsten Reid |
|  | Kwun Chan |
|  | Lisa Dewar |
|  | Michael Robson |
|  | Mysoon Alabdah |
|  | Naomi Hyndman |
|  | Rosanna Seatter |
|  | Rosemary Mudie |
|  | Sarah Scott |
|  | Thomas Ballantyne |
| **Epsom and St Helier University Hospitals** | Abi Coe |
| Principal Investigator Martin Akioyame | Adil Hussain |
|  | Agyapong Ansu |
|  | Ahmed Eid |
|  | Alice Hipsey |
|  | Alice Pandaan |
|  | Analyn Alipustain |
|  | Anna Cebula |
|  | Anna Forbes |
|  | Asmaa Hassan |
|  | Beatrix Sari |
|  | Dipak Niroula |
|  | Eva Garcia |
|  | Harriet Asquith |
|  | Irrum Afzal |
|  | Jayadeep Sandhu |
|  | Jenny Hisole |
|  | Joe Joseph |
|  | Kimberly Edgerton |
|  | Krystie Vedat |
|  | Kulasekar Kaliappan |
|  | Laijee Benny |
|  | Luke Parker |
|  | Mahmoud Elhefnawy |
|  | Mahmoud Hassanin |
|  | Martin Akioyame |
|  | Mary Madden |
|  | Maureen Estrada |
|  | Neringa Vilimiene |
|  | Niketa Shukla |
|  | Niketa Shukla |
|  | Noor Ul Islam Syed |
|  | Osatohanmwen Osagie |
|  | Rachel Hayre |
|  | Rosavic Chicano |
|  | Sandhya Anandkumar |
|  | Saurabh Kakkar |
|  | Serah Duro |
|  | Sharad Sinha |
|  | Sonia Andleeb |
|  | Suman Biswas |
|  | Thomas Craven |
|  | Tina Raju |
|  | Uju Roskon-rapu |
|  | Wadzanai Mupaya |
| **Glangwili General Hospital** | Abdul Rahim Ali Bakhsh |
| Principal Investigator Peter Havalda | Adam Ryan |
|  | Barnaby Hart |
|  | Becky Icke |
|  | Dylan John |
|  | Jill Williams |
|  | Linda O'brien |
|  | Lucy Hill |
|  | Michael Coulton |
|  | Peter Havalda |
|  | Samantha Coetzee |
| **Glasgow Royal Infirmary and Stobhill Ambulatory Care Hospital** | Chris Hay |
| Principal Investigator Malcolm Howell and Sonya Mckinlay | Eilidh Lynch |
|  | Emma Bhatti |
|  | Gemma Scotland |
|  | Khalid Boussouara |
|  | Malcolm Howell |
|  | Marielle Li |
|  | Mykhaylo Shumeyko |
|  | Roshan Saleh |
|  | Ryan Murphy |
|  | Sarah Barton |
|  | Sonya Mckinlay |
|  | Susan Speirs |
|  | Susanne Cathcart |
| **Glenfield Hospital** | Adela Dobru |
| Principal Investigator Rahil Mandalia | Aqsa Haq |
|  | Emily Rich |
|  | Frances Tait |
|  | Hayun Lee |
|  | Jaimin Arya |
|  | Joanna Shak |
|  | Jun Tan |
|  | Kathleen Wolff |
|  | Pitchayud Kantachuvesiri |
|  | Rajani Annamaneni |
|  | Rohan Babla |
| **Gloucestershire Hospital NHS FT** | Alex Christie |
| Principal Investigator Henry Murdoch | Artemis Prevot |
|  | Benedict Shinner |
|  | Charlotte Bestwick |
|  | Daniel Liu |
|  | Deborah Ward |
|  | Duncan Castle |
|  | Elena Teh |
|  | Ellie Courtney |
|  | Emily Farrow |
|  | Fiona Macrae |
|  | Gemma Gardner |
|  | Gemma Gardener |
|  | Helena Veck |
|  | Henry Murdoch |
|  | Hiromi Uzu |
|  | Isabel Evans |
|  | Jayne Evitts |
|  | Joanne Waldron |
|  | Kayleigh Collins |
|  | Kirsty Benton |
|  | Matthew Townsend |
|  | Maximo Clark |
|  | Ozzie Zaidi |
|  | Pauline Brown |
|  | Reggie Gray |
|  | Rhiannon Tanner |
|  | Sophie Turnton |
|  | Susan O'connell |
|  | Tushar Rakhecha |
|  | Victoria Gaunt |
| **Great Western Hospital** | Abbie Poole |
| Principal Investigator Mala Greamspet | Catherine Novis |
|  | Mala Greamspet |
|  | Charlotte Hunt |
|  | Dominic Bashford |
|  | Helen Langton |
|  | Jacinta Ugoji |
|  | James Sharples |
|  | James Brooks |
|  | Joe Stevens |
|  | Laura McAfferty |
|  | Maggie Ryder |
|  | Nicholas Budhram |
|  | Rachel Lyons |
|  | Rebecca Mairs |
|  | Robert Coe |
|  | Tracey Benn |
| **Guys and St Thomas** | Abegail Salvana |
| Principal Investigator Kariem El-boghdadly | Alexander Ware |
|  | Andrew Williams |
|  | Andrew Wilson |
|  | Anthi Andrianou |
|  | Azka Afzal |
|  | Bhavini Shah |
|  | Danny Wong |
|  | David Hutchinson |
|  | Eleni Eleni |
|  | Gary Colville |
|  | Gill Arbane |
|  | Jack Jack |
|  | John Paul Mcnally-reilly |
|  | Joseph Hetherington |
|  | Kariem El-boghdadly |
|  | Liana Zucco |
|  | Maame Adesu Poku |
|  | May Rabuya |
|  | Naina Mohan |
|  | Paul Morillon |
|  | Phoebe Scarfield |
|  | Rachel Babic |
|  | Rosalind Rosalind |
|  | Rupert Mason |
|  | Saif Ahmed |
|  | Sanna Khawaja |
|  | Sara Ko |
|  | Sarah Eshelby |
|  | Sherpal Singh |
|  | Sofiane Kouadria |
|  | Thomas Potter |
|  | Thwe Han |
| **Hammersmith Hospital** | Amitav Philip |
| Principal Investigator Kenneth Murray | Frances Garrick |
|  | Joshua Singleton |
|  | Peter Zsinko |
|  | Slawomir Jaszczuk |
|  | Uchechi Nwangama |
| **Harrogate District Hospital** | Abhinav Kant |
| Principal Investigator Abhinav Kant | Ahmed Aboughazy |
|  | Caroline Bennett |
|  | Karim Elfaham |
| **Hillingdon Hospital** | Geraldine Landers |
| Principal Investigator Myra Malik and Robert Crooks | Melinda Holden |
|  | Myra Malik |
|  | Natasha Mahabir |
|  | Robert Crooks |
|  | Stephen John |
| **Homerton University Hospital** | Amrutha Vishwanathan |
| Principal Investigator Christian Schwiebert | Christian Schwiebert |
|  | Deepthy Pillai |
|  | Eftychia Sousi |
|  | Eleanor Taylor |
|  | Jagrul Miah |
|  | Jenny Olsson |
|  | Pramitha Chinduluri |
|  | Rose English |
|  | Samuel Naylor |
|  | Saqib Khawaja |
|  | Srinivasan Perumal |
|  | Tay-yibah Mohamed |
|  | Zain Syed |
| **Hull Royal Infirmary** | Abigail Lau |
| Principal Investigator Andrew Gratrix | Adam Walker |
|  | Andrew Gratrix |
|  | Barbara Ribeiro |
|  | Christopher Macrow |
|  | Elizabeth Stones |
|  | Emily Frost |
|  | Esme Ward |
|  | Eugene Ndimele |
|  | Hannah Lauder |
|  | Harriet Van |
|  | Hull Research |
|  | Jakub Kazda |
|  | Jennifer Chalmers |
|  | Jonathan Payne |
|  | Jonathan Pyatt |
|  | Jookyung Park |
|  | Karen Winter |
|  | Llucia Cabral-ortega |
|  | Matthew Hines |
|  | Mithilia Govind |
|  | Molly Janowski |
|  | Nicola Staples |
|  | Prakash Subramaniam |
|  | Raquel Costantino-duarte |
|  | Rebecca Ireson |
|  | Stephanie Bailey |
|  | Stephen Mcaleer |
|  | Vicky Martinson |
|  | Llucia Cabral-Ortega |
| **Ipswich Hospital** | Aleix Ugalde |
| Principal Investigator Elizabeth Speirs | Carol Buckman |
|  | Cathleen Chabo |
|  | Ceren Driver |
|  | Charlotte Mathur |
|  | Daniel Watkins |
|  | Daniel Waldschutz |
|  | Deborah Beeby |
|  | Elizabeth Speirs |
|  | Genessa Peters |
|  | Georgina Gray |
|  | Jenny Finch |
|  | Laurence Suckling |
|  | Nipun Mundkur |
|  | Rebecca Francis |
|  | Stephanie Bell |
|  | Suchona Hafiz |
|  | Teresa Theobald |
|  | Vanessa Rivers |
|  | Victoria Fernando |
| **James Paget University Hospital** | Amanda Ayers |
| Principal Investigator Sudha Garg | Amy Garrod |
|  | Charlotte Kelly |
|  | Christian Alcock |
|  | Christian Hacon |
|  | Daniel Sciberras |
|  | Darylile Guledew |
|  | Donna Griffiths |
|  | Elva Wilhelmsen |
|  | Helen Sutherland |
|  | Julie North |
|  | Kevin Howard |
|  | Lisa Hudig |
|  | Pablov Zamora |
|  | Pablov Zamora |
|  | Sarah Daniels |
|  | Sophie Cook |
|  | Sudha Garg |
|  | Syed Mazhar |
|  | Wendy Harrison |
|  | Zainab Najim |
| **Kent and Canterbury Hospital** | David Loader |
| Principal Investigator Srdjane Trajkovic | Gemma Hector |
|  | Madhushika Dayarthne |
|  | Rachel Vernall |
|  | Srdjane Trajkovic |
|  | Ritoo Kapoor |
| **Kettering General Hospital** | Alistair Thomas |
| Principal Investigator Satya Jakkampudi | Alvin Lau |
|  | David Jarrold |
|  | Foteini Christodouli |
|  | Gayan Dissanayake |
|  | Haider Zahur |
|  | Julie Sebastian |
|  | Lauren Hunt |
|  | Paul Swift |
|  | Satya Jakkampudi |
|  | Su Vern Lim |
|  | Tony Talhat |
| **Kings College Hospital, Denmark Hill** | Amy Dukoff-gordon |
| Principal Investigator Ravi Bhatia | Andy Chu |
|  | Anna Naito |
|  | Bethan Iikponmwosa |
|  | Hamish Baillie |
|  | Jennifer Berg |
|  | Natasha Amaradasa |
|  | Ravi Bhatia |
|  | Sanjoy Bhattacharyya |
| **Kingston Hospital** | Camilla Paget |
| Principal Investigator Sarang Puranik | Elizabeth Evans |
|  | Lucy Studd |
|  | Lydia Weiss |
|  | Sarang Puranik |
| **Lancashire Teaching Hospitals** | Abdelaziz Abdelaziz |
| Principal Investigator Zara Townley, Arumugam Pitchiah | Ailsa Watt |
|  | Alexandra Williams |
|  | Amanda Alty |
|  | Angela Yan |
|  | Anil Kumar |
|  | Arumugam Pitchiah |
|  | Benjamin Stewart |
|  | Joy Hirst |
|  | Louis Turrell |
|  | Mark Verlander |
|  | Michael Roach |
|  | Nadeem Jamal |
|  | Nadia Paes |
|  | Sandra Sowden |
|  | Shalil Henderson |
|  | Shivani Gulati |
|  | Sourav Mahajan |
|  | Thomas Bradley |
|  | Zara Townley |
| **Leeds Teaching Hospital NHS Trust** | Aneesha Qadeer |
| Principal Investigator Simon Howell | Antonio Borrelli |
|  | Beverley Jackson |
|  | Caroline Thomas |
|  | Catherine Moriarty |
|  | Chandan Gupta |
|  | Ian Chadderton |
|  | Jagadish Gourapoura |
|  | Judith Sharp |
|  | Maheeka Rajamuni |
|  | Michelle Naylor |
|  | Mohannad Mohyeldin |
|  | Niranjala Wickramasinghe |
|  | Rosie Wragg |
|  | Samuel Craven |
|  | Samuel Richards |
|  | Sarah Peacock |
|  | Serena Yen |
|  | Simon Howell |
| **Leicester General Hospital** | Amy Howard |
| Principal Investigator Rahil Mandalia | Ankit Darolia |
|  | Chris Molloy |
|  | Francesca O'Brien |
|  | Gamal Ibrahim |
|  | Irina Georgieva |
|  | Matt Gawne |
|  | Puspinder Kaur |
|  | Richard Pertwee |
|  | Taha Namik |
|  | Thomas Chad |
|  | Vipul Kaushik |
|  | Yuvraj Kukreja |
| **Leicester Royal Infirmary** | Anish Khandia |
| Principal Investigator Rahil Mandalia | Anthony Wyn-hebden |
|  | Beth Leonard |
|  | Busu Zvidzayi |
|  | Cailin Mcevoy |
|  | Charlotte Hall |
|  | Dan Walker |
|  | Dhruti Pandya |
|  | Diane Jackson |
|  | Emily Parker |
|  | Jack Hague |
|  | Jamie Macdonald |
|  | Jen Taylor |
|  | Kirsty Macfarlane |
|  | Lisa Mclelland |
|  | Mohamed Alebsawy |
|  | Mohamed Shahbudin |
|  | Nathan Ware |
|  | Paras Patel |
|  | Prematie Andreou |
|  | Rahil Mandalia |
|  | Rebecca Cole |
|  | Sharon Tam |
|  | Sobia Jahan |
|  | Syed Hussain |
|  | Wahaballah Abdallah |
|  | Zaid Ahmad |
| **Leighton Hospital** | Beata Lawecka |
| Principal Investigator Helen Burton | Claire Gabriel |
|  | Deborah Maren |
|  | Emily Lear |
|  | Helen Burton |
|  | Hewakilpitige Ranaweera |
|  | Juraj Hajnik |
|  | Katherine Pagett |
|  | Laura Ellerton |
|  | Przemyslaw Lawecki |
|  | Rajanbabu Nivethana |
|  | Sanjeewa Ranaweera |
|  | Sheron Clarke |
|  | Victoria Williams |
|  | William Gray |
| **Lincoln County Hospital** | Arion Pepas |
| Principal Investigator Manish Kakkar | Crystal Yick |
|  | Frances Wilson-Morkeh |
|  | Kieran Poland |
|  | Lydia Pegman |
|  | Mark Chen |
|  | Rosanna Baker-Wilding |
|  | Sarah Farrington |
|  | Shawn Miranda |
|  | Manish Kakkar |
|  | Nicole Sarens |
|  | Shivan Kanani |
| **Lister Hospital** | Anna Price |
| Principal Investigator Pietro Ferranti | Carina Cruz |
|  | Christopher Eyeington |
|  | Darren Smith |
|  | Gloria Sikapite |
|  | James Noble Johnston |
|  | Joy Derigay |
|  | Martin Ebon |
|  | Pietro Ferranti |
|  | Rhos Gabriel |
|  | Sachin Navarange |
|  | Saniyah Shaikh |
|  | Sheena Lim |
| **Liverpool Womens Hospital** | Adam Mcconville |
| Principal Investigator Grainne Garvey | Amy Beasley |
|  | Amy Hughes |
|  | Ashoke Shah |
|  | Asim Khan |
|  | Edward Staunton |
|  | Grainne Garvey |
|  | Helen Mcnamara |
|  | John Osakue |
|  | Katrina Reily |
|  | Oliver Henry |
| **Manchester Royal Infirmary (MRI)** | Adel Hutchinson |
| Principal Investigator Hannah Greenlee | Alistair Duncan |
|  | Amarjeet Patil |
|  | Anila Sukumaran |
|  | Deborah Paripoorani |
|  | Dominique Jones |
|  | Hannah Greenlee |
|  | Holly Moxon |
|  | Iain Venables |
|  | Lauren Edmunds |
|  | Melanie Barker |
|  | Rahul Norawat |
|  | Rajit Khosla |
|  | Richard Clark |
|  | Shazra Reeza |
|  | Sujesh Bansal |
| **Medway Maritime Hospital** | Ahmed Rashwan |
| Principal Investigator Keith Lankester | Aimee Williams |
|  | Andreas Sotirieau |
|  | Gayzel Vallejera |
|  | Jennifer May |
|  | Jodie Wright |
|  | Keith Lankester |
|  | Laura Kemp |
|  | Linda Ofori |
|  | Lisa Parker |
|  | Lucy Connolly |
|  | Mary Everett |
|  | Mohammed Aktar Rezaur Rahman |
|  | Petros Akin-nibosun |
|  | Rebecca Collins |
|  | Sabita Pokharel |
|  | Sheena Cheung |
|  | Sheldon Ferron |
|  | Sian Wilson |
|  | Sureswarasarma Jagatheepan |
|  | Thomas Bolland |
|  | Tom Hatton |
|  | Vandana Rajesh |
| **Manchester University NHS FT (except MRI)** | Afeefa Rasheeth |
| Principal Investigator Sujesh Bansal | Alexander Scott |
|  | Sujesh Bansal |
|  | Alistair Sawyerr |
|  | Amr Shalaby |
|  | Andy Fairclough |
|  | Angela Chrisopoulou |
|  | Charlotte Taylor |
|  | Daniel Conway |
|  | Despoina Toraki |
|  | Dhirendra Allen |
|  | Dominque Jones |
|  | Hannah Bennett |
|  | Heather Mcmullen |
|  | Helen Michael |
|  | Helen T-michael |
|  | Jane Shaw |
|  | Jees Porinch |
|  | Jo-ann Midgley |
|  | Joanne Rothwell |
|  | Jossy Kayappurathu |
|  | Kailash Bhatia |
|  | Karan Kanal |
|  | Karen Connolly |
|  | Lisa Cooper |
|  | Lok Heng Arosa Lin |
|  | Lorna Oshea |
|  | Neeraj Bhardwaj |
|  | Nicholas Gould |
|  | Nisha Jayanandan |
|  | Nowfal Rahman |
|  | O'baird Haider |
|  | Olga Colaco |
|  | Oliver Hill |
|  | Peter Alexander |
|  | Preetha Mathew |
|  | Rose Jama |
|  | Sarah Beresford |
|  | Sarah Yousif |
|  | Sharon Baxter-dore |
|  | Sheetal Crasta |
|  | Sofia Fioumi |
|  | Stefania Stewart |
|  | Steph Harrison |
|  | Susan Ferguson |
|  | Thomas Morris |
|  | Tracey Hodgkiss |
| **Mid Yorkshire Hospital NHS Trust** | Alexandra Metcalfe |
| Principal Investigator Brendan Sloan | Amit Dalvi |
|  | Amy Longhurst |
|  | Amy Major |
|  | Brendan Sloan |
|  | Chuyan Yu |
|  | Elizabeth Denis |
|  | John Norris |
|  | Lauren Tye |
|  | Lucy Gurr |
|  | Martin Sylvester |
|  | Nathan Sloane |
|  | Nicholas Wroe |
|  | Rusha Saha |
|  | Sarah Buckley |
|  | Sharwend Supermanian |
|  | Susannah Thomas |
|  | Theo Perkins |
| **Milton Keynes University Hospital** | Andrew Jones |
| Principal Investigator Richard Stewart | Anne Rose |
|  | Arun Mukkavilli |
|  | Cheryl Padila-harris |
|  | Diane Scaletta |
|  | Eva Howard |
|  | Francesca Teasdale |
|  | Jeannette Smith |
|  | Jimmy John |
|  | Louise Mew |
|  | Louise Moran |
|  | Mirajini Manoharan |
|  | Mustafa Majeed |
|  | Paramdeep Jandu |
|  | Rahim Esmail |
|  | Ramiro D'abrantes |
|  | Rashmi Rebello |
|  | Rhys Smith |
|  | Richard Stewart |
|  | Sara-beth Sutherland |
|  | Shalini Patel |
|  | Sue George |
|  | Veronica Edgell |
| **Moorfields Eye Hospital** | Floju Chin |
| Principal Investigator Louisa Pavlakovic | Louisa Pavlakovic |
|  | Mariepi Manolis |
|  | Sejung Park |
|  | Stefanos Ioannidis |
| **Morriston Hospital** | Abish Kodakkat |
| Principal Investigator Shilpa Rawat | Alex Wilson-evans |
|  | Caroline Davies |
|  | Christine Range |
|  | Craig Sell |
|  | Debra Evans |
|  | Doaa Awadallah |
|  | Fahad Salim |
|  | Fatima Lahloub |
|  | Helen Williamson |
|  | James Ainsworth |
|  | James Bowen |
|  | Jenny Travers |
|  | Kristian Dye |
|  | Lee Gauntlet |
|  | Marie Williams |
|  | Mark Baker |
|  | Mohammed Hassan |
|  | Mostafa Elsayed |
|  | Richard Hughes |
|  | Sharon Storton |
|  | Shilpa Rawat |
|  | Sophie Jones |
|  | Sophie Tate |
|  | Tim Green |
| **Musgrove Park Hospital** | Anna Tennant |
| Principal Investigator Rebecca Purnell | Ashly Thomas |
|  | Charmaine Shovelton |
|  | Gemma Chilcott |
|  | Kate James |
|  | Rebecca Purnell |
|  | Sharon Bates |
|  | Wayne Battishill |
| **National Hospital for Neurology and Neurosurgery** | Archana Depala |
| Principal Investigator Eleanor Carter | Eleanor Carter |
|  | Astri Luoma |
|  | Emily Awana |
|  | Emma-Jane Smith |
|  | Megan Griffiths |
|  | Michelle Lam |
|  | Robert John |
|  | Thomas O'Dell |
|  | Zainab Hussein |
| **Newcastle upon Tyne Hospitals NHS Foundation Trust** | Arankumar Sivasubramaniam |
| Principal Investigator David Saunders | Arathi Radhakrishnan |
|  | Arti Gulati |
|  | Ben Brown |
|  | David Saunders |
|  | Emma Grace Lewis |
|  | Fatima Simoes |
|  | Grace Lewis |
|  | Hazem Altriagy |
|  | Helen Doherty |
|  | James Savage |
|  | Janaki Pearson |
|  | Jonathan Dinsmore |
|  | Lauren Butler |
|  | Leigh Dunn |
|  | Luzgie Gavina |
|  | Munira Zogaib |
|  | Paula Gomez |
|  | Rikzing Bhutta |
|  | Shin Chia |
|  | Sophie Ingham |
|  | Suzanne Oneill |
|  | Suzy O'Neill |
|  | Tess Wilkinson |
|  | Thomas Wooten |
|  | Tom Wootten |
|  | Wirginia Bada |
|  | Zahid Khan |
| **Newham Hospital** | Aleksandra Laguna |
| Principal Investigator Bavesh Gohil | Bhavesh Gohil |
|  | Cherry Jain |
|  | Esme Ingram |
|  | Harkiran Sagoo |
|  | Himani Murdeshwar |
|  | Joseph Gafton |
|  | Kathy Lok-Yu Man |
|  | Labbeka Begum |
|  | Maryam Chaudhry |
|  | Niraj Barot |
|  | Rebecca Carroll |
|  | Sameh Shafek |
|  | Serena Chanoch |
|  | Swati Bansal |
|  | Tim Westwood |
|  | Zohreh Abdi |
| **NHS Tayside** | Alison Mcculloch |
| Principal Investigator Sharon Hilton-Christie | Callum Taylor |
|  | Chloe Chang |
|  | Christopher Perman |
|  | David Johnstone |
|  | Duncan Hargreaves |
|  | Eilidh Gillen |
|  | Fiona Burns |
|  | Jennifer Lockhart |
|  | Joanna Tait |
|  | Joaquim Desousa |
|  | Jonathan Miller |
|  | Kirsty Morrison |
|  | Lewis Macleod |
|  | Nicholas Record |
|  | Paul Martin |
|  | Paul Wasik |
|  | Rachel Philips |
|  | Ross Hendry |
|  | Samantha Dean |
|  | Sarah Bugeja |
|  | Sean Sproule |
|  | Sharon Hilton-Christie |
|  | Simon Parkin |
|  | Tim Smith |
|  | Victoria Richmond |
| **Norfolk and Norwich University Hospital** | Akshita Daga |
| Principal Investigator Melanie Maxwell | Alex Yusaf |
|  | Carla White |
|  | Caroline Reavley |
|  | Carolyn Dales |
|  | Daniel Teszka |
|  | David Brooks |
|  | Dominic Linden |
|  | Ewa Prusak |
|  | Gemma Maryan |
|  | Harriette Beard |
|  | Hazem Kamel |
|  | James Long |
|  | Jonathan Dearden |
|  | Karan Verma |
|  | Kate Tabrett |
|  | Laura Hobbs |
|  | Meghan Jones |
|  | Melanie Maxwell |
|  | Nancy Wang |
|  | Nicky Ueckermann |
|  | Peter Locke |
|  | Sebastian Locke |
| **North Middlesex University Hospital** | Bhamini Tharamalingam |
| Principal Investigator Hemantha Handapangoda | Dinesh Vidanagamage |
|  | Farham Rasheed |
|  | Godknows Mashaire |
|  | Hemantha Handapangoda |
|  | Jane Benedict |
|  | Kugan Xavier |
|  | Rebecca Hull |
|  | Rizana Ghafoor |
| **University Hospital of North Tees** | Alison Chilvers |
| Principal Investigator David Pritchard | Carol Adams |
|  | David Pritchard |
|  | Deborah Wilson |
|  | Elaine Siddle |
|  | Elizabeth Parkes |
|  | Emma Connell |
|  | Fe Hernandez |
|  | Gala Stevanovic |
|  | Helen Wardle |
|  | Jasmine Wilkinson |
|  | Liz Baker |
|  | Lorna Shepherd |
|  | Sarah Purvis |
| **North Tyneside General Hospital** | Angela Dawson |
| Principal Investigator Adrian Taylor | Chris Yates |
|  | Gemma Mccafferty |
|  | Adrian Taylor |
|  | Hayley Mckie |
|  | Jessica Bell |
|  | Stacey Short |
| **North West Anglia NHS FT** | Ben Straughan |
| Principal Investigator Shiny Sivanandan and Sivaprakash Vaitheeswaran | Chiamaka Oladipo |
|  | Chloe Eddings |
|  | Claire Chisenga |
|  | Cristina Constantin |
|  | Eleanor Smith |
|  | Eleonora Gkigkelou |
|  | Elizabeth Clayton |
|  | Helen Bowyer |
|  | Islam Hamed |
|  | Janki Bhayani |
|  | John Frazer |
|  | Karen Scholes |
|  | Kate Fitzpatrick |
|  | Krishma Adatia |
|  | Lauren Eadie |
|  | Loredana Sescu |
|  | Lucy Dunn |
|  | Michelle Bone |
|  | Mukur Ghosh |
|  | Nicola Parker |
|  | Ping Coutts |
|  | Raquel Calcada |
|  | Roberta De |
|  | Sarah Tester |
|  | Shiny Sivanandan |
|  | Sivaprakash Vaitheeswaran |
|  | Susan O'sullivan |
|  | Sushma Ojha |
|  | Susie Osullivan |
|  | Terri-Anne Baker |
|  | Vikas Saxena |
|  | Zarah Brown |
| **Northampton General Hospital** | Alexander Nottingham |
| Principal Investigator Prashant Kakodkar | Amaryl Jones |
|  | Andrea Kempa |
|  | Catherine Wilde |
|  | Claire Woolhouse |
|  | Dave Bella |
|  | Ethelwolda Goyena |
|  | Flora Gallamoza |
|  | Jake Pile |
|  | Jane Ocallaghan |
|  | Kate Smith |
|  | Kathryn Hall |
|  | Lorraine Campey |
|  | Lucy Dudgeon |
|  | Lynne Stockham |
|  | Malgorzata Polnik |
|  | Maxine Foo |
|  | Paula Oconnell |
|  | Prashant Kakodkar |
|  | Rachael Hitchcock |
|  | Rachel Tighe |
|  | Shesly Jose |
| **Nottingham University Hospitals NHS Trust** | Ahmed Elwakil |
| Principal Investigator David Hewson | Alice Williams |
|  | Amy Mitchell |
|  | Andrew Orsi |
|  | Babar Riaz |
|  | Basma Adiel |
|  | Craig Smith |
|  | David Hewson |
|  | Hannah Dudhill |
|  | Henry Corner |
|  | Jennifer Bowen |
|  | Kelly Williams |
|  | Maryam Umar |
|  | Shameek Datta |
|  | Shannon Boardman |
|  | Shannon Gawley |
|  | Siti Abd Hadi |
|  | Thomas Horne |
|  | Viresh Patel |
|  | Vitul Manhas |
|  | Ying Chean Haw |
| **Orpington Hospital** | Ahmed Elfaioumy |
| Principal Investigator Bavesh Gohil | Michael Ayres |
|  | Osokoya Babatunde |
| **Oxford University Hospitals NHS Trust** | Akshay Shah |
| Principal Investigator James Day | Alex Filby |
|  | Eyad Abdeljawad |
|  | Georgina Wilson |
|  | Grace Readion |
|  | James Day |
|  | Jean Wilson |
|  | Joy Edwards |
|  | Karen Clark |
|  | Kin Lam |
|  | Luke Holdsworth |
|  | Mukunthakrishnan Lingeswaran |
|  | Neil Davidson |
|  | Nissy George |
|  | Peter Chater-lea |
|  | Sally Beer |
|  | Soyamol Mathew |
|  | Susan Johnston |
|  | Victoria Green |
|  | Vishaka Kerner |
| **Pilgrim Hospital** | Bryony Saint |
| Principal Investigator Lisa Sharp | Khaled Ahmed |
|  | Kimberley Netherton |
|  | Kinga Szymiczek |
|  | Lisa Sharp |
|  | Tianzhe Wong |
|  | Trish Tsuro |
| **Poole General Hospital** | Charlotte Humphrey |
| Principal Investigator Henrik Reschreiter | Charolotte Barclay |
|  | Claire Osey |
|  | Emma Langridge |
|  | Henrik Reschreiter |
|  | Judith Dube |
|  | Maxine Ashton |
|  | Megan Woolcock |
|  | Patrick Covernton |
|  | Patrick Coverton |
|  | Rebecca Miln |
|  | Yasmin De'Ath |
| **Portsmouth Hospital NHS FT (Queen Alexandra Hospital)** | Ahmed Abdelhadi |
| Principal Investigator Renee Ford | Ayesha Shajpal |
|  | Barnaby Jafkins |
|  | Charlotte Bellis |
|  | Connor James |
|  | Daniel Growcott |
|  | Guy Slabbert |
|  | Heidi See |
|  | Hermione Tolliday |
|  | James Collis |
|  | James Connor |
|  | James Gray |
|  | Karen Hudson |
|  | Katherine Pavel |
|  | Mahesh Chandrashekaraiah |
|  | Megan Adams |
|  | Mina Narouz |
|  | Natasha Hughes |
|  | Neha Hasija |
|  | Nicola Mundy |
|  | Nina Szarazova |
|  | Noel Kithakye |
|  | Nosheen Younas |
|  | Rebecca Smart |
|  | Renee Ford |
|  | Shiv Vohra |
|  | Snigdha Seksaria |
|  | Thomas Mankelow |
|  | Timothy Prescott |
|  | Yuen Kang |
| **Prince Charles Hospital** | Alysha Hancock |
| Principal Investigator Omar Pemberton | Bibi Khan |
|  | Hatem Elsharawih |
|  | Israel Okwor |
|  | Michael Gibbons |
|  | Najia Hasan |
|  | Nick Gill |
|  | Omar Pemberton |
|  | Ravishankar Punuloou |
| **Prince Philip Hospital** | Barnaby Hart |
| Principal Investigator Peter Havalda | Charlotte Jones |
|  | Emma Perkins |
|  | Joanne Connell |
|  | Kathryn Powell |
|  | Konara Dharmarathna |
|  | Laura Micusan |
|  | Linda Brien |
|  | Michael Martin |
|  | Peter Havalda |
|  | Richard Timoney |
|  | Robert Cassidy |
|  | Tracy Lewis |
|  | Yvonna Plesnikova |
| **Princess Royal University Hospital (PRUH)** | Ahmed Elfaioumy |
| Principal Investigator Karthick Duraisamy | Ananya Mandal |
|  | Caitlin Spooner |
|  | Clare Finney |
|  | Clare Donegan |
|  | Emma Clarey |
|  | Hayley Kaye |
|  | Humza Yusuf |
|  | Karthick Duraisamy |
|  | Liam Botterill |
|  | Mikaela Theocharidou |
|  | Mohamed Afifi |
|  | Nayer Guirguis |
|  | Nicola Griffiths |
|  | Osokoya Bosokoya |
|  | Pranav Pershad |
|  | Ranganathan Srinivasan |
|  | Ravi Bhatia |
|  | Sophie Rabas |
|  | Sruthi Ravichandran |
|  | Sylvia Martin |
| **Queen Elizabeth Hospital Gateshead** | Adam Cookson |
| Principal Investigator Joanne Knight | Emma Allen |
|  | Fiona Sim |
|  | Frederick Hett |
|  | Helen Wild |
|  | Ingvild Helgesen |
|  | James Mcpherson |
|  | Jenny Ritzema |
|  | Joanne Knight |
|  | Julie James |
|  | Nikhil Tambe |
|  | Rachel Lucas |
|  | Stephanie Berry |
|  | Usama Butt |
| **Queen Elizabeth Hospital, Birmingham** | Ali Usman |
| Principal Investigator Mansoor Bangash | Anandh Balu |
|  | Anna Musgrave |
|  | Anne Lim |
|  | Chiemezie Orji |
|  | David Desai |
|  | Despoina Terzi |
|  | Hoi Yan Wong |
|  | James Baker |
|  | Jamie Thompson |
|  | Jessica Mernagh |
|  | Jigneshbhai Patel |
|  | Julia Blackburn |
|  | Kavaldeep Jabbal |
|  | Liam Roberts |
|  | Mansoor Bangash |
|  | Menanta Van Velze |
|  | Mohyman El Habishi |
|  | Muneeba Ahmed |
|  | Neelesh Mohan |
|  | Neil Tiwari |
|  | Rupal Swami |
|  | Zikrullah Kalim |
| **Queen Elizabeth Hospital, Lewisham and Greenwich** | Allison Mascagni |
| Principal Investigator Danielle Factor | Charlotte Braithwaite-shirley |
|  | Chris Holt |
|  | Daniel Henderson |
|  | Danielle Factor |
|  | Jonase Mutetwa |
|  | Neisha Rhule |
|  | Pradnya Vadnere |
|  | Rachel Williams |
|  | Samia Pilgrim |
|  | Shirley Braithwaite |
| **Queen Elizabeth Queen Mother Hospital** | Dushyant Sharma |
| Principal Investigator Sanjay Agrawal | Eva Beranova |
|  | Gabriella Tutt |
|  | Hazel Ramos |
|  | Liam Austin |
|  | Sanjay Agrawal |
|  | Sharon Turney |
|  | Tracy Hazelton |
| **Queen Elizabeth Hospital, Kings Lynn** | Charlotte Kingsley |
| Principal Investigator Holy Sira | Harriet Mark |
|  | Holly Sira |
|  | Jessica Murley |
|  | Ping Chen |
|  | Shrestha Sinha |
| **Queen Victoria Hospital** | Cassandra Honeywell |
| Principal Investigator Fiona Ramsden | Catherine Bounds |
|  | Christopher Ward |
|  | Fiona Ramsden |
|  | Gail Pottinger |
|  | Julian Giles |
|  | Megan Thomas |
|  | Tom Hansen |
|  | Tracey Shewan |
| **Queens Hospital Burton** | Agha Isguzar |
| Principal Investigator Manab Haldar | Amara Masood |
|  | Bis Das |
|  | Caroline Dickens |
|  | Debasis Pradhan |
|  | Emily Blurton |
|  | Gillian Bell |
|  | Louise Wilcox |
|  | Manab Haldar |
|  | Precious Basvi |
|  | Sam Besant |
|  | Sunday Ekaiidem |
|  | Sunita Gurung |
| **Queens Hospital Romford** | Abishek Chitnis |
| Principal Investigator Madeep Phull | Daisy Riddle |
|  | Heidi Chandler |
|  | Louis Peakall |
|  | Mandeep Phull |
|  | Oliver Mckinney |
|  | Suraj Shah |
| **Raigmore Hospital** | Alex Reid |
| Principal Investigator Mario Fernandes | Alys Wei |
|  | Andrew Richardson |
|  | Ben Marshall |
|  | Frances Hines |
|  | Heather Turnbull |
|  | Kat Murray |
|  | Laura Mcilhatton |
|  | Mario Fernandes |
|  | Mark Hannen |
|  | Rebecca Trimble |
| **Robert Jones & Agnes Hunt Hospital** | Barbara Linklater-jones |
| Principal Investigator Melanie Bloor | Charlotte Perkins |
|  | Claire Nicholas |
|  | Claire Wright |
|  | Jayne Edwards |
|  | Johanna Wales |
|  | Julie Steen |
|  | Melanie Bloor |
|  | Sara Owen |
|  | Sarah Clamp |
|  | Teresa Jones |
|  | Tessa Rowlands |
|  | Theresa Garratt |
| **Rotherham General Hospital** | Andy Mitchell |
| Principal Investigator Elinor Cromarty | Becky Hawes |
|  | Cheryl Graham |
|  | Elinor Cromarty |
|  | Jake Mccormick |
|  | Lianne Sellors |
|  | Louise Weatherley |
|  | Morwenna Read |
|  | Namitha Jayaprabhu |
|  | Natalie Chan |
|  | Nick Hobbs |
|  | Prasan Kadaramandalgi |
|  | Rachael Faulkner |
|  | Rachel Walker |
|  | Rick Harrold |
|  | Sarah Ingram |
| **Royal Alexandra Hospital and Vale of Leven** | Alasdair Turnbull |
| Principal Investigator Michael Brett | Alice Solerod |
|  | Alistair May |
|  | Colin Hutchison |
|  | David Ure |
|  | Fenella Barlow-pay |
|  | Gary Paul |
|  | Iain MacTier |
|  | Louise Clark |
|  | Michael Brett |
|  | Michael Kerr |
|  | Paul Beggs |
|  | Paul Mcconnell |
|  | Rebecca Vere |
|  | Shashi Timalapur |
|  | Timothy Gray |
| **Royal Berkshire NHS Foundation Trust** | Amelia Robinson |
| Principal Investigator Richard Barnes | Camilla Jackson |
|  | Cecille Cadampog |
|  | Dan Kent |
|  | Dave Golding |
|  | Killian Donovan |
|  | Kinza Emmanuel |
|  | Kulpdeep Nijjar |
|  | Odhran Keating |
|  | Parminder Bhuie |
|  | Poppy Sellwood |
|  | Richard Barnes |
|  | Sabi Rai |
| **Royal Blackburn Hospital** | Andrew Lancaster |
| Principal Investigator Anuradha Kurvey | Anuradha Kurvey |
|  | Beverley Hammond |
|  | Georgina Sutcliffe |
|  | Jillian Fitchett |
|  | Sanjiv Sharma |
| **Royal Bolton Hopsital** | Aashish Koirala |
| Principal Investigator Peter Sandbach | Helen Dixon |
|  | Antonia Peilober-Richardson |
|  | Chris Dale |
|  | Elfateh Ibrahim |
|  | Patricia Hodgson |
|  | Peter Sandbach |
|  | Priyash Verma |
|  | Rachel Fletcher |
|  | Raphael Holmes |
| **Royal Bournemouth Hospital** | Amy Gribble |
| Principal Investigator James Walker | Debbie Branney |
|  | Faith Beecham |
|  | Heather Tiller |
|  | James Walker |
|  | Lindsay Rogers |
|  | Nina Barratt |
|  | Sally Pitts |
|  | Sarah Savage |
| **Royal Cornwall Hospital** | Benita Adams |
| Principal Investigator Claire Preedy | Cara Campbell |
|  | Charlotte Barker-kirby |
|  | Claire Preedy |
|  | Daniel Phillips |
|  | Eden Leaper |
|  | Elliot Edmund |
|  | Eve Fletcher |
|  | Evelina Russell |
|  | Jack Williams |
|  | Jemima Henstridge-blows |
|  | Octavia Smith |
|  | Ollie Ryan |
|  | Peter Thomas |
|  | Riyea Akhtar |
|  | Selina Roy |
|  | Shane Roy |
|  | Suzanne Dean |
|  | Virginija Vilkelyte |
| **Royal Derby Hospital** | Alison Fletcher |
| Principal Investigator Nagendra Prasad | Carly Mcdonald |
|  | Catherine Addleton |
|  | Charlotte Downes |
|  | Coral Smith |
|  | Corinne Paxton |
|  | David Daly |
|  | Emily Mignott |
|  | Fiona Scothern |
|  | Jeanette Allison |
|  | Julie Edmonds |
|  | Katie Large |
|  | Lisa Mayles |
|  | Liz Nadin |
|  | Maggie Langley |
|  | Melanie Hayman |
|  | Mercy Korley |
|  | Nagendra Prasad |
|  | Samia Hussain |
|  | Sarah Miller |
|  | Timothy Streets |
|  | Zita Ibatuliniene |
| **Royal Devon and Exeter Hospital** | Aaron Lavin |
| Principal Investigator Helen Gilfillan | Chris Gillett |
|  | Ellie Nelson |
|  | Francis Bonomaully |
|  | Gemma Clark |
|  | Helen Gilfillan |
|  | James Grant |
|  | Jo Wilson |
|  | Katie Flower |
|  | Kevin Windsor |
|  | Linda Park |
|  | Lisa Jones |
|  | Martha Belete |
|  | Megan Purchall |
|  | Peggy Fooks |
|  | Pei Jean Ong |
|  | Rebecca Dyar |
|  | Sophie Ashman |
|  | Tania Nightingale |
|  | Tom Hewitt |
|  | Vanessa Chiappa |
|  | Will Foers |
|  | Will Spencer |
|  | Zach Jeffery |
|  | Zahid Gilitwala |
| **Royal Gwent, the Grange and Nevill Hall Hospitals** | Alison Hare |
| Principal Investigator Jake Hartford-beynon | Anna Roberts |
|  | Anushka Sierarine |
|  | Charlotte Dunn |
|  | David Agombar |
|  | Hannah Hoskins |
|  | Jake Hartford-beynon |
|  | Kota Kumar |
|  | Maxine Nash |
|  | Sammy Sharif |
|  | Simran Kooner |
|  | Swyn Lewis |
|  | Zoe Bennetton |
| **Royal Hampshire County Hospital** | Emma Norman |
| Principal Investigator Kathleen Hempenstall | Jordan Dennis |
|  | Kathleen Hempenstall |
|  | Rachael Brooks |
|  | Sarah Davidson |
| **Royal Lancaster Infirmary** | Andrew Prior |
| Principal Investigator Corinne Rimmer | Aziza Aini |
|  | Corinne Rimmer |
|  | Craig Marshall |
|  | Helen Spickett |
|  | Hilary Thatcher |
|  | Jack Dalziel |
|  | Jayne Craig |
|  | Julie Le |
|  | Jyothis Manalayil |
|  | Karen Burns |
|  | Nurul El-Ruslan |
|  | Steve Peters |
|  | Sushma Paccha |
|  | Therese Kelly |
|  | Wael Abdelrhman |
| **Royal Liverpool and Broadgreen University Hospitals NHS** | Amanda Wood |
| Principal Investigator Richad Ramsaran | Andre Simons |
|  | Christy Ord |
|  | Claire Hennigan |
|  | Emilia Spodniewska |
|  | Fran Westwel |
|  | Francesco Ferraro |
|  | Georgia Williams |
|  | Hannah Davis |
|  | Hefin Llewellyn |
|  | Hema Thomas |
|  | Ibrahim Abdelkhalek |
|  | Jenny Kirkpatrick |
|  | Katelyn Aitchison |
|  | Kera Hainey |
|  | Laura Cureton |
|  | Lauren Greer |
|  | Peter Harding |
|  | Rasmeet Kainth |
|  | Richard Ramsaran |
|  | Sophie Holder |
| **Royal National Orthopaedic Hospital** | Amit Patel |
| Principal Investigator Rachel Baumber | Antony Finny |
|  | Charlotte Pratt |
|  | Esther Hanison |
|  | Finny Antony |
|  | Fiona Fitzgerald |
|  | Jackline Nkhoma |
|  | Nana Okine |
|  | Nnebuife Oji |
|  | Rachel Baumber |
|  | Trusha Halai |
| **Royal Surrey County Hospital** | Donna Sanga |
| Principal Investigator James Mckinlay | James Mckinlay |
|  | Jerik Verula |
|  | Natalia Michalak |
|  | Nicholas Maskell |
|  | Paula Carvelli |
| **Royal United Hospital, Bath** | Abigail Mann |
| Principal Investigator Lesley Jordan | Annete Moreton |
|  | Catherine Bressington |
|  | Charlotte Ekblad |
|  | Frances Parry |
|  | Gabrielle Evans |
|  | Jennifer Pullen |
|  | John Wright |
|  | Joyce Katebe |
|  | Katherine Lloyd-jones |
|  | Laura Evans |
|  | Lesley Jordan |
|  | Lidia Ramos |
|  | Lucy Howie |
|  | Melody Rich |
|  | Natalie Gaskell |
|  | Rachel Awan |
|  | Rachel Beer |
|  | Ronan Hanratty |
|  | Sarah Burnard |
|  | Sarah Hierons |
|  | Sophia Muschnik |
|  | Tobin Osicki |
|  | Tom Cloke |
|  | Tonia Clark |
|  | Wendy Duberry |
| **Royal Victoria Hospital, Belfast** | Adam Glass |
| Principal Investigator Kerry Featherstone | Adam Lowe |
|  | Amy O'donnell |
|  | Anastasia Mcbride |
|  | Blayne Mccann |
|  | Catherine Poots |
|  | Claire Mccaul |
|  | Emma Gardiner |
|  | Emma Sweeney |
|  | Hannah Cooper |
|  | Helen Mcgourty |
|  | Kerry Featherstone |
|  | Matthew Mcguckin |
|  | Ruth Mccrystal |
| **Royal Wolverhampton Trust** | Alex Villaplaza |
| Principal Investigator Asha Ramkumar | Amardeep Kulkarni |
|  | Antonella Meraglia |
|  | Asha Ramkumar |
|  | Beenish Bashir |
|  | Benedict Williams |
|  | Chakravarthy Tutika |
|  | Deepak Ravindran |
|  | Emma Jay |
|  | Iqra Jangda |
|  | James Haddock |
|  | Jyothi Avula |
|  | Kanika Daga |
|  | Katie Betts |
|  | Kesavan Dhamodaran |
|  | Lewis Davies |
|  | Mohamed Shariff |
|  | Nick Ledlie |
|  | Parvathy Nair |
|  | Philip Thomas |
|  | Prabjoyt Kler |
|  | Puja Sharma |
|  | Roma Kalaria |
|  | Ross Evans |
|  | Stephen Norris |
|  | Sumant Shanbhag |
| **Russells Hall Hospital** | Anser Ali |
| Principal Investigator Anser Ali | Lesley Jones |
|  | Sarah Stavert |
|  | Stacey Forsey |
|  | Thomas Wallbridge |
|  | Tony Anthony |
|  | Vishal Amin |
| **Salford Royal Hospital** | Alice Harvey |
| Principal Investigator Manjunatha Patel | Bethan Charles |
|  | Danielle Walker |
|  | Diane Lomas |
|  | Elena Aperti |
|  | Helen Cristensen |
|  | Jesse Oliver |
|  | Kris Sivarajan |
|  | Manjunatha Patel |
|  | Melanie Taylor |
|  | Sheryl Bell-rhone |
|  | Vicky Thomas |
| **Salisbury District Hospital** | Abby Rand |
| Principal Investigator Xantha Holmwood | Hayley Savage |
|  | Oliver King |
|  | Xantha Holmwood |
| **Scarborough and Bridlington Hospital** | Alexander Knighton |
| Principal Investigator Bejamin Chandler | Alexander Polding |
|  | Alison Turnbull |
|  | Anna Waine |
|  | Benjamin Chandler |
|  | James Sangma |
|  | Janine Mallinson |
|  | Jordan Toohie |
|  | Kate Quigley |
|  | Katie Howard |
|  | Kerry Elliott |
|  | Laith Alsaket |
|  | Laura Barman |
|  | Rachael Harrison |
|  | Tania Neale |
| **Sherwood Forest Hospitals (Kings Mill Hospital)** | Andra Baghiu |
| Principal Investigator Srinivas Magham | Camelia Goodwin |
|  | Cheryl Heeley |
|  | Donna Sowter |
|  | Helen Shirt |
|  | Jill Kirk |
|  | Kaytie Bennett |
|  | Leah Holloway |
|  | Lynne Allsop |
|  | Mandy Gill |
|  | Nigel Thorpe |
|  | Philip Buckley |
|  | Rachel Johnson |
|  | Sarah Turner |
|  | Srinivas Magham |
|  | Stephanie Pike |
|  | Susan Smith |
|  | Vaisakh Viswanathan |
|  | Wayne Lovegrove |
| **Shrewsbury and Telford Hospital NHS Trust** | David Stuckey |
| Principal Investigator Paul Jones | Louise Ting |
|  | Paul Jones |
|  | Richard Watson |
| **Southampton General Hospital** | Aldo Bibnamini |
| Principal Investigator Karen Salmon | Alice Baker |
|  | Alix Bird |
|  | Anna Foster |
|  | Aurore Gerrish |
|  | Belinda Roberts |
|  | Clare Bolger |
|  | Daniela Georgieva |
|  | David Baker |
|  | Diana Mondo |
|  | Edisa Xhani |
|  | Elisabeth Jarman |
|  | Erika Kovacs |
|  | Hannah Wardall |
|  | Helena Eagles |
|  | Imogene Hedges |
|  | Jonathan Biss |
|  | Karen Salmon |
|  | Kerry Thorpe |
|  | Kim Golder |
|  | Luke Bracegirdle |
|  | Maria Baggott |
|  | Matthew Morris |
|  | Michael Carter |
|  | Norma Diaper |
|  | Owen Gregory |
|  | Rachel Burnish |
|  | Rachel Schranz |
|  | Sandra Bartolomeu-pires |
|  | Stephanie Kirby |
|  | Susan Jackson |
|  | Thomas Purvis |
| **Southend University Hospital** | Aneta Oborska |
| Principal Investigator Aneta Oborska | Bridgett Masunda |
|  | Henna Sattar |
|  | James Jegard |
|  | Mohamed Eshmandi |
|  | Naima Khalk |
|  | Nigara Atayeva |
|  | Prisca Gondo |
|  | Sunil Shah |
|  | Swapna Kunhunny |
| **Southern Health & Social Care Trust** | Aidan Cullen |
| Principal Investigator Michael Jones and Laura McLoughlin | Alexandrina Todd |
|  | Alison Blair |
|  | Anastasia Solomou |
|  | Andrew Dunbar |
|  | Barry Mcconville |
|  | Catherine Yarr |
|  | Claire Steenson |
|  | Declan Love |
|  | Denise Mcfarland |
|  | Duncan King |
|  | Emma Doherty |
|  | Erik Lichnovsky |
|  | Garwei Ho |
|  | Grainne Mckendry |
|  | Jacek Sobocinski |
|  | Jack Carmichael |
|  | James Crockett |
|  | Joanne Wylie |
|  | Laura Mcloughlin |
|  | Laura Somerville |
|  | Manvi Singhal |
|  | Michael Jones |
|  | Michael Magee |
|  | Mohammed Zayan |
|  | Niranjana Mohan |
|  | Patricia McCaffrey |
|  | Peter Mcclung |
|  | Peter Merjavy |
|  | Rebecca Rooney |
|  | Ruby Crothers |
|  | Ruth Thornbury |
|  | Ruth Thonbury |
|  | Samantha Leung |
|  | Shane Donnelly |
|  | Tim Bennett |
| **Southmead Hospital, Bristol** | Benjamin Hillam |
| Principal Investigator Sarah Martindale | Chris Thorne |
|  | Hannah Matthews |
|  | James Marshall |
|  | James Matthams |
|  | Jerome Condry |
|  | Kate Bell |
|  | Kerry Smith |
|  | Lydia Osborne |
|  | Ottilie Lloyd-thomas |
|  | Peter Sykes |
|  | Richard Mason |
|  | Sam Scholes |
|  | Sarah Dolling |
|  | Sarah Martindale |
| **Southport and Ormskirk NHS Trust** | Abdul Alim Khan |
| Principal Investigator Abdul Alim Khan | Afeez Abderahman |
|  | Alveena Bilal |
|  | Amanda Adigwe |
|  | Anna Morris |
|  | Belal Yasin |
|  | Bethany Preece |
|  | Christopher Goddard |
|  | Claire Corless |
|  | Himashi Nawimana |
|  | Patricia Jenkins |
|  | Rebecca Seddon |
|  | Rohith Nayak |
| **St Johns Hospital** | Andrew Goddard |
| Principal Investigator Andrew Goddard | Bridget Podmore |
|  | Michael Kriger |
|  | Paul Purvis |
|  | Rebecca Lovett |
| **St Marys Hospital, Imperial** | Ajantheny Naguleswaran |
| Principal Investigator Kenneth Murray | Dharshini Rajasooriyer |
|  | Drew Harding |
|  | Emily Russell |
|  | Jonathan Dunne |
|  | Lloyd Nunag |
|  | Sulaimaan Haq |
|  | Tizzy Abraham |
| **St Richards Hospital** | Denise Szabo |
| Principal Investigator Emily Dana | Emily Dana |
|  | Emma Finlay |
|  | Erikka Siddall |
|  | Esther Towner |
|  | Grace Williams |
|  | Hannah Atkinson |
|  | Sharon Floyd |
|  | Sophie Clarke |
|  | Thomas Thompson |
|  | Yolanda Baird |
|  | Yvette Thirlwall |
| **Stepping Hill Hospital** | Ali Nawaz |
| Principal Investigator Petya Chalakova | Bishal Gautam |
|  | Hywel Garrard |
|  | Karen Cheung |
|  | Leigh Wilson |
|  | Malathi Jabanathan |
|  | Nicolas Short |
|  | Petya Chalakova |
|  | Rebecca Mills |
|  | Rohan Colaco |
|  | Thomas Moore |
|  | Ujala Khwaja |
| **Sunderland Royal Hospital** | Akhil Lakhani |
| Principal Investigator Sean Cope | Alexandria Cropp |
|  | Claire Agius |
|  | David Swetman |
|  | Edward Halvey |
|  | Emma Worth |
|  | Faisal Shiekh |
|  | Hannah Fairclough |
|  | Henry Tancred-holmes |
|  | Iain Dryburgh |
|  | Jack Shepard |
|  | James Durrand |
|  | Jennifer Hooper |
|  | Katie Burke |
|  | Lindsey Woods |
|  | Matthew Bickerton |
|  | Natalie Hickling |
|  | Nicholas Killips |
|  | Robert Hessell |
|  | Sabrina Kapur |
|  | Sarah Dawson |
|  | Sean Cope |
|  | Sophie Curtis |
|  | Tom Ballance |
|  | Tom Collis |
| **The Christie Hospital** | Adam Bulinski |
| Principal Investigator Jaya Nariani | Anhthony Murphy |
|  | Jaya Nariani |
|  | Parisa Cutting |
|  | Roman Mary-genetu |
|  | Suzanne Allibone |
| **The James Cook University Hospital** | Abigail List |
| Principal Investigator Charlotte Anderson | Charlotte Anderson |
|  | Dean Wilkinson |
|  | Harry Heaton |
|  | Kerry Colling |
|  | Leanne Wakes |
|  | Nicola Powley |
|  | Tracy Ruddick |
| **The Princess Alexandra Hospital** | Bibi Badal |
| Principal Investigator Rajamani Seturaman | Dinesh Das |
|  | Huw Griffiths |
|  | Joanne Finn |
|  | Karen Ixer |
|  | Michelle Grove |
|  | Nikki White |
|  | Patricia Nabayego |
|  | Rajamani Seturaman |
|  | Sophie Harris |
| **The Royal Glamorgan Hospital** | Alice O'donnell |
| Principal Investigator Neeta Taylor | Ameerah Azmil |
|  | Amy Nixon |
|  | Bethan Gibson |
|  | Catherine Archer |
|  | Catherine Farrelly |
|  | Ceri Lynch |
|  | Faye Bond |
|  | Iain Mclure |
|  | Kathryn Lloyd-thomas |
|  | Martin Cole |
|  | Nathan Anderson |
|  | Neeta Taylor |
|  | Nick Preston |
|  | Richard Roberts |
|  | Robert Jones |
|  | Tracey Thomas-wood |
| **The Royal London Hospital** | Aaroh Dubey |
| Principal Investigator Ashley Parker | Alison Li |
|  | Anna Yang |
|  | Asher Knight |
|  | Ashley Parker |
|  | Ben Thorp |
|  | Divya Harshan |
|  | Fatimah Bme |
|  | Hannah Nugent |
|  | Heather Fuller |
|  | Ioannis Kapsokalyuas |
|  | Jennifer Overend |
|  | Karen Collins |
|  | May Webb |
|  | Mina Arsan |
|  | Mira Razzaque |
|  | Nicky Lau |
|  | Philip Devendra |
|  | Xiaoyan Yang |
| **The Whittington Hospital** | Amy Findlay |
| Principal Investigator Denise Lim | Denise Lim |
|  | Emma Jenkins |
|  | Floju Chin |
|  | Kayleigh Gilbert |
|  | Lauren Booker |
|  | Lucy Blair |
|  | Nicola Brown |
| **Torbay Hospital** | Angie Foulds |
| Principal Investigator Johannes Retief | Gregory Warren |
|  | Helen Williams |
|  | Johannes Retief |
|  | Kieran Miller |
|  | Laura Helley |
|  | Pauline Aspa |
| **Tunbridge Wells Hospital** | Daniel Gorman |
| Principal Investigator Hilary Taylor | Emily Phiri |
|  | Fabio Fernandes |
|  | Gavin Fossey |
|  | Heather Callaghan |
|  | Hilary Taylor |
|  | Indrowtree Sookun |
|  | Jen Assimakopoulos |
|  | Miriam Davey |
|  | Rebecca Seaman |
|  | Vicky Earl |
| **University College Hospital** | Atokwame Ocansey |
| Principal Investigator Sohail Bampoe | Chimverly Diaz |
|  | Denise Wyndham |
|  | Ernesto Bettini |
|  | Gladys Martir |
|  | Maya Sussman |
|  | Sohail Bampoe |
| **University Hospital Ayr** | Amadeusz Ziarkowski |
| Principal Investigator Philip Hamilton | Cameron Taylor |
|  | Danielle Gilmour |
|  | Debbie Callaghan |
|  | Fiona Elliott |
|  | Mark Wilson |
|  | Philip Hamilton |
|  | Pritam Mohanty |
|  | Sharon Meehan |
|  | Stephen Wood |
|  | Toni Mcintosh |
| **University Hospital Coventry** | Adam Boulton |
| Principal Investigator Carol Bradbury | Anne Scase |
|  | Carl Groves |
|  | Carol Bradbury |
|  | Charlotte Bullock |
|  | Giles Coverdale |
|  | Jennifer Morrish |
|  | Katharine Reeves |
|  | Katie Ramm |
|  | Kay Mak |
|  | Michelle Aukland |
|  | Nalini Sethia |
|  | Sarah Delahunt |
|  | Stephen Pearson |
|  | Susanne Anver |
|  | Tom Radcliffe-law |
|  | Tom Trouton |
|  | Tom Wilson |
| **University Hospital Crosshouse** | Ali Meikle |
| Principal Investigator Andrew Clark | Amy Clark |
|  | Andrew Clark |
|  | Cara Hughes |
|  | Colin Pow |
|  | Conor Nichol |
|  | David Finn |
|  | Hugh Neill |
|  | Jacqueline Mccarthy |
|  | Jamie Weir |
|  | Jane Collie |
|  | Kathryn Mcaleer |
|  | Ker Wei |
|  | Laura Macdonnell |
|  | Mark Andonovic |
|  | Monika Doshi |
|  | Nick Brown |
|  | Phil Jacobs |
|  | Rob Bonar |
|  | Ross Junkin |
|  | Ruth Blackett |
|  | Sam Norman |
|  | Sarjit Singh |
| **University Hospital Hairmyres** | Alfie Lloyd |
| Principal Investigator Jonathan Edgar | Arthur Norton |
|  | Chloe Macdonald |
|  | Claire Young |
|  | Dairshini Sithambaram |
|  | Emma Lee |
|  | Euan Murdoch |
|  | Faith Dalgaty |
|  | Fiona Walker |
|  | Jonathan Edgar |
|  | Laura Meney |
|  | Leigh Hamilton |
|  | Louise Jamieson |
|  | Lynn Valentine |
|  | Michael Airlie |
|  | Ross Dryden |
| **University Hospital Lewisham** | Angharad Langdon |
| Principal Investigator | Cara Mclean |
|  | Elizabeth Herbert |
|  | Fay Riley |
|  | Hamish Jackson |
|  | Harpreet Gill |
|  | Josephine Priest |
|  | Kimberley Rhodes |
|  | Manju Agarwal |
|  | Rebecca Mitchell |
|  | Richard Crowson |
| **University Hospital Monklands** | Gail Fleming |
| Principal Investigator Samuel Maguire | Michael Rodger |
|  | Samuel Maguire |
|  | Tracy Baird |
| **University Hospital of North Durham** | Abigail Sachs |
| Principal Investigator David Hamilton | Ami Wilkinson |
|  | Andrea Kay |
|  | Arun Varghese |
|  | Chloe Gilchrist |
|  | Christopher Pennington |
|  | David Hamilton |
|  | Jacob Mcdermott |
|  | Jamie Greenwood |
|  | Jonathan Dennis |
|  | Joseph Haynes |
|  | Khalid Zahir |
|  | Lauren Ferguson |
|  | Mark Birt |
|  | Mary Leese |
|  | Melanie Kent |
|  | Noreen Kingston |
|  | Peter Standen |
|  | Stefanie Hobson |
|  | Vicki Atkinson |
|  | Victoria Allinson |
| **University Hospital Wales** | Amanda Skingle |
| Principal Investigator Margaret Coakley | Anna Gilfedder |
|  | Anna Pisarcyk |
|  | Ben Sharif |
|  | Brigitte Baxter |
|  | Charlotte Eglinton |
|  | Claire Bunce |
|  | David Manson |
|  | Deborah Mann |
|  | Eais Mehmood |
|  | Farzad Saadat |
|  | Gail Williams |
|  | George Pitchers |
|  | Gina Allen |
|  | Graham Picton |
|  | Hannah Saitch |
|  | Harris Beca |
|  | Hawys Evans |
|  | Hywel Evans |
|  | Ifan Patchell |
|  | Jana Anandarajah |
|  | Jennie Williams |
|  | Jo Krawczyk |
|  | Joseph Edwards |
|  | Josh Patch |
|  | Julia Parnell |
|  | Jyothi Srinivas |
|  | Kerry Paradowski |
|  | Laura Gray |
|  | Luca Galvani |
|  | Margaret Coakley |
|  | Martin Grigg |
|  | Matt Short |
|  | Matthew Forester |
|  | Natalya Acres |
|  | Nia Humphry |
|  | Nicola Ball |
|  | Nicola Jardine |
|  | Oluwafisayo Olabisi |
|  | Pete Rogers |
|  | Phil Molloy |
|  | Rachel Skinner |
|  | Russel Townsend |
|  | Sam Tyrell |
|  | Samuel Bird |
|  | Sara Turley |
|  | Shaun Oram |
|  | Sonal Lodhi |
|  | Sophie Foreman |
|  | Sophie Pooley |
|  | Tom Roberts |
|  | Zarah Paris |
| **University Hospitals Sussex NHS FT (Royal Sussex County Hospital, Princess Royal Hospital, Haywards Heath, Sussex Orthopaedic Treatment Centre)** | Afonso Sequeira |
| Principal Investigator Stuart White | Alison Porges |
|  | Caroline Humphreys |
|  | Denise Skinner |
|  | Gabriella Salvi |
|  | Jane Gaylard |
|  | Justyna Nowak |
|  | Kanmani Lakshmikantha |
|  | Kate May |
|  | Keely Stewart |
|  | Mel Smith |
|  | Melissa Thorburn |
|  | Miles Seavill |
|  | Patrick Haye |
|  | Patrick Hayes |
|  | Stuart White |
|  | Usman Jamil |
|  | Valentina Toska |
|  | Zdenka Cipinova |
| **Victoria Hospital, Kirkcaldy** | Ally Rocke |
| Principal Investigator Katie Hunter | Cara Mccahill |
|  | Emma Scahill |
|  | Jessica Mccleery |
|  | Katie Hunter |
|  | Mandy Couser |
|  | Olivia Groom |
|  | Patricia Cochrane |
|  | Sarah Galloway |
|  | Susan Fowler |
| **Warwick Hospital** | Ben Wilkinson |
| Principal Investigator Emert White | Charlie Hudson |
|  | Chloe Thomson |
|  | Claire Baldwin |
|  | Emert White |
|  | Hannah Wolfenden |
|  | Hooi Shin |
|  | Ifra Zahoor |
|  | Jonathan Finnity |
|  | Parag Shastri |
|  | Rakesh Khunti |
| **Watford General Hospital** | Barnaby Glover |
| Principal Investigator Nidhi Gautam | Camilla Zorloni |
|  | Chiara Ellis |
|  | David Mccretton |
|  | Dharshana Ranasinghe |
|  | Elvira Hoxha |
|  | Georgia Perkins |
|  | Harish Ningegowda |
|  | Jackie Evans |
|  | Jules Kho |
|  | Mary James |
|  | Mehul Raithatha |
|  | Melanie Claridge |
|  | Naila Zahoor |
|  | Nidhi Gautam |
|  | Priyanka Moon |
|  | Rajesh Shankar |
|  | Rebekah Mostyn |
|  | Rosie Cortaville |
|  | Sarah Loftus |
|  | Saul Sundayi |
|  | Sukanya Khan |
|  | Xiaobei Zhao |
| **West Cumberland Hospital** | Elena Grani |
| Principal Investigator Elena Grani | Hannah Craig |
|  | Melanie Scott-richardson |
|  | Rosemary Harper |
|  | Una Poultney |
|  | Wendy Armstrong |
| **West Middlesex University Hospital** | Amir Majid |
| Principal Investigator Mhairi Jhugursing | Amrinder Sayan |
|  | Daniel Stubbins |
|  | Farrah Akkouch |
|  | Filomena Liccardo |
|  | Harriet Wilson |
|  | Jonathan Fox |
|  | Louise Cloney |
|  | Marie-louise Svensson |
|  | Mhairi Jhugursing |
|  | Mary O'brien |
|  | Priyakam Chowdhury |
|  | Roya Movahedi |
|  | Wazir Salamut |
| **Western General Hospital** | Alistair Coleman |
| Principal Investigator Louise Peach | Elspeth Paterson |
|  | Emily Kidd |
|  | Faisal Jafar |
|  | Fiona Auld |
|  | Iain Slessor |
|  | Julie Baruah-young |
|  | Louise Peach |
|  | Mala Greamspet |
|  | Nina Park |
|  | Rebecca Gormley |
|  | Teodora Filipescu |
|  | Akila Visvanathan |
|  | Alison Smith |
|  | Chetan Pataki |
|  | Debra Chatterton |
|  | Edel Robbins |
|  | Harvey Dymond |
|  | Katrina Stallard |
|  | Lindy Murray |
|  | Robert Grecian |
|  | Susan Wilkinson |
| **William Harvey Hospital** | Angela Moon |
| Principal Investigator Kim Jemmett | Angela Munteanu |
|  | Emma Ingall |
|  | Heather Weston |
|  | James Rand |
|  | Jeffrey Tsang |
|  | John Cockcroft |
|  | Kim Jemmett |
|  | Max Rigaudy |
|  | Prathiban Kumar |
|  | Reanne Solly |
|  | Sam Mcferran |
|  | Sarah Stirrup |
|  | Victoria Clarke |
| **Withybush General Hospital** | Amir Mekael |
| Principal Investigator Sunita Agarwal | Deshan Wickramaarachchi |
|  | Edward Todd |
|  | Jolene Brooks |
|  | Lara Sabry |
|  | Mary O'regan |
|  | Michelle Edwards |
|  | Prasanth Ganesan |
|  | Sharmila Ajaratnam |
|  | Sunita Agarwal |
| **Worcestershire Acute Hospitals NHS Trust (Worcester Royal Hospital, The Alexandra Hospital and Kidderminster Treatment Centre)** | Aly-khan Makhani |
| Principal Investigator Emily Johnson | Antonia Stone |
|  | Cindy Persad |
|  | Emily Johnson |
|  | Hannah Puddy |
|  | Jack Lee |
|  | Joseph Swani |
|  | Mike Eager |
|  | Mukunder Patel |
|  | Omolola Afelumo |
|  | Prakash Vadukul |
|  | Sarah Clayton |
|  | Shahid Khan |
|  | Shailendra Krishniah |
|  | Suhas Hebri |
|  | Vatsala Padmanabhan |
|  | Vinayak Nirmalan |
|  | Zuzana Hutkova |
| **Worthing Hospital** | Amy Davis |
| Principal Investigator Patrick Thorburn | Ben Mcallister |
|  | Bjorn Birk |
|  | Carmen Laue |
|  | Carrie Ridley |
|  | Charlotte Quick |
|  | Chloe Hoskins |
|  | Chris Redburn |
|  | Dan Puntis |
|  | Hannah Shimmin |
|  | Heather Fox |
|  | Jamie Gibson |
|  | Jimmy Siu |
|  | Joanna Dearden |
|  | Jordi Margalef |
|  | Kat Ganly |
|  | Katrine Thorup |
|  | Kirsten King |
|  | Layla Brookfield |
|  | Linda Folkes |
|  | Lucy Brennan |
|  | Marek Perera |
|  | Mark Burgess |
|  | Masseh Yakubi |
|  | Mohamam Selim |
|  | Neil Botting |
|  | Nicholas Weston-smith |
|  | Nicole Martins |
|  | Oliver Harvey |
|  | Patrick Thorburn |
|  | Piyush Varma |
|  | Raquel Gomez-marcos |
|  | Sam Goodhand |
|  | Samira Green |
|  | Sarah Driver |
|  | Sarah House |
|  | Shane Weinmann |
|  | Todd Leckie |
|  | Vivienne Cannons |
|  | Will Southall |
| **Wrexham Maelor Hospital** | Ahmed Salem |
| Principal Investigator Anna Williams | Amelia Devine |
|  | Anna Williams |
|  | Andrew Lonsdale |
|  | Chaw Nandar |
|  | Chijioke Orji |
|  | Chris Littler |
|  | Daphne Gunness |
|  | Emma Mcivor |
|  | Gillian Bennett |
|  | Hope Worthington |
|  | Jane Stockport |
|  | Josh Exley |
|  | Luke Williams |
|  | Mohit Sethi |
|  | Myles Roach |
|  | Oliver Smith |
|  | Philip Metcalf |
|  | Rachel Bradley |
|  | Robert Jesty |
|  | Romy Peterson |
|  | Sara Murray |
|  | Thomas Abberton |
|  | Will Simpson |
| **Wrightington Hospital and Royal Albert Edward Infirmary** | Anne Evans |
| Principal Investigator Anthony Short | Anthony Short |
|  | Caroline Tierney |
|  | Claire Williams |
|  | Daivd Wilcock |
|  | Emma Robinson |
|  | George Metias |
|  | Joshua Cooper |
|  | Katherine Dowdall |
|  | Lauren Brown |
|  | Rebecca Smith |
|  | Sarah Liderth |
|  | Thomas Bedwell |
|  | Tracey Taylor |
|  | Valerie Parkinson |
| **Yeovil District Hospital** | Aashkaben Shah |
| Principal Investigator Agnieszska Kubiszpudelko | Agnieszska Kubiszpudelko |
|  | Alice Quayle |
|  | Alison Lewis |
|  | Ashraf Ahmed |
|  | Ayman Gouda |
|  | Jess Perry |
|  | Kate Beesley |
|  | Linda Howard |
|  | Matthew Hillier |
|  | Munnaza Irfan |
|  | Nigel Beer |
|  | Rachel Wood |
|  | Ramez Aziz |
|  | Sarah Board |
|  | Thomas Bennett |
|  | Tressy Pitt-kerby |
| **York Hospital** | Andrew Chamberlain |
| Principal Investigator Andrew Chamberlain | Claire Brookes |
|  | Daniel Greenwood |
|  | Ellis Bramall |
|  | Harriet Pearson |
|  | Heidi Redfearn |
|  | Julie Anderson |
|  | Kate Howard |
|  | Louise Martin |
|  | Nicola Marshall |
|  | Paul Brittain |
|  | Radu Chirvasuta |
|  | Sally Gilroy |
|  | Stephy Jose |
|  | Zoe Cinquina |
|  | Zoe Scott |
| **Ysbyty Glan Clwyd Hospital** | Alice O'docherty |
| Principal Investigator Venkat Sundaram | Amy Gullis |
|  | Andras Safranko |
|  | Angela Pye |
|  | Annette Bolger |
|  | Hossam Abdelmotaal |
|  | Hossam Helmy |
|  | Laurence Baker |
|  | Llinos Davies |
|  | Nathan Littley |
|  | Rachael Farnell |
|  | Rachel Manley |
|  | Venkat Sundaram |
|  | Victoria Garvey |
|  | Yehya Slim |
| **Ysbyty Gwynedd Hospital** | Bryn Ellis |
| Principal Investigator Chrisopher Bailey | Christopher Bailey |
|  | Donna Ward |
|  | Ellen Knights |
|  | Jasmine Peh |
|  | Jeannie Bishop |
|  | Lisa Roberts |
|  | Wendy Scrase |
| **Unknown** | Emma Perkins |
|  | Ahmed Abdelhadi |
|  | Lynn Abel |
|  | Suzanne Allibone |
|  | Emad Al-Washash |
|  | Alpha Anthony |
|  | Beenish Bashir |
|  | Hannah Baytree |
|  | Karen Beaumont |
|  | Austin Begbey |
|  | Sophie Berry |
|  | Emily Bevan |
|  | Parminder Bhomra |
|  | Jonathan Blake |
|  | Neil Botting |
|  | Charlotte Brathwaite-Shirley |
|  | Layla Brookfield |
|  | Julie Chadwick |
|  | Kwun Chan |
|  | Karen Cloherty |
|  | Jon Clouston |
|  | John Cockcroft |
|  | Shirley Cocks |
|  | Joanne Connell |
|  | Maria Corretge |
|  | Alexa Cox |
|  | Benjamin Cracknell |
|  | Thomas Craven |
|  | Kavit Dasari |
|  | Elizabeth Denman |
|  | Anna Devlin |
|  | Kate Dillon |
|  | Joe Edwards |
|  | Abdalla Eisazwi |
|  | Kamal El-Badawi |
|  | Zenira Elbasheer |
|  | Sofia Fiouni |
|  | Rebecca Flanagan |
|  | Marian Flynn-Batham |
|  | Linda Folkes |
|  | Aidan Fullbrook |
|  | Gillian Garden |
|  | Ben Goodman |
|  | Jonathan Goodship |
|  | Miguel Guerrero |
|  | Ramanan Gukathasan |
|  | Emma Gunter |
|  | Dashiell Hall |
|  | Rachel Hallam |
|  | Henry Harcourt |
|  | Matt Henwood |
|  | Natasha Hughes |
|  | Mohamed Imam |
|  | Dianne Jackson |
|  | Zachary Jeffery |
|  | Ancy John |
|  | Lijo Joy |
|  | Prasanth Kandepalli |
|  | Matthew Kearney |
|  | Heather Kelley |
|  | Stephanie Kirby |
|  | Edward Knights |
|  | Louis Koizia |
|  | Chethana Kossinnage |
|  | Kartik Kota |
|  | Scott Latham |
|  | Anna Leslie |
|  | Chiwen Lin |
|  | Kat Lloyd Jones |
|  | Annabel Lloyd-Thomas |
|  | Ruairidh Mackay |
|  | Rose Mackonochie |
|  | Asif Mahmood |
|  | Ben McAllister |
|  | Chris McKee |
|  | Emma McKenna |
|  | Alex Metcalfe |
|  | Angelo Milioto |
|  | Raksha Mistry |
|  | Selma Mohammed |
|  | Mirela Mukaj |
|  | Anthony Murphy |
|  | Sophia Muschik |
|  | Priya Nagaraj |
|  | Vinayak Nirmalan |
|  | Raymond Njafuh |
|  | Ahmed Osman |
|  | Sarah Packer |
|  | Diran Padiachy |
|  | Emily Pallister |
|  | YeeWee Pang |
|  | Amy Parekh |
|  | Na Hyun Park |
|  | Sophie Patterson |
|  | George Pitchers |
|  | Barbara Pryzsyz |
|  | Paul Purves |
|  | Umang Qazi |
|  | Samyuktha Raj |
|  | Tom Ratcliffe-Law |
|  | Tom Reevell |
|  | Morag Renton |
|  | Kat Rhead |
|  | Maxime Rigaudy |
|  | Natalie Rodden |
|  | Hannah Saitch |
|  | Mark Sandford |
|  | Selena Sehgal |
|  | Imran Shareiff |
|  | Jasmin Shearer |
|  | Rachel Shipsides |
|  | Michaela Sibsey |
|  | Rhona Sinclair |
|  | Sophia Strong-Sheldrake |
|  | Jeevan Subramaniam |
|  | Neena Suchdev |
|  | Seb Tanner |
|  | Emma Tanton |
|  | Chris Tattersall |
|  | Jacqui Taylor |
|  | Anna Te Water Naude |
|  | Philip Thomas |
|  | Alex Touze |
|  | Tor Tuckey |
|  | Sara Turley |
|  | Chidimma Ugonabo |
|  | Yuvashree Venkatesan |
|  | Lagath Wanigabadu |
|  | Christopher Ward |
|  | Annamaria Wilce |
|  | Caroline Wrey Brown |
|  | Roger Yau |
|  | Nur Zalkapli |
